# Epidermal cornification is preceded by the expression of a keratinocyte-specific set of pyroptosis-related genes

**DOI:** 10.1038/s41598-017-17782-4

**Published:** 2017-12-12

**Authors:** Julia Lachner, Veronika Mlitz, Erwin Tschachler, Leopold Eckhart

**Affiliations:** Research Division of Biology and Pathobiology of the Skin, Department of Dermatology, Medical University of Vienna, 1090 Vienna, Austria

## Abstract

The homeostasis of the epidermis depends on keratinocyte differentiation and cornification, a mode of programmed cell death that does not elicit inflammation. Here, we report that cornification is associated with the expression of specific genes that control multiple steps of pyroptosis, another form of cell death that involves the processing and release of interleukin-1 family (IL1F) cytokines. Expression levels of pro-inflammatory IL1A and IL1B and of the pyroptotic pore-forming gasdermin (GSDM) D were downregulated during terminal differentiation of human keratinocytes *in vitro*. By contrast, negative regulators of IL-1 processing, including NLR family pyrin domain containing 10 (NLRP10) and pyrin domain-containing 1 (PYDC1), the anti-inflammatory IL1F members IL-37 (IL1F7) and IL-38 (IL1F10), and GSDMA, were strongly induced in differentiated keratinocytes. In human tissues, these keratinocyte differentiation-associated genes are expressed in the skin at higher levels than in any other organ, and mammalian species, that have lost the epidermal cornification program during evolution, i.e. whales and dolphins, lack homologs of these genes. Together, our results suggest that human epidermal cornification is accompanied by a tight control of pyroptosis and warrant further studies of potential defects in the balance between cornification and pyroptosis in skin pathologies.

## Introduction

Terminal differentiation of epidermal keratinocytes culminates in cornification, a mode of programmed cell death which differs from apoptosis^1^. Recently, other forms of programmed cell death, most notably necroptosis and pyroptosis, have been characterized^2–4^. The mechanism of cornification has remained incompletely understood while the regulation of cell death in the epidermis has become of wide interest, not the least, because of its possible implications in skin diseases^5^. Recently, pyroptosis has emerged as a pro-inflammatory mode of necrotic cell death that depends on the activation of members of the caspase-1 family (caspases-1/4/5, CASP1/4/5) and leads to the release of cytokines of the interleukin (IL)-1 family (IL1F)^2,3^.

Pyroptosis is a form of programmed necrosis^2^, during which the content of cell is released through a permeabilized cell membrane. Pyroptosis is initiated either via LPS-sensing caspases-4 and 5 or via the activation of inflammasomes by “danger-associated molecular patterns” or “homeostasis-altering molecular processes”^3,6^. Inflammasomes consist of a sensor protein, such as Nod-like receptor (NLR) family pyrin domain containing 3 (NLRP3), NLRP1 or Absent in melanoma 2 (AIM2), the adaptor protein PYD and CARD domain containing (PYCARD)/ASC, and caspase-1. Upon activation, the proinflammatory caspases-1, 4, and 5 cleave the proforms of IL-1β and IL-18 and the proform of gasdermin D (GSDMD). The latter processing step separates the inhibitory C-terminal domain from the N-terminal domain which then forms pores in the plasma membrane. These gasdermin pores contribute to the release of IL-1α and IL-1β and ultimately cause disintegration of the cell^3^. Besides GSDMD, other members of the gasdermin family (GSDMA, GSDMB, GSDMC, and GSDME) can induce pore formation upon different initiation events^4^.

IL1F cytokines play central roles in the inflammatory reactions^7^. Seven members of this protein family act as proinflammatory agonists (IL-1α, IL-1β, IL-18, IL-33, IL-36α, IL-36β, IL-36γ) while four have anti-inflammatory roles (IL-1RN, IL-36RN, IL-37, IL-38)^7^. Proteolytic removal of a prodomain is necessary for the activation of many IL1F members^8^. The proforms of the prototypical IL1F cytokines, IL-1β and IL-18, are processed by caspase-1 during pyroptosis and, in distinct other settings, by other proteases either within or outside of cells^8^.

While many of the aforementioned processes have been characterized in cells of the immue systems, in particular in macrophages, there is also evidence for roles of IL1F proteins and pyroptosis in the epidermis. To list but a few of many important early findings, IL-1 activity was detected in normal and psoriatic skin^9,10^ and IL-1 β processing activity was detected in the stratum corneum^11^. More recently, deficiency of IL-1 receptor antagonist (IL-1RN) was reported to cause an autoinflammatory disease with skin involvement^12^, mutations of the interleukin-36-receptor antagonist (IL-36RN) were shown to cause generalized pustular psoriasis^13^, and expression of *IL36G* was reported to distinguish lesions of psoriasis vulgaris from those of other erythemato-squamous skin diseases, such as atopic dermatitis and lichen planus^14,15^. Among members of the gasdermin family, three gasdermin A homologs are expressed in the epidermis and skin appendages of the mouse, and mutations in *GSDMA3* cause alopecia^16–18^. The crystal structure of GSDMA3 was determined, serving as a model for other gasdermins including the pyroptotic pore-forming GSDMD protein^19^. Recently, we showed that the caspase-1 inhibitor CARD18, also known as ICEBERG^20^ is predominantly expressed during terminal differentiation of epidermal keratinocytes^21^.

In the present study, we aimed to determine the expression of IL1F and gasdermin genes in a defined keratinocyte differentiation model and to establish the comparative evolutionary trajectories for these gene families in mammals with and without effective cornification. Furthermore, we screened pyroptosis-related protein families for members with predominant expression in the skin. We provide evidence for normal keratinocyte differentiation-associated expression of specific IL1F cytokines and proteins related to pyroptosis.

## Results

### Gene expression analysis of IL-1 family cytokines

**Figure 1:**
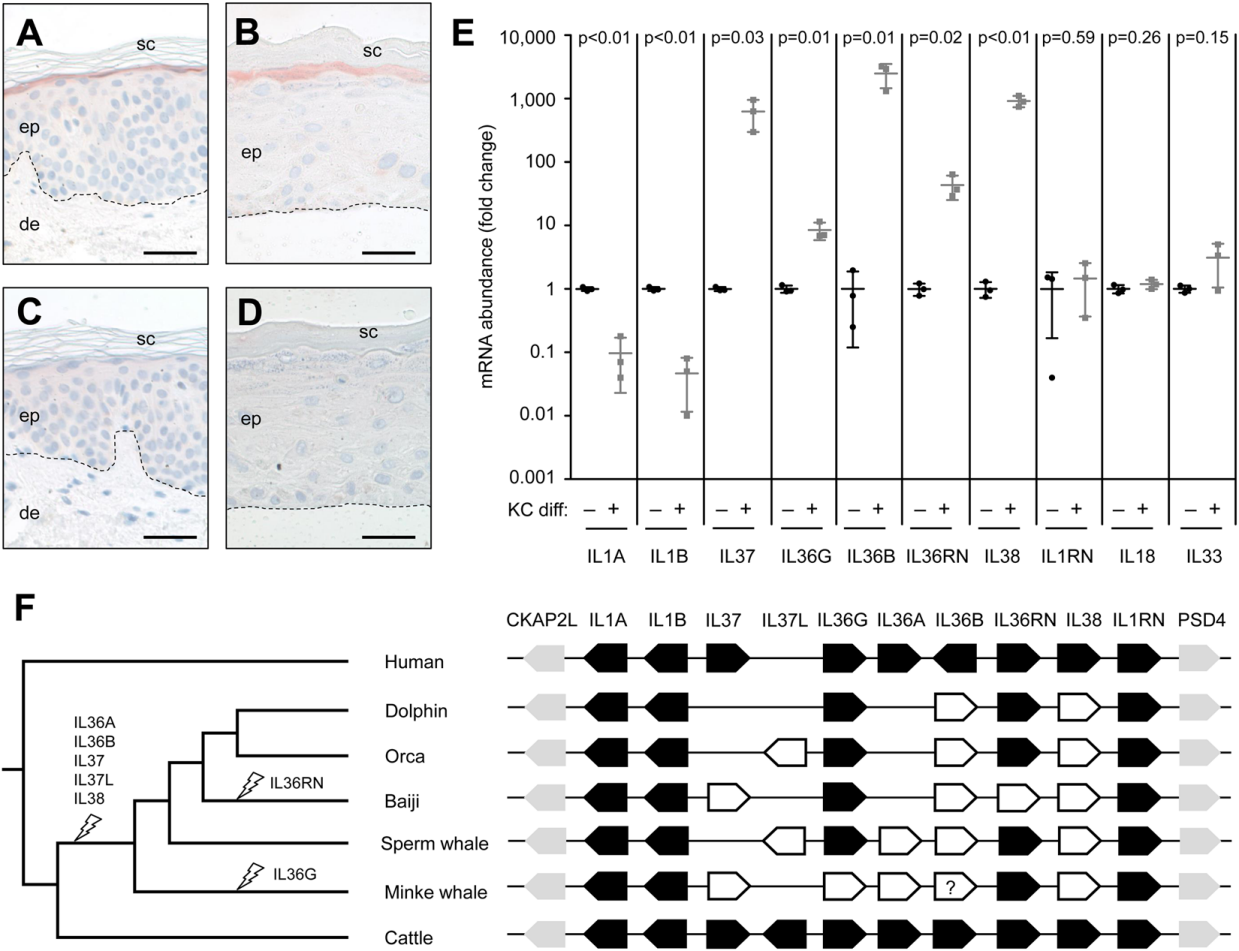
Gene expression analysis and comparative genomics of IL-1 family cytokines. The expression of IL37 was investigated by immunohistochemistry in normal human abdominal skin (**A**,**C**) and skin equivalents (**B**,**D**). Expression of IL37 is indicated by red staining (**A**,**B**). Replacing the primary antibody with an unrelated antibody abolished the staining and confirmed specifity (**C**,**D**). In addition, the specificity of the immunostaining was confirmed by siRNA-mediated knockdown of IL37, which abolished the staining (Suppl. Fig. S1). The data are representative of more than 3 experiments. de, dermis; ep, epidermis; sc, stratum corneum. Scale bars, 50 µm. (**E**) Quantitative RT-PCR analysis of IL1 family genes in human keratinocytes (KC) cultured *in vitro*. KC differentiation was either not induced (-) by maintaining cells in subconfluent monolayer culture or induced (+) by culture in skin equivalents (n = 3). Individual values are indicated by circles and squares; the horizontal line indicates the mean, and error bars indicate the standard deviation. Note the logarithmic scale of the mRNA expression level. The results shown here are representative of 4 experiments using KCs from different donors. p-values (t-test) are shown above the comparisons of expression levels with p < 0.05 being considered significant. (**F**) Phylogenetic tree and comparative analysis of the main IL1F gene locus, flanked by *CKAP2L* and *PSD4*, in humans, cetaceans, and cattle. White arrows indicate inactive gene remnants. A mutation close to the end of the coding sequence of *IL36G* in the minke whale may have a negative or neutral effect on the functionality of the encoded protein (indicated by a question mark). Strike symbols on the tree indicate gene inactivation events inferred from comparative genomics.

To validate a model system for the study of keratinocyte differentiation-associated gene expression, we determined the expression pattern of IL-37, an anti-inflammatory IL1F cytokine^22,23^, in human skin *in vivo*, as well as in 2-dimensional (monolayer) and 3-dimensional (skin equivalent) cultures of human keratinocytes *in vitro*. By immunohistochemistry, IL-37 was detected in the granular layer of the epidermis in normal human skin which was in agreement with published results obtained with another antibody^24^. IL-37 was also detected in the granular layer of skin equivalents (Fig. 1A–D). Treatment of keratinocytes with IL37-specific but not with control short interfering RNA (siRNA) abolished the immunohistochemistry signal (Suppl. Fig. S1). Reverse transcription-polymerase chain reaction (RT-PCR) analysis showed a predominant expression of IL37 variant 1, also referred to as IL-37b, which was reported to suppress inflammation^22^, at high levels in the epidermis and in skin models and only at low levels in keratinocytes in subconfluent culture (Suppl. Fig. S2). These data suggested that the skin equivalent model of keratinocyte differentiation induced a high level of *IL37* expression that was also detected in the granular layer of human epidermis.

Next we determined, by quantitative RT-PCR, the expression levels of all human IL1F genes in keratinocytes maintained in subconfluent monolayer culture and in skin equivalents. The mRNAs of *IL1A* and *IL1B* were significantly (p < 0.05) down-regulated whereas expression of *IL36B*, *IL36G*, *IL36RN*, *IL37*, and *IL38* was upregulated (Fig. 1E). The abundance levels of *IL18*, *IL1RN*, and *IL33* mRNAs were not significantly different in keratinocytes growing in subconfluent monolayer and skin equivalent cultures (Fig. 1E). *IL36A* mRNA was absent in undifferentiated keratinocytes and present at low levels in some but not all differentiated keratinocyte cultures. Comparison of gene expression levels in human tissues suggested that those genes, that were upregulated during keratinocyte differentiation, were expressed at higher levels in the skin than in any other organ of the human body (Suppl. Figs S3 and S4). These data indicated that an important part of the functions of *IL36B*, *IL36G*, *IL36RN*, *IL37*, and *IL38*, are associated with differentiated epidermal keratinocytes.

In previous studies, we and others have established comparative genomics as a method to correlate the presence or absence of genes with phenotypic adaptations of mammalian species^25,26^. Genes required specifically for a single biological process are conserved in a functional form when this process is active in a given species, whereas such genes tend to accumulate inactivating mutations in species that do not depend on this process. The terminal differentiation and cornification program of epidermal keratinocytes has degenerated during the evolutionary land-to-water transition of cetaceans (dolphins and whales)^25,27,28^ because these fully aquatic mammals do not need a barrier to a dry environment. We compared the gene loci of IL1F genes in humans, cetaceans and the cattle (*Bos taurus*, Artiodactyla), i.e. the phylogenetically closest relative of cetaceans, of which the genome sequence has been determined^29^ (Fig. 1F; Suppl. Table S1). The IL1F gene cluster of the cattle is largely syntenic with that of humans, however, there is an additional *IL37-like* gene in the cattle (Fig. 1F). By contrast, cetaceans lack functional orthologs of *IL36A*, *IL36B*, *IL37*, and *IL38* while other IL1F genes were conserved (Fig. 1F; Suppl. Fig. S5). The presence of an in-frame stop codon at the same position of the *IL38* gene in all cetaceans suggest that the gene was inactivated in a common ancestor prior to phylogenetic diversification of this clade (Suppl. Fig. S6). The mutations in other IL1F genes appear to be compatible with both a single, early gene loss event (as indicated in the phylogenetic tree on the left of Fig. 1F) and inactivations of these genes after the split of cetacean lineages. This pattern of presence and absence of genes indicates that *IL36A*, *IL36B*, *IL37*, *IL37-like*, and *IL38* were inactivated during or after the land-to-water transition, most likely in close association with the loss of the epidermal cornification program in cetaceans. Notably, the set of IL1F genes lost in cetaceans (Fig. 1F) included all human IL1F orthologs (*IL36B*, *IL37*, and *IL38*) that were upregulated more than a 100-fold during keratinocyte differentiation (Fig. 1E).

### Gene expression analysis and phylogenetic profiling of gasdermins

**Figure 2:**
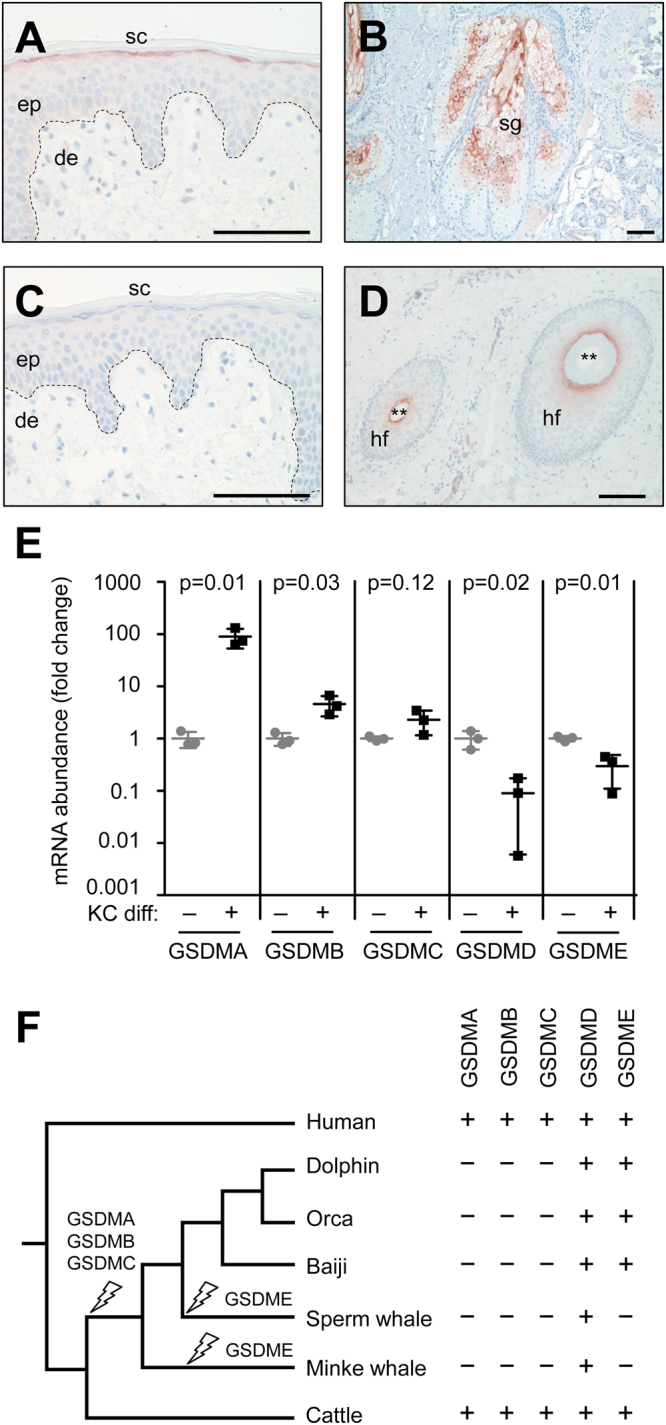
Gene expression analysis and comparative genomics of gasdermins. The expression of gasdermin A (GSDMA) was investigated by immunohistochemistry in normal human skin (**A**), sebaceous glands (**B**), and hair follicles (**D**). Tissue samples were from the thigh (**A**,**C**,**D**) and head (**B**). Expression of GSDMA is indicated by red staining. Replacing the primary antibody with an unrelated antibody abolished the staining and confirmed specifity (**C**). In addition, the specificity of the immunostaining was confirmed by siRNA-mediated knockdown of GSDMA, which abolished the staining (Suppl. Fig. S9). The data are representative of more than 3 experiments. de, dermis; ep, epidermis; hf, hair follicle; sg, sebaeous gland; sc, stratum corneum. Scale bars, 100 µm. (**E**) Quantitative RT-PCR analysis of gasdermin genes in human keratinocytes (KC) cultured *in vitro*. KC differentiation was either not induced (−) by maintaining cells in subconfluent monolayer culture or induced (+) by culture in skin equivalents (n = 3). Individual values are indicated by circles and squares; the horizontal line indicates the mean, and error bars indicate the standard deviation. The results shown here are representative of 4 experiments using KCs from different donors. p-values (t-test) are shown above the comparisons of expression levels with p < 0.05 being considered significant. (**F**) Phylogenetic tree and comparative analysis of conservation of gasdermin genes in humans, cetaceans, and cattle. Presence and absence of functional gene orthologs is indicated by “+” and “−”, respectively. Strike symbols on the tree indicate gene inactivation events inferred from comparative genomics.

Gasdermins are a group of pore-forming proteins among which gasdermin D has been shown to mediate pyroptotic cell death^3,19,30,31^. Three gasdermin A isoforms of the mouse have been reported to be expressed in the skin and the esophagus^18^. Immunohistochemistry showed that human GSDMA is expressed in the granular layer of normal epidermis, in differentiated sebocytes of the sebaceous glands, and in the inner root sheath of the hair follicle but not in undifferentiated keratinocytes or non-epithelial cells of the skin (Fig. 2A–D).

We performed quantitative RT-PCR analyses of gasdermins A through E in monolayer versus skin equivalent cultures of human keratinocytes. *GSDMA* and, to a lesser extent, *GSDMB* were upregulated in their expression whereas *GSDMD* and *GSDME* were downregulated and *GSDMC* levels were not significantly changed during differentiation (Fig. 2E). Among all human tissues, for which consolidated gene expression levels are available, the skin was the site of maximal expression of *GSDMA* while other gasdermins were either expressed at similar levels in multiple tissues or predominantly in organs other than the skin (Suppl. Fig. S7).

Comparative genomics showed conservation of GSDMD and inactivation of the GSDMA, GSDMB, GSDMC and GSDME genes in cetaceans (Fig. 2F; Suppl. Fig. S8; Suppl. Table S2). All gasdermin genes were conserved in the cattle (Fig. 2F; Suppl. Table S2) while the mouse has been reported to have 1 *GSDMD*, 3 *GSDMA* and 4 *GSDMC* but no *GSDMB* genes^32^. Thus, cetaceans have a remarkably reduced panel of gasdermins.

As the pore-forming activity of gasdermin D is implicated in the pyroptotic death of macrophages^30,33^ and gasdermin A is specifically expressed shortly before programmed death (cornification) of keratinocytes, we tested the hypothesis that gasdermin A might be required for the differentiation-associated death of epidermal keratinocytes. To this end, we knocked down by specific siRNAs the expression of GSDMA in keratinocytes *in vitro* (Suppl. Fig. S9) and compared the morphology of skin models formed by normal GSDMA-positive and GSDMA-negative keratinocytes. Despite efficient suppression of GSDMA expression (Suppl. Fig. S9A–C), keratinocytes cornified and formed an orthokeratotic (nuclei-free) stratum corneum (Suppl. Fig. S9D,E), suggesting that gasdermin A is not required for terminal differentiation-associated death of human keratinocytes.

### RT-PCR analysis and comparative genomics of other pyroptosis-related genes

**Figure 3:**
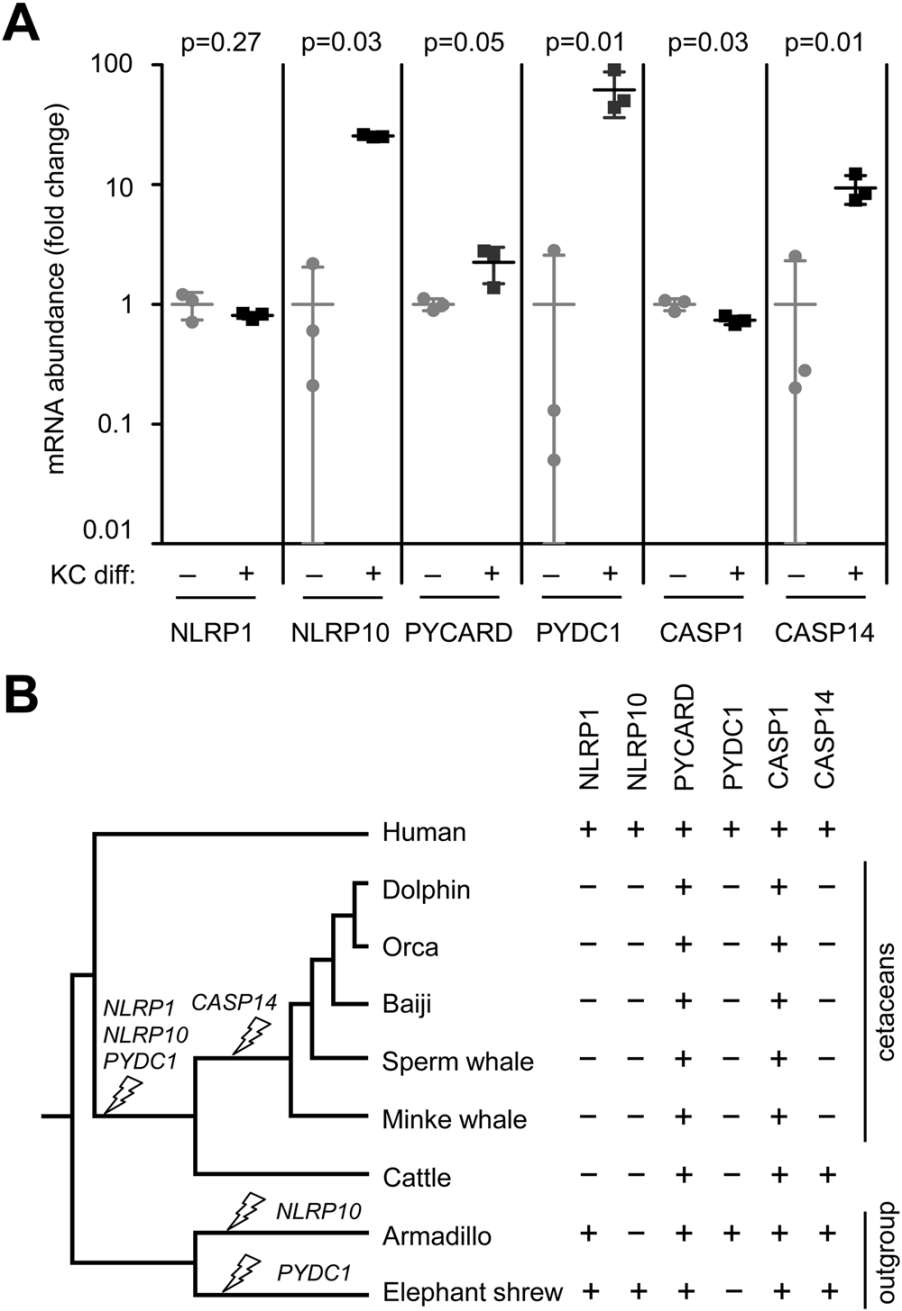
Gene expression analysis and comparative genomics of other pyroptosis-related proteins. (**A**) Quantitative RT-PCR analysis of genes, that encode pyroptosis regulator and effector proteins in human epidermal keratinocytes (KC). KC differentiation (KC diff) was either not induced (−) by maintaining cells in subconfluent monolayer culture or induced (+) by culture in skin equivalents (n = 3). The results shown here are representative of experiments using KCs from two donors. p-values (t-test) are shown above the comparisons of expression levels with p < 0.05 being considered significant. (**B**) Phylogenetic analysis of selected pyroptosis genes in humans, cetaceans, cattle, armadillo, and elephant shrew. The latter two species were chosen to represent an outgroup (Atlantogenata) in which an ortholog of each of the six indicated genes is present in at least one species, thus indicating inheritance from a common ancestor of all placental mammals. Presence and absence of functional gene orthologs is indicated by + and -, respectively. Strike symbols on the tree indicate gene inactivation events inferred from comparative genomics.

As the prototypical members of the IL1 and gasdermin protein families participate in the same molecular pathway, linking the sensation of intracellular danger or damage (e.g., via NLRPs) to proteolysis, membrane rupture and cytokine release, we extended our investigation of keratinocyte differentiation-dependent gene expression to other confirmed and potential factors in the afore-mentioned reaction cascade. Screening gene expression levels in human tissues suggested that *NLRP10*, *PYDC1*, and *CASP14* are predominantly expressed in epidermal keratinocytes (Suppl. Fig. S10). RT-PCR analyses showed that the expression of *NLRP1* remained constant whereas *NLRP10* was strongly upregulated during differentiation of keratinocytes (Fig. 3A). The mRNA level of *PYCARD* was slightly increased whereas that of the closely related *pyrin domain containing 1* (*PYDC1*), which encodes an anti-inflammatory pyrin domain-only protein^34^, was strongly upregulated during differentiation of keratinocytes (Fig. 3A). The mRNA level of the pyroptosis-mediating and IL-1 beta processing protease caspase-1 (*CASP1*) was slightly decreased when keratinocytes were induced to differentiate whereas the expression of the related *CASP14* was strongly increased in skin models relative to proliferating keratinocytes (Fig. 3A). The latter result confirmed our previous report in which caspase-14 was identified as the only caspase transcriptionally upregulated during keratinocyte differentiation^35^. All genes, that were either up- or downregulated in skin equivalent cultures (Figs 1E, 2E and 3A), showed the same direction of change in expression levels when keratinocyte differentiation was induced by postconfluent culture (Suppl. Fig. S11), however, some changes were not statistically significant under those conditions.

Comparative genomics showed that *NLRP10*, *PYDC1*, and *CASP14*, which are transcriptionally upregulated during human keratinocyte differentiation, lack functional orthologs in cetaceans (Fig. 3B; Suppl. Fig. S12). The species distribution of these genes, including presence in basal placental mammals, suggests that these genes were present in an ancestor of cetaceans. However, only *CASP14* was lost specifically in cetaceans^25^ whereas *NLRP10* and *PYDC1*, which are not conserved in several mammalian lineages (our unpublished data), were likely lost in a common (terrestrial) ancestor of cattle and cetaceans (Fig. 3B).

**Figure 4:**
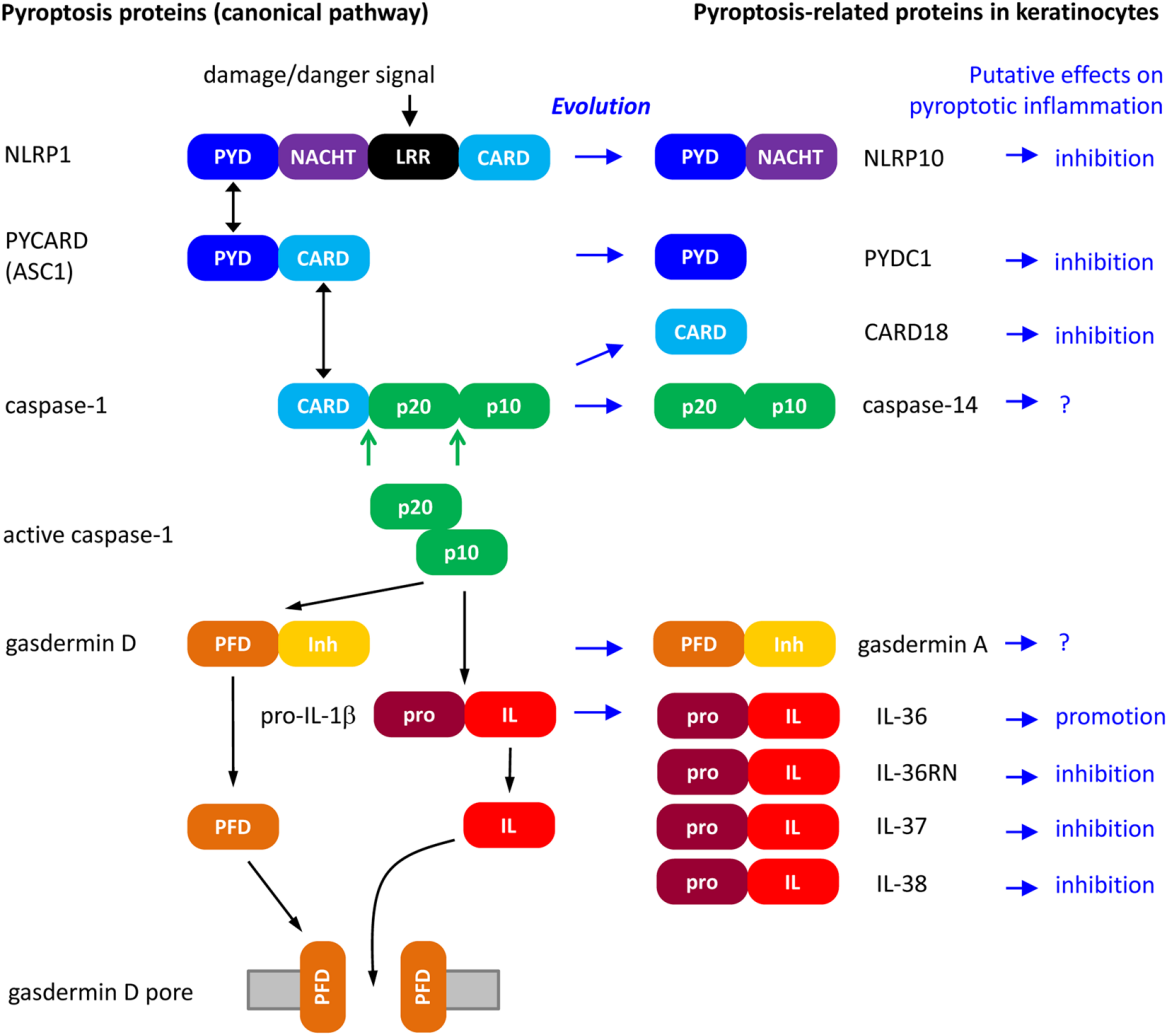
Pyroptosis-related gene families contain members that are predominantly expressed in differentiated epidermal keratinocytes and whose orthologs are dispensable for cetaceans. The left part of the scheme depicts major regulators of pyroptosis and their interactions leading from the sensing of damage/danger signals to caspase-1 activation, proteolysis of pro-IL-1β and gasdermin D, and ultimately pore formation in the cell membrane and release of IL-1β. On the right, evolutionarily related proteins, that are predominantly expressed in keratinocytes, are shown and their effects on pyroptotic inflammation, mostly reported by other cell types, are indicated. The domain organization of the proteins is schematically shown. CARD, caspase recruitment domain; IL, interleukin 1; Inh, inhibitory domain; LRR, leucine-rich repeats; NACHT, NACHT domain; p10, caspase domain of approximately 10 kD; p20, caspase domain of approximately 20 kD; PFD, pore forming domain; pro, prodomain; PYD, pyrin domain.

Taken together, the results of this study indicate that several members of the pyroptosis and IL1-signaling-related gene families are transcriptionally upregulated during human keratinocyte differentiation and dispensable for mammalian species with incomplete epidermal cornification (Fig. 4).

## Discussion

The results of this study suggest that the terminal differentiation program of human keratinocytes involves the expression of specific members of gene families implicated in pyroptosis and inflammatory signaling. While some of these genes had been linked to epidermal differentiation in previous studies^18,35^, *PYDC1* is newly identified as a potential factor in keratinocyte differentiation. Moreover, the comprehensive screening of genes has allowed to demonstrate that for virtually every level of the pyroptosis activation and signaling cascade, there is a protein homolog predominantly expressed in differentiated keratinocytes. Thereby, our results suggest that unique mechanisms of control for pyroptosis or pyroptosis-like processes exist in the epidermis.

Our results confirm and extend the concept of high expression levels of several IL1F genes in the skin^7^. In previous studies, changes in the expression of IL1Fs and in particular IL-36 cytokines were mainly found in association with inflammatory skin diseases^22,23,35–39^. *IL37* was reported to be expressed in many tissues including the epidermis^22,40^. RNA-seq-based expression levels in human tissues (Suppl. Figs S3 and S4) and our demonstration of differentially upregulated IL1F gene expression in a defined *in vitro* skin model suggest that *IL36B*, *IL36G*, *IL36RN*, *IL37*, and *IL38*, are predominantly expressed in epidermal keratinocytes that are destined to undergo cornification. IL-37 as well as IL-36RN and IL-38 have been implicated in anti-inflammatory responses^22,23,37,38^ whereas IL-36A, B, and G are proinflammatory proteins^39^. It appears possible that the high expression levels of IL-37 contribute to the suppression of pro-inflammatory signaling in normal human epidermis^24,40–42^. Further studies of IL-37 regulation and signaling induced by IL-37 in the epidermis are required to fully uncover its role in human skin.

The immunohistochemical detection of IL-37 in the stratum granulosum was in agreement with previously reported data that were obtained with a different IL-37 antibody^24^ but differed from a reported staining with yet another antibody^40^. IL37-specific knockdown experiments in skin models suggested that our antibody gave specific signals in immunohistochemistry (Suppl. Fig. S1) whereas both specific and unspecific signals were obtained in western blot analysis (Suppl. Figs S13, S14). Presence of side products on western blots was not linked with appearance of immunohistochemical signals (Suppl. Fig. S14). Interestingly, IL-37 was also detected by proteomics in human stratum corneum^43^ where immunohistochemistry typically does not give signals due to masking of epitopes. IL36G was the only other IL1F member found in this proteomics study, while caspase-14 was the only caspase and GSDMA was the only gasdermin detected in the stratum corneum (see Table E1 in the online repository of ref.^43^).

Since the data on gene expression levels in human tissues and cells suggested primary roles of some IL1F genes in differentiated epidermal keratinocytes, we hypothesized that orthologs of these genes would confer an evolutionary advantage in species that have retained keratinocyte differentiation but not in species that have lost keratinocyte cornification, i.e. cetaceans^25,44^. Indeed, comparative genomics provided evidence for dispensability and loss of *IL37* as well as *IL38*, *IL36A* and *IL36B* in cetaceans. By contrast, the conservation of *IL36G* and *IL36RN* in cetaceans suggests that these IL1F genes have advantageous functions even in the absence of epidermal cornification. The above-mentioned hypothesis does not imply that all genes lost in cetaceans were originally involved in keratinocyte differentation, and it does not exclude that individual genes predominantly expressed in cornifying keratinocytes are lost in species in which keratinocyte cornification is still active. The latter point is exemplified by the loss of *IL37* in a subclade of rodents including the mouse (Suppl. Fig. S15). Thus, the results of comparative genomics are compatible with a largely tissue-specific and, in some species, redundant role of *IL37* in the cornifying epidermis.

Gasdermin A is expressed in human epidermis, hair follicles and sebaceous glands, which apparently corresponds to the expression sites of the 3 murine GSDMA homologs^17,18^. Knockdown of GSDMA did not abrogate the ability of keratinocytes to undergo cornification and to degrade the nucleus *in vitro*, suggesting that this protein is not essential for the investigated aspects of cornification. However, a contribution of GSDMA to normal cornification cannot be excluded because GSDMB and GSDMC are also expressed in human keratinocytes and may functionally replace GSDMA when the latter is knocked down. Interestingly, GSDMA, GSDMB, and GSDMC are dispensable for cetaceans which have lost the epidermal differentiation program. The expression of GSDMA in differentiated cells of the sebaceous glands, as demonstrated by immunohistochemistry, suggests that human GSDMA does not function specifically in cornification. Sebocytes undergo a special mode of differentiation-associated programmed cell death that results in the holocrine secretion of sebum^45^. It will be interesting to immuno-localize GSDMA at the ultrastructural level and to investigate whether GSDMA forms pores in the plasma membrane or organellar membranes of differentiated keratinocytes or sebocytes. Recently, a polymorphism in the human *GSDMA* gene leading to an amino acid change (R18Q) was shown to be associated with systemic sclerosis, a severe autoimmune disease involving fibrosis of the skin and other organs^46^. In the context of the emerging key role of GSDMD in pyroptosis and the associations of GSDM family members with human diseases further studies into the regulation of GSDM proteins in the skin and other organs are warranted.

Besides epidermis-associated IL1F and gasdermin genes, further genes related to other pyroptosis proteins are expressed predominantly in the epidermis and, in contrast to the conservation in the human genome, they are absent in cetaceans. *NLRP10*, identified as susceptibility locus for atopic dermatitis in a genome-wide association study^47^, was reported to be expressed in keratinocytes in at least one publication^48^ whereas other reports have suggested roles of NLRP10 in other cell types^49,50^. Our results support a predominant function of NLRP10 in epidermal keratinocytes. NLRP10 lacks the carboxy-terminal leucine-rich domain that is otherwise conserved in NLRPs. Deletion of NLRP10 did not have an effect on an irritant contact dermatitis model but reduced inflammation in a contact hypersensitivity model in the mouse^47^. Our comparative genomics data indicate that NLRP10 as well as NLRP1 have been lost in a common ancestor of cetaceans and cattle, indicating that its function was dispensable or even disadvantageous. As this ancestor had a terrestrial lifestyle, loss of NLRP10 was likely not associated with a change in the epidermal differentiation program but perhaps with the regulation of immune responses to pathogens.

*PYDC1* (*POP1*, *ASC2*) was reported to be expressed in several other organs whereas expression was not investigated in the skin^51^. Recently, a regulatory role of PYDC1 in immune cells has been proposed^34^. Our gene expression results suggest an epidermal keratinocyte-associated function of PYDC1 and warrant further investigations of PYDC1 functions in the skin. Interestingly, a study of psoriasis-associated changes in the skin transcriptome showed upregulation of *PYDC1* and *PYCARD* in psoriasis lesions^52^. In the mouse, deletion of *PYCARD* (*ASC*) reduces contact hypersensitivity^53^, however, like cetartiodactyls (Fig. 3), this model species lacks a *PYDC1* ortholog. Our results indicate that human keratinocytes induced to differentiate *in vitro*, are a suitable model for investigating the functions of endogenous *PYDC1* in future studies.

Proinflammatory caspases have crucial roles in pyroptosis and, remarkably, a caspase related to the pyroptosis initiators, caspase-1, 4, and 5, is expressed specifically in cornifying keratinocytes. *CASP14* expression is upregulated during keratinocyte differentiation, as demonstrated by our present and previous results^35^, whereas expression of *CASP1* is not increased. Previously, we have also found that *CASP4* is not upregulated and *CASP5* is not expressed in differentiated normal epidermal keratinocytes^35^. As the expression of *CASP5* can be induced by LPS^54^ and immunochemical detection of caspase-5 protein in keratinocytes has been reported^55^, the roles of classical proinflammatory caspases in keratinocytes require further studies. Caspase-14 is phylogenetically closely related to the pyroptosis initiator caspases and caspase-15, a pyrin domain-containing caspase lost during human evolution^56,57^. In agreement with molecular phylogenetics, caspase-14 has a peptide substrate preference most similar to that of proinflammatory caspase-1^58^ but it is also able to cleave the inhibitor of caspase-activated DNase (ICAD) *in vitro* ^59^. Caspase-14 lacks a prodomain homologous to either the pyrin domain of caspase-15 or CARD of caspases-1, 4, and 5, suggesting that it does not integrate into inflammasomes, and *Casp14* gene knockout studies in mice have suggested a critical role of caspase-14 in the proteolytic processing of filaggrin^60^. Thus, the current evidence indicates that caspase-14 evolved by the cooption of an inflammation-associated ancestral caspase into a keratinocyte-specific subprocess of cornification.

Although this study was aimed at identifying genes that are upregulated in their expression during terminal differentiation of keratinocytes, we have also obtained evidence that several members of the gene families under investigation were downregulated in this process. The mRNA levels of *IL1A*, *IL1B*, and *GSDMD*, all of which play proinflammatory roles, decreased significantly during formation of skin equivalents *in vitro*. Based on the comparison of real time PCR cycle threshold (ct) values (Suppl. Fig. S16), we estimate that the expression levels of *IL1B* in undifferentiated keratinocytes in subconfluent culture were similar to those of *IL37* in differentiated keratinocytes in skin equivalents. The further evaluation of the relative impact of individual IL1F and pyroptosis-related genes on epidermal inflammation should include studies in which their expression is quantified at the single cell level and the activation of the encoded proteins by proteolytic processing is determined.

Taken together, the results of this study reveal that several genes, similar to established regulators and executors of pyroptosis, are expressed at high levels and in an almost exclusive manner in terminally differentiating keratinocytes. These data warrant further investigations into the functional roles of these genes during normal and disease-related keratinocyte cornification.

## Materials and Methods

### Ethics statement

The Ethics Committee at the Medical University of Vienna approved the use of skin samples that were obtained from plastic surgery for all experiments performed in this study (EK2011/1149). All donors provided written informed consent. All methods were performed in accordance with the relevant guidelines and regulations.

### *In vitro* culture of keratinocytes and skin models

Keratinocytes and fibroblasts were isolated from fresh skin samples (abdomen, thigh and upper arm) obtained from plastic surgery. The epidermis and dermis were separated by incubation with 2.4 U/ml dispase (Roche Applied Science, Basel, Switzerland) at 4 °C overnight. Subsequently, keratinocytes were isolated from the epidermis by incubation with trypsin (Lonza, Basel, Switzerland) for 8 min at 37 °C and incubation with DNase 1. Keratinocytes were cultured in KGM2 medium (Lonza). Fibroblasts were isolated from the dermis by incubation with collagenase (Thermo Fisher Scientific, Waltham, MA) at 37 °C for 1 h. Fibroblasts were cultured in penicillin/streptomcycin-supplemented DMEM (Thermo Fisher Scientific) with fetal bovine serum (Biochrom AG, Berlin, Germany). Cells were maintained in monolayer cultures or used for the preparation of organotypic skin cultures (skin equivalents) according to published protocols with the modification that KGM2 was used instead of KGM^61^. Knock-down experiments with Stealth™ siRNAs (Thermo Fisher Scientific) were performed as described previously^61^. Briefly, keratinocytes were transfected with 80 µl siRNA (20 µM) in the presence of 50 µl Lipofectamine 2000. Three Stealth™ siRNAs specific for gasdermin A (RNA duplex sense sequences: GSMDA siRNA1: 5′-CAGAGCUAAGUGAAGCCCAACAGAA-3′, GSMDA siRNA2: 5′-GGAUAUUCCACAUAUCUGCAAUGAU-3′, and GSMDA siRNA3: 5′-CAGGAGAGGAGAAGGUCAUCCUUAU-3′) and a negative control siRNA (5′-GAGUGGGUCUGGGUCUUCCCGUAGA-3′) were used. In separate experiments three Stealth™ siRNAs specific for IL37 (RNA duplex sense sequences: IL37 siRNA1: 5′-GAAACUGAUGAAGCUGGCUGCCCAA-3′, IL37 siRNA2: 5′-AGGAAUCAGCACGCCGGCCCUUCAU-3′, and IL37 siRNA3: 5′-UCAUUUCAACCAGUUUGCAAAGCUG-3′) and a negative control siRNA (5′-CCGAUGUGAUGGGCCCAUCUAAUUA-3′) were used. In each knockdown experiment only one siRNA, not a pool of several siRNAs, was transfected into keratinocytes. Untreated, mock-treated, or siRNA-transfected keratinocytes were seeded onto a collagen type I gel containing fibroblasts in cell culture inserts to facilitate the formation of skin equivalents^61^. Keratinocytes were allowed to differentiate for 7 days at the air-liquid interface under supplementation of serum-free KGM2 (Lonza) without bovine pituitary extract and epinephrine, but with 1.5 mM calcium (Sigma), 50 μg/ml ascorbic acid (Sigma) and 0.1% bovine serum albumin (Sigma).

### Reverse transcription (RT) and quantitative polymerase chain reaction (PCR)

RNA was purified from cultured human keratinocytes and from the epidermal compartment of skin equivalents. The latter was peeled off from the dermal compartment using forceps. These samples were homogenized with the Precellys system (VWR International, Radnor, PA) and solubilized with TriFast (VWR International) according to the manufacturer´s instructions. RNA was reverse-transcribed to cDNA with the Iscript^TM^ Kit (Biorad, Hercules, CA). The qPCR was performed using the LightCycler® technology (LC480) and the LightCycler 480 DNA SYBR Green I Master Kit (Roche Applied Science) according to the manufacturer’s protocol. The sequences of the qPCR primers are listed in Supplementary Table S3. Fold-changes relative to the mean of the expression level in preconfluent keratinocytes are shown.

For semi-quantitative PCR of IL37 splice variants and GAPDH (housekeeping gene), the following primers were used: GAPDH-f (5′-accacagtccatgccatcac-3′) and GAPDH-r (5′-tccaccaccctgttgctgta-3′) for GAPDH; IL37-f1 (5′-tgaaccccagtgctgcttaga-3′, annealing in exon 1) and IL37-r1 (5′-ctgagctcaaggatgaggcta-3′, annealing in exon 5) for IL37 mRNA variants v1-v4 (Accession numbers of mRNAs: NM_014439.3; NM_173202.1; NM_173203.1; NM_173204.1); and IL37-f2 (5′-atgtcaggctgtgataggagg-3′) and IL37-r2 (5′-tcctaatcgctgacctcactg-3′) for IL37v5 (Accession number: NM_173205.1). PCRs involved an annealing temperature of 62 °C and 32 cycles for GAPDH and an annealing temperature of 60 °C and 38 cycles for IL37. Three µl of the PCR products were run on a 1.5% agarose gel containing GelRed^TM^ (Biotium) nucleic acid stain.

### Immunohistochemistry

Immunohistochemistry was performed according to published protocols^62^ with modifications. In brief, sections of tissues were fixed with 7.5% formaldehyde and embedded in paraffin. Antigens were demasked with citrate buffer and the samples were incubated with rabbit anti-IL-37 antibody (1:100, HPA054371, lot R72671, Sigma-Aldrich) or with affinity isolated polyclonal rabbit anti-GSDMA (1:200, HPA023313, lot R09704, Sigma-Aldrich). Goat anti-rabbit immunoglobulin (1:100, BA-1000, lot Y1228; Vector Laboratories Inc. Burlingame, CA) was used as secondary antibody. Goat serum (DAKO) (10%) was added to the secondary antibody to suppress unspecific binding. In negative control experiments, the primary antibodies were replaced by anti-rabbit IgG (1 µg/ml) (P120-101, lot P120-101-11, Bethyl). Nuclei were counterstained with hematoxylin.

### Comparative genomics

The genome sequences of bottlenose dolphin (*Tursiops truncatus*), orca (*Orcinus orca*), Yangtze river dolphin (*Lipotes vexillifer*), sperm whale (*Physeter catodon*), minke whale (*Balaenoptera acutorostrata scammoni*), cattle (*Bos taurus*), and humans (*Homo sapiens*) were investigated for the presence and sequence integrity of genes related to pyroptosis and IL-1 signaling. In addition, the genome sequences of other species were used for sequence comparisons. Because of good sequence assembly quality in pyroptosis-related genes, armadillo (*Dasypus novemcinctus*) and elephant shrew (*Elephantulus edwardii*), were chosen as representatives of Atlantogenata, which served as the outgroup to Boreoeutheria (including human, cattle, and cetaceans). The sequences were retrieved from the GenBank database of the National Center for Biotechnology Information (NCBI), USA (http://www.ncbi.nlm.nih.gov/). Gene predictions in the GenBank were used when exon-intron organisation and sequence similarities were compatible with orthology to human genes, otherwise the coding sequences were newly predicted based on these criteria^25^. The Basic Local Alignment Search Tool (BLAST) was used to search for regions of local similarity between sequences. The conservation of blocks of order of genetic elements (synteny) was tested by manual alignment of gene maps including conserved genes on each side the gene(s) of interest. Nucleotide and amino acid sequences were aligned using Multalin with the default parameters for sequence alignments (http://multalin.toulouse.inra.fr/multalin/)^63^.

### Data availability statement

The datasets generated and analysed during the current study are available from the corresponding author on reasonable request.

## Electronic supplementary material

Supplementary Information
