# Supplementary material for: Epidermal cornification is preceded by the expression of a keratinocyte-specific set of pyroptosis-related genes
Source: Sci Rep. 2017 Dec 12;7:17446. doi: 10.1038/s41598-017-17782-4 (PMC5727156; doi:10.1038/s41598-017-17782-4)
Supplement: Supplementary file 1 — Supplementary Information [file 41598_2017_17782_MOESM1_ESM.pdf]

Supplementary Information

Epidermal cornification is preceded by the expression of a keratinocyte-specific set of pyroptosis-related genes

Julia Lachner, Veronika Mlitz, Erwin Tschachler, Leopold Eckhart

Content

Supplementary Figures S1-S16

Supplementary Tables S1-S3

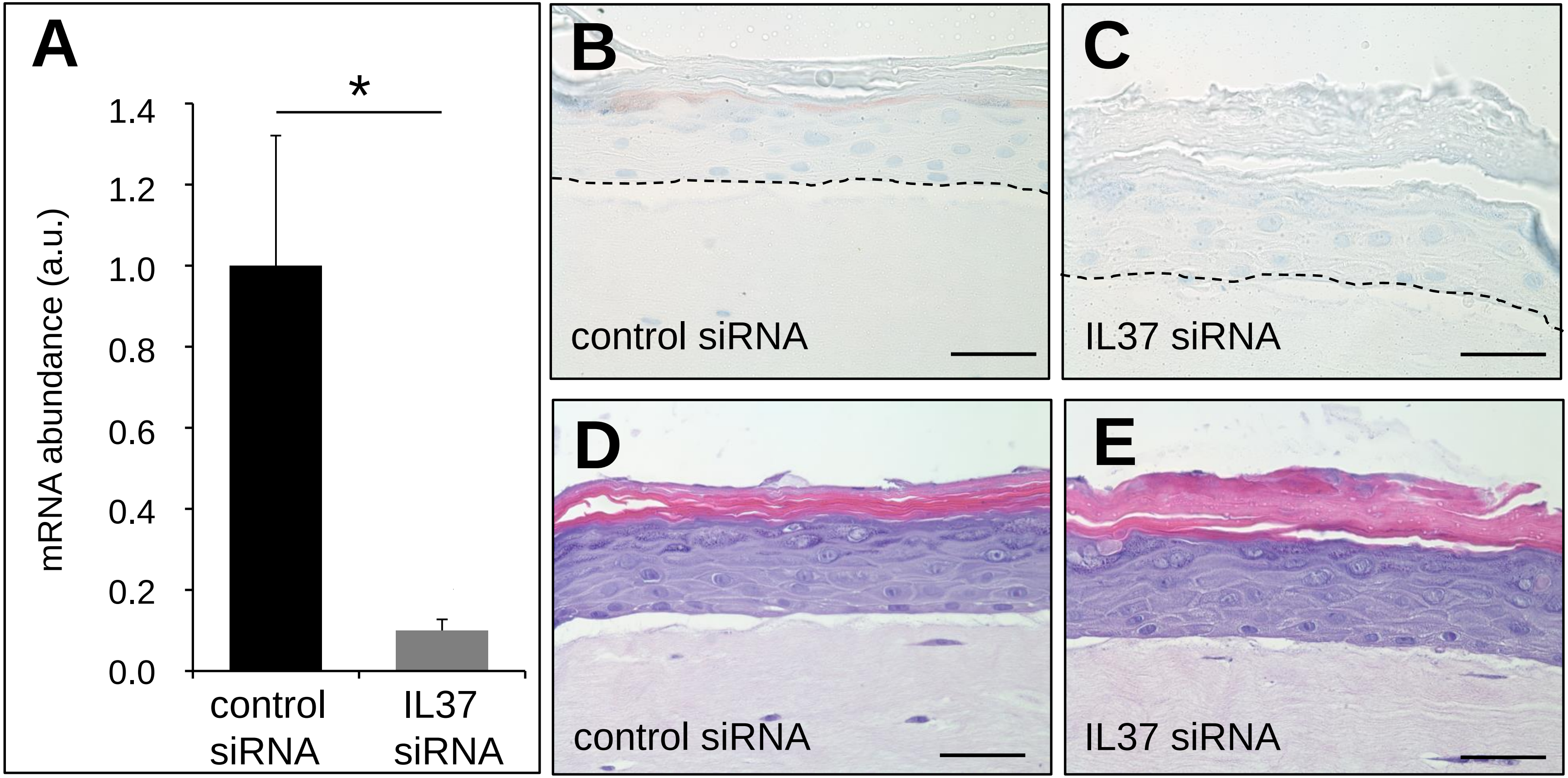

Suppl. Fig. S1. Knockdown of *IL37* confirms specificity of anti-IL-37 immunohistochemistry in human skin equivalents. (**A**) Quantitative RT-PCR analysis of *IL37* expression in keratinocytes treated with control siRNA or siRNA specific for *IL37*. The *IL37* mRNA abundance was normalized to that of the house-keeping gene *B2M*. Bars show the mean (n=3) and error bars show standard deviations. *, $p<0.05$ (t-test). a.u., arbitrary units. (**B, C**) Immunohistochemical detection of IL-37 (red) in skin equivalents comprising keratinocytes treated with control siRNA or *IL37*-specific siRNA. The epidermal-dermal junction is indicated by a broken line. (**D, E**) Hematoxylin and eosin (H&E) staining of skin equivalents comprising keratinocytes treated with control siRNA or *IL37*-specific siRNA. Scale bars, 50 µm. The results shown here (IL37-specific siRNA1) are representative for knockdown experiments with three different IL37 siRNAs, each performed in triplicates.

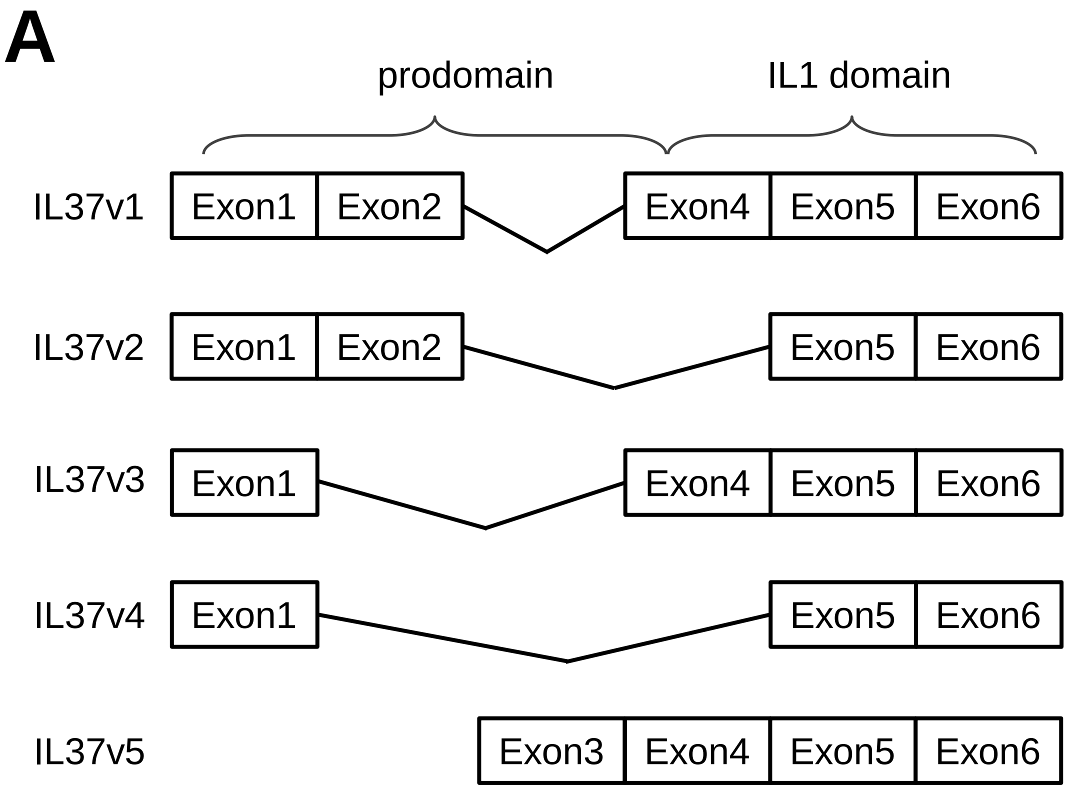

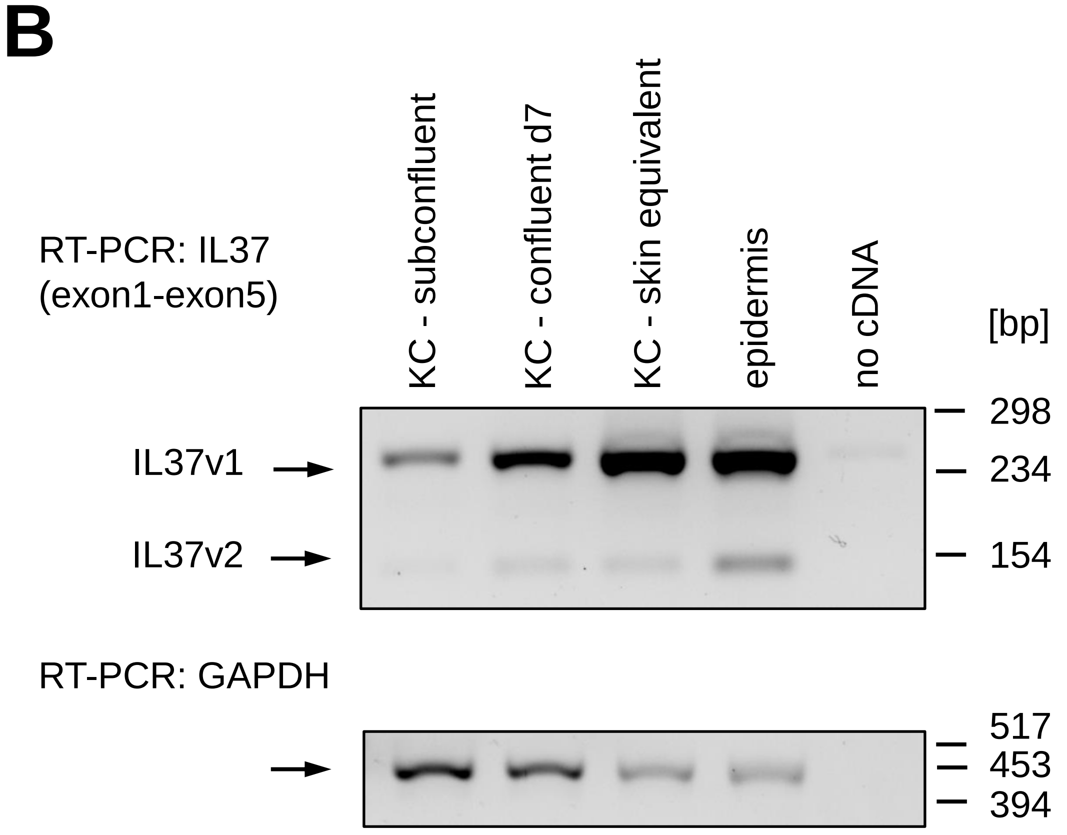

Suppl. Fig. S2. IL37 variant 1 is the predominant mRNA transcribed from the *IL37* gene in epidermal keratinocytes. (**A**) Schematic depiction of alternatively spliced mRNA variants (v1-v5) of human *IL37*. Note that IL37v1 through v4 correspond to variants *b* through *e*, and v5 corresponds to variant *a* in another nomenclature system. Boxes represent mRNA segments (not drawn to scale) that are transcribed from the indicated exons of the *IL37* gene. The encoded protein domains are indicated above. (**B**) RT-PCR analysis of IL37 mRNA variants in cultured keratinocytes (KC) and epidermis using primers annealing to exons 1 and 5 (see Materials and Methods). The predicted PCR product lengths of 242 bp (IL37v1) and 127 bp (IL37v2) matched to the indicated bands. The identity of the bands was confirmed by DNA sequencing. RT-PCR with primers annealing to exons 3 and 5 (specific for v5) did not yield products. bp, base pairs; d7, day 7.

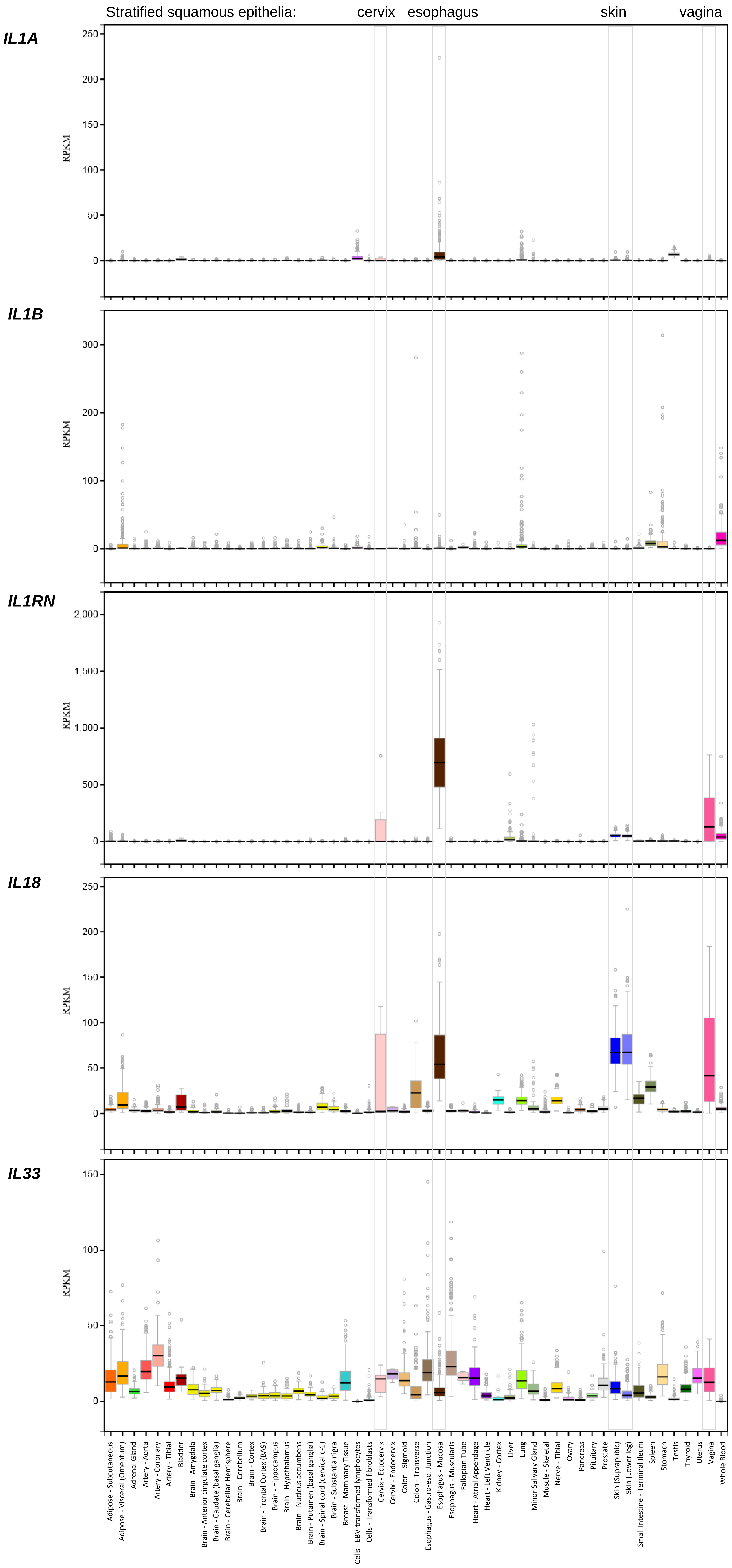

Suppl. Fig. S3. Expression levels of *IL1A*, *IL1B*, *IL1RN*, *IL18*, and *IL33* in human organs, tissues, and cell types. The expression values are shown in RPKM (reads per kilobase of transcript per million mapped reads), calculated from a gene model with isoforms collapsed to a single gene. Box plots show the median and the 25th and 75th percentiles. Outliers are indicated. Organs with stratified squamous epithelia (cervix, esophagus, skin, vagina) are marked by grey boxes. Tissue sampling sites and tissue harvesting methods are described at: https://www.gtexportal.org/home/anatomogramPage. The data used for the analyses described in this manuscript were obtained from: GTEx Analysis Release V6p (dbGaP Accession phs000424.v6.p1) the GTEx Portal (https://www.gtexportal.org/) on 06/26/2017. These data are available without restrictions on use or publication. The Genotype-Tissue Expression (GTEx) Project was supported by the Common Fund of the Office of the Director of the National Institutes of Health, and by NCI, NHGRI, NHLBI, NIDA, NIMH, and NINDS.

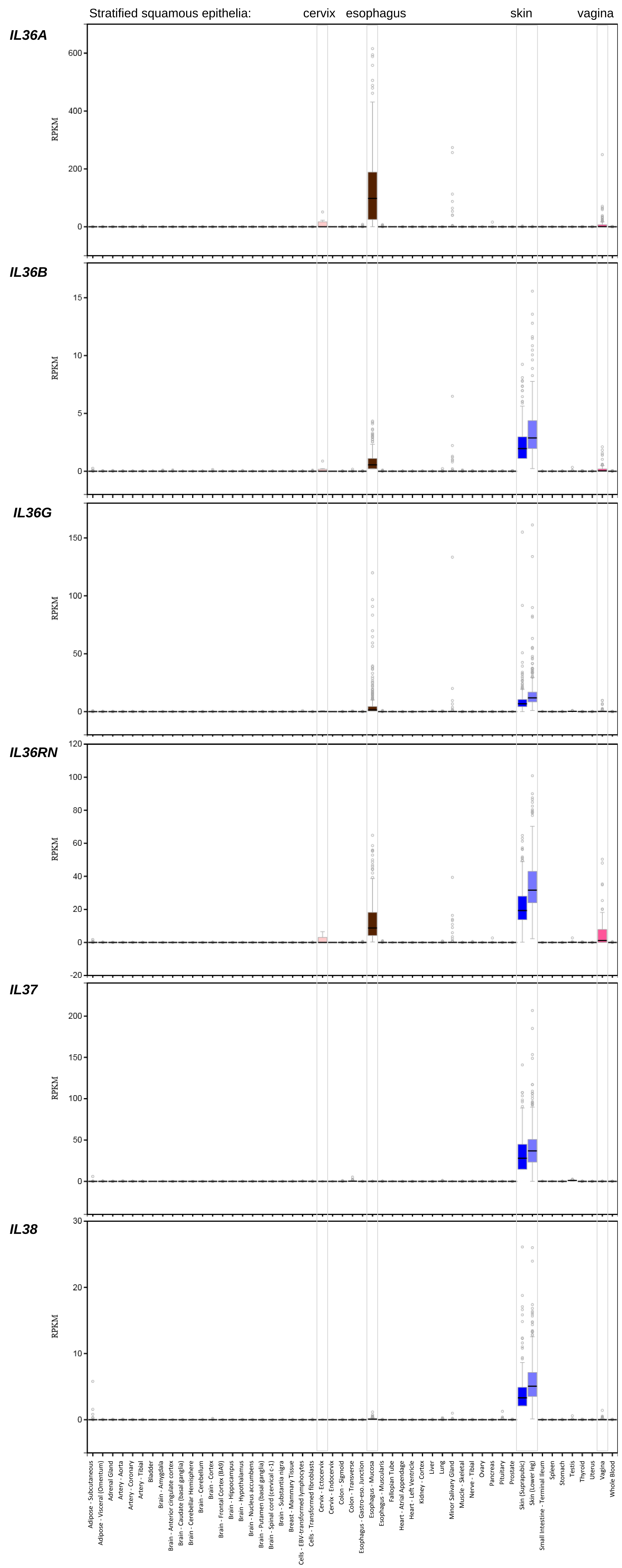

Suppl. Fig. S4. Expression levels of *IL36A*, *IL36B*, *IL36G*, *IL36RN*, *IL37*, and *IL38 (IL1F10)* in human organs, tissues, and cell types. The expression values are shown in RPKM (reads per kilobase of transcript per million mapped reads), calculated from a gene model with isoforms collapsed to a single gene. Box plots show the median and the 25th and 75th percentiles. Outliers are indicated. Organs with stratified squamous epithelia (cervix, esophagus, skin, vagina) are marked by grey boxes. Tissue sampling sites and tissue harvesting methods are described at: https://www.gtexportal.org/home/anatomogramPage. The data used for the analyses described in this manuscript were obtained from: GTEx Analysis Release V6p (dbGaP Accession phs000424.v6.p1) the GTEx Portal (https://www.gtexportal.org/) on 06/26/2017. These data are available without restrictions on use or publication. The Genotype-Tissue Expression (GTEx) Project was supported by the Common Fund of the Office of the Director of the National Institutes of Health, and by NCI, NHGRI, NHLBI, NIDA, NIMH, and NINDS.

A

```
        1                                                          60
Hs_IL1A MAKVPDMFEDLKNCYSENEEDSSSIDHLSLNQKSFYHVSYGPLHEGCMDQSVSLSISETS
Bt_IL1A MAKVPDLFEDLKNCYSENEDYSSEIDHLSLNQKSFYDASYEPLREDQMNKFMSLDTSETS
Tt_IL1A MAKVPDLFEDLKNCYSENEEYSSEIDHLSLNQKSFYDASYEPLHQDCIDKYMSLNTPETS
Oo_IL1A MAKVPDLFEDLKNCYSENEAYSSEIDHLSLNQKSFYDTSYEPLRQDCIDKYMSLNTPETS
Lv_IL1A MAKVPDLFEDLKNCYSENEEYSSEIDHLSLNQKSFYDASYEPLHEDCMDKFMSLNTPETS
Pc_IL1A MAEVPDLFEDLKNCYSENEEYSSEIDHLSLNQKSFYDASYEPLHEDCMDKFMSLNSSETS
Ba_IL1A MAKVPDLFEDLKNCYSENEEYSSEIDHLSLNQKSFYDASYEPLHEDCMDKFISLNTSETS

        61                                                        120
Hs_IL1A KTSKLTFKESMVVVATNGKVLKKRRLSLSQSITDDDLEAIANDSEEEIIKPRSAPFSFLS
Bt_IL1A KTSKLSFKENVVMVAASGKILKKRRLSLNQFITDDDLEAIANNTEEEIIKPRSAHYSFQS
Tt_IL1A KTSKLTFKENVMMVAANGKILKNRWLSLNQFLTDDDLEDIANDTEEEIIKPRSAHYSFQS
Oo_IL1A KTSKLTFKENVMMVAANGKILKNRWLSLNQFLTDDDLEDIADDTEEEIIKPRSAHYSFQS
Lv_IL1A KTSKLTFKENVVMMAAKGKILKNRWLSLNQFLTDDDLEDMANDTEEEIIKPRSAHYSFQS
Pc_IL1A KTSKLTFKENVGMVAANGKILKKRWLSLNQFITNDDLEDIANDTEEXXXXXXXXXXXXXX
Ba_IL1A KTSKLTFKENVAKVAANGKILKKRRLSLNQFITDDDLKDITNDTEEEIIKPRSAHYSFQS

        121                                                       180
Hs_IL1A NVKYNFMRIIKYEFILNDALNQSIIR-ANDQYLTAAALHNLDEAVKFDMGAYKSSKDDAK
Bt_IL1A NVKYNFMRVIHQECILNDALNQSIIRDMSGPYLTATTLNNLEEAVKFDMVAY-VSEEDSQ
Tt_IL1A NMKYNFIKLIQQQYILNDALHQSIVLHPSGQHLMAAALNNLDEAVEFDIAAY-TSGDDTQ
Oo_IL1A NMKYNFIKLIQQQYILNDALHQSIVLHPSGQYLMAAALNNLDEAVEFDIAAY-TSGDDTQ
Lv_IL1A NMQYNFIRVIKEQCILNDALHQSIVQDSSGPYLVAAALNNLDEAVKFDVSSY-TSEEDSL
Pc_IL1A XXXXXXXXXXXXXXXXXXXXXXXXXXXXXXXXXXXXXXXXXXXXVKFDMCAY-TSEEDSQ
Ba_IL1A NMKYNFMGVIKQQCILNDALHQSIVQDSSGQYLMAAALNNPDEAVKFDMCAY-IPEEDLQ

        181                                                       240
Hs_IL1A ITVILRISKTQLYVTAQDEDQPVLLKEMPEIPKTITGSETNLLFFWETHGTKNYFTSVAH
Bt_IL1A LPVTLRISKTQLFVSAQNEDEPVLLKEMPETPKIIK-DETNLLFFWEKHGSMDYFKSVAH
Tt_IL1A LPVTLRISKTQLFVCAQNEGEPVLLKEMPETPQTIK-DETDLLFLWSNHGSMQHFKSVAH
Oo_IL1A LPVTLRISKTQLFVCAQNEGEPVLLKEMPETPQTIK-DETDLLFLWSNHGSMQHFKSVAH
Lv_IL1A LPVTLRISKTQLFVCAQNEDEPVLLKEMPETPQTIK-DETNLLFFLERTGSMSNFKSVAH
Pc_IL1A LPVTLRISNTRLFVCAQNEDEPVLLKEMPETPKTIK-DETNLLFFWEKHGTIHYFKSVAH
Ba_IL1A CPVTLRISKTLLFVCAQNEDEPVLLKEMPEIPKTIK-DETNLLFFWEEHGTMCYFKSVAN

        241                              272
Hs_IL1A PNLFIATKQDYWVCLAGGPPSITDFQILENQA
Bt_IL1A PKLFIATKQEKLVHMASGPPSITDFQILEK--
Tt_IL1A PKLYFATKGEKLVHLARGPPSIIDFQL-----
Oo_IL1A PKLYFATKGEKLVHLARGPPSIIDFQL-----
Lv_IL1A PKLYFATKQEKRVHLARGPPSNTDFQF-----
Pc_IL1A PKLYFATKQEKLVHLARGLPSITDFQILENQL
Ba_IL1A SKLYLATKQEKLVHLARGPPSITDFQMSENQP
```

Suppl. Fig. S5. Amino acid sequence alignment of interleukin-1 family (IL1F) proteins. Continued on the next page.

B

```
         1                                                          60
Hs_IL1B  MAEVPELASEMMAYYSGNEDDLFFEADGPKQMKCSFQDLDLCPL-DGGIQLRISDHHYSK
Bt_IL1B  MATVPEPINEMMAYYS-DENELLFEADDPKQMKSCIQHLDLGSMGDGNIQLQISHQFYNK
Tt_IL1B  MATVPEPTNEVMAYYS-DENDLLFEADGPKQMKCCVQHLDLSSTGDESIHLQISHQLYNK
Oo_IL1B  MATVPEPTNEVMAYYS-DENDLLFEADGPKQMKCCVQHLDLSSTGDESIHLQISHQLYNK
Lv_IL1B  MATVPEPTNEVMDYYS-DENDLLFEADSPKQMKCCVQHLDLSSTGDGSIHLQISHQLYNK
Pc_IL1B  MAMVPEPANEVMAYYS-DENDLLFEADGPKQMKCCVQHLDLSSTGDGSIQLQICHQLHDK
Ba_IL1B  MATVPEPTNEVMAYYS-DENDLLFEADGPKQMKCCVQHLDLSSVGDGSIQLQISHQLYNK

         61                                                        120
Hs_IL1B  GFRQAASVVVAMDKLRKMLVPCPQTFQENDLSTFFPFIFEEEPIFFDTWDNEAYVHDAPV
Bt_IL1B  SFRQVVSVIVAMEKLRN--SAYAHVFHDDDLRSILSFIFEEEPVIFETSSDE-FLCDAPV
Tt_IL1B  SFRHVVSVIVAVEKLQK--IPCSQTFQDDGLRSIFSLIFEEEPVIFETYDDD-LLCDAAV
Oo_IL1B  SFRHVVSVIVAVEKLQK--IPCSQTFQDDGLKSIFSLIFEEEPVIFETYDDD-LLCDAAV
Lv_IL1B  SFRKVVSVIVAMEKLQK--VPCSQTFQDDGLRSILSLIFQEEPVIFETYDDD-LLCDAVV
Pc_IL1B  SFRQVVSVIVAMEKLKR--VPCSQAFQDDDLRGIFSVIFEEEPVIFETYTDD-LLCDAGV
Ba_IL1B  SFRQVVSVIVAMEKLKR--IPCSQAFQDDDLRSIFSLIFEEEPVIFETYADD-FLCDAGV

         121                                                       180
Hs_IL1B  RSLNCTLRDSQQKSLVMSGPYELKALHLQGQDMEQQVVFSMSFVQGEESNDKIPVALGLK
Bt_IL1B  QSIKCKLQDREQKSLVLASPCVLKALHLLSQEMNREVVFCMSFVQGEERDNKIPVALGIK
Tt_IL1B  QSLTCKLQDRDQKSLVLASPCVLKALHLLARDMNREVVFCMSFVQGDESNDKIPVALGLK
Oo_IL1B  QSLTCKLQDRDQKSLVLASPCVLKALHLLARDMNREVVFCMSFVQGDESNDKIPVALGLK
Lv_IL1B  QSLNCKLQDRDQKSLVLASPCVLKALHLLERDVNREVVFCMSFVQGDESNDKIPVALGLK
Pc_IL1B  HSLTCKLQDKEQKSLVLASPYVLKALHLLARDVNREVVFCMSFVQGDESDDKIPVALGLK
Ba_IL1B  QSLNCKLQDKEQKSLVLAGPCVLKALHLLARDMNREVVFCMSFVQGDEREDKIPVALGLK

         181                                                       240
Hs_IL1B  EKNLYLSCVLKDDKPTLQLESVDPKNYPKKKMEKRFVFNKIEINNKLEFESAQFPNWYIS
Bt_IL1B  DKNLYLSCVKKGDTPTLQLEEVDPKVYPKRNMEKRFVFYKTEIKNTVEFESVLYPNWYIS
Tt_IL1B  EKNLYLSCVMKGDRPILQLEEVDPKTYPKWKMEKRFVFNKTEIKNSVEFESALYPNWYIS
Oo_IL1B  EKNLYLSCVMKGDRPILQLEEVDPKTYPKWKMEKRFVFNKTEIKNSVEFESALYPNWYIS
Lv_IL1B  EKNLYLSCVMKGDRPTLQLEEVDPKTYPKWKMEKRFVFNKTEIKNSVEFESALYPNWYIS
Pc_IL1B  EKNLYLSCVMKGDRPTLQLEEVDPKTYPKWKMEKRFVFNKTEIKNRVEFESALYPNWYIS
Ba_IL1B  EKNLYLSCVMKGDRPTLQLEEVDPKTYPKWNMEKRFVFNKTEVKNTVEFESALYPNWYIS

         241                           270
Hs_IL1B  TSQAENMPVFLGGTKGGQDITDFTMQFVSS
Bt_IL1B  TSQIEERPVFLGHFRGGQDITDFRMETLSP
Tt_IL1B  TSQAEEKPIFLGRSKGGHDITDFTMEIISP
Oo_IL1B  TSQAEEKPIFLGRSKGGHDITDFTMEIISP
Lv_IL1B  TSQAEEKPIFLGRSKGGHDITDFTMEIISP
Pc_IL1B  TSQAEEKPVFLGRSKGGHDITDFTMEIISP
Ba_IL1B  TSQAEEKPIFLGRSKGGHDITDFTMEIISP
```

Suppl. Fig. S5. Amino acid sequence alignment of interleukin-1 family (IL1F) proteins. Continued on the next page.

C

```
               1                                                          60
Hs_IL1RN  MEICRGLRSHLITLLLLFLFHSETICRPSGRKSSKMQAFRIWDVNQKTFYLRNNQLVAGYL
Bt_IL1RN  MDIY--IHGYLICLLLFLFRSETACHPLGKRRCEMQAFRIWDVNQKIFYLRNNQLVAGYL
Tt_IL1RN  MEVCRCHHGYLISLLLFLFHSETACYPLGKRPCEMQAFRIWDVNQKTFYLRNNQLVAGYL
Oo_IL1RN  MEVCRCHHGYLISLLLFLFHSETACYPLGKRPCEMQAFRIWDVNQKTFYLRNNQLVAGYL
Lv_IL1RN  MEVCRCHHGYLISLLLFLFHSETACHPLGKRPCEMQAFRIWDVNQKTFYLRNNQLVAGYL
Pc_IL1RN  MEVGRCHHGYLISLLLFLFHSETACYPLGNRPCEMQTFRIWDVAQKTFYLRNNQLVAGYL
Ba_IL1RN  MEVCRCHHGYLISLLLFLFHSETACYPLGKRPCEMQAFRIWDVTQKTFYLRNNQLVAGYL

          61                                                         120
Hs_IL1RN  QGPNVNLEEKIDVVPIEPHALFLGIHGGKMCLSCVKSGDETRLQLEAVNITDLSENRKQD
Bt_IL1RN  QGPNTKLEEKIDVVPIEPHTMFLGIHGGKLCLACVKSGDEIKLKLEAVNITDLNQNREQD
Tt_IL1RN  QGPNTKLEEKIDVVPIEPHAMFLGIHGGKLCLACVKSGDEIKLGLEPVNITDLNSSKEED
Oo_IL1RN  QGPNTKLEEKIDVVPIEPHAMFLGIHGGKLCLACVKSGDEIKLGLEPVNITDLNSSKEED
Lv_IL1RN  QGPNTKLEEKIDVVPIEPHAMFLGIHEGKLCLACVKSGNEIKLGLEPVNITDLNSSKEED
Pc_IL1RN  QGANTKLEEKIDVVPVEPHTMFMGIHGGKLCLACVKSGNEIKLGLEPVNITDLSTSKEED
Ba_IL1RN  QGPNTKLEEKIDVVPIEPHAMFLGIHGGKLCLACVKSGNEIKLGLEPVNITDLSRNNEED

          121                                                  177
Hs_IL1RN  KRFAFIRSDSGPTTSFESAACPGWFLCTAMEADQPVSLTNMPDEGVMVTKFYFQEDE
Bt_IL1RN  KRFAFIRFDNGPTTSFESAACPGWFLCTSLEADQPVGLTNMPTEALKVTKFYFQQD-
Tt_IL1RN  KRFAFIRSDSGPTTSFESAACPGWFLCTALETDQPVGLTNTPQDAVQVTKFYFQQDQ
Oo_IL1RN  KRFAFIRSDNGPTTSFESAACPGWFLCTALETDQPVGLTNTPQDAVQVTKFYFQQDQ
Lv_IL1RN  KRFAFIRFDSGPTTSFESAACPGWFLCTALETDQPVGLTNTPKDAVQVTKFYFQQDQ
Pc_IL1RN  KRFAFIRSDSGPTTSFESAACPGWFLCTATETDQPVGLTNKPQDAVQVTKFYFQQDQ
Ba_IL1RN  KRFAFIRSDSGPTTSFESAACPGWFLCTALETDQPVGLTNAPQDAVQVTKFYFQQDQ
```

Suppl. Fig. S5. Amino acid sequence alignment of interleukin-1 family (IL1F) proteins. Continued on the next page.

D

```
        1                                                          60
Hs_IL18 MAAEPVEDNCINFVAMKFIDNTLYFIAEDDENLESDYFGKLESKLSVIRNLNDQVLFIDQ
Bt_IL18 MAAEQVEDYCISFVEMKFINNTLYFVAENDEDLESDHFGKLEPKLSIIRNLNDQVLFINQ
Tt_IL18 MAAEPVEDNCISFVEMKFINNTLYFVAESDEDLESDYFGKLEPKFSVIRNLNDQVLFINQ
Oo_IL18 MAAEPVEDNCISFVEMKFINNTLYFVAESDEDLESDYFGKLEPKFSVIRNLNDQVLFINQ
Lv_IL18 MAAELVEDNCISFVEMKFINNTLYFVAENDEDLESDYFGKLEPKFSVIRNLNDQVLLINK
Pc_IL18 MAAEPVEDNCISFVEMKFINNTLYFVAENDEDLESDYFGKLEPKFSVIRNLNDQVLFINQ
Ba_IL18 XXXXXXXXXXXXXXXXXXINSTLYFVAENDEDLESDYFGKLEPKFSVIRNLNDQVLFINQ

        61                                                        120
Hs_IL18 GNRPLFEDMTDSDCRDNAPRTIFIISMYKDSQPRGMAVTISVKCEKISTLSCENKIISFK
Bt_IL18 GNQPVFEDMPDSDCSDNAPQTIFIIYMYKDSLTRGLAVTISVQCKKMSTLSCENKIVSFK
Tt_IL18 ESQPVFEDMPDSDCSDNAPQTIFVICMYKDSLTRGLAVTISVHCKKIFTLSCKNKILSFK
Oo_IL18 ESQPVFEDMPDSDCSDNAPQTIFIICMYKDSLTRGLAVTISVHCKKIFTLSCKNKILSFK
Lv_IL18 ESQPVFEDMPDSDCSDNAPQTIFIICMYKDSLTRGLAVTISVHCKKISTLSCKNKILSFK
Pc_IL18 GSQPVFEDMPDSDCSDNAPQTIFIICMYKDSLTRGLAVTISVHFKKISTLSCKNKILSFK
Ba_IL18 GSQPVFEDMPDSDCSDNAPQTIFIICMYKDSLTRGLAVTISVHCKKISTLSCKNKIISFK

        121                                                       180
Hs_IL18 EMNPPDNIKDTKSDIIFFQRSVPGHDNKMQFESSSYEGYFLACEKERDLFKLILKKEDEL
Bt_IL18 EMNPPDNIDNEESDIIFFQRSVPGHDDKIQFESSLYKGYFLACKKENDLFKLILKKQDDN
Tt_IL18 EMSPPDNIDDEGNDIIFFQRSVPGHDDKIQFESSLYKGYFLACKKENDLFKLILKKKDEN
Oo_IL18 EMSPPDNIDDEGNDIIFFQRSVPGHDDKIQFESSLYKGYFLACKKENDLFKLILKNKDEN
Lv_IL18 EMSPPDNIDDEGNDIIFFQRSVPGHDDKIQFESSLYKGYFLACKKENDLFKLILKKKDEN
Pc_IL18 EMSPPDNIDDEGNDIIFFQRSVPGHDDKIQFESSLYKGYFLACKKENDIFKLILKEKDEN
Ba_IL18 EMSPPDNIDDEGNDIIFFQRSVPGHDDKIQFESSLYKGYFLACKKENDLFKLILKKKDEN

        181       193
Hs_IL18 GDRSIMFTVQNED
Bt_IL18 RDKSVMFTVQNQN
Tt_IL18 GDKSVMFTVQNKN
Oo_IL18 GDKSVMFTVQNKN
Lv_IL18 GDKSVMFTVQNKN
Pc_IL18 GDKSVMFTVQNKN
Ba_IL18 GDKSVMFTVQNKN
```

Suppl. Fig. S5. Amino acid sequence alignment of interleukin-1 family (IL1F) proteins. Continued on the next page.

E

```
          1                                                          60
Hs_IL33   MKPKMKYSTNKISTAKWKNTASKAL--CFKLGKSQQKAKEVCPMYFMKLRSGLMIKKEAC
Bt_IL33   MKPKMKYSTTKISPAKMKCSAGKALVKSPKLRKSQQKAEEVCQIYYMQLRSGLKIEKKVC
Tt_IL33   MKPEMKYSTTKISPAKMNSSAGKAL--SYSLGKSQQKAEEVCPMVFMQLRSGLKIEKKTC
Oo_IL33   MKPEMKYSTTKISPAKMNSSAGKALVKSPKLRKSQQEAEEVCPMVFMQLRSGLKIEKKTC
Pc_IL33   MKPKMKYSTIKISPAKMNSSAGKALVKSPKLRKSQQKAEEICPMYFMQLRSGLKIEKKTC
Ba_IL33   MKPKMKYSTTKISPAKMHSSAGKALAKSPKLRKSQQKAEEVCPMYFMQLRSGLKIEKKTC

          61                                                        120
Hs_IL33   YFRRETTKRPSLKTGRKHKR-HLVLAACQQQSTVECFAFGISGVQKYTRALHDSSITGIS
Bt_IL33   YFRKETTKRHSL-TAEKYKE-HLALVACEQLNHLQQSVEQGFTLGK-TMVP-YTTATGLP
Tt_IL33   YFRKETTKRHSPRTAKMYKEKHLVFAACQELEHLGPSVKS-FAFDK-TRVQKYTTATGLP
Oo_IL33   YFRKETTKRHSPRTAKTYKEKHLVFAACQELEHLGPSVKS-FAFDK-TRVQKYTTATGLP
Pc_IL33   YFRKETTKRYSPRTAKKYKEQHLVFAACQELEHLGQSVKS-FAFDK-TRVQKYTTATGLP
Ba_IL33   YFRKETTKRHSLRTAKKYKEQRLVFAACQELEHIGQSVKS-FTFDK-TRVQKYTTATGLP

          121                                                       180
Hs_IL33   PITEYLASLSTYNDQSITFALEDESYEIYVEDLKKDEKKDKVLLSYYESQHPSNESGDGV
Bt_IL33   SIKEHSASLSTYNDQFITFVLEDGSYEIYVEDLIDNQEKDKVLLRYYDSQFPSSETDDGG
Tt_IL33   SIKEHSASLSTYNDQYITFVFEDGNYEIYVEDLGKDQEKDKVLLRYYDSQFPSSETEGGG
Oo_IL33   SIKEHSASLSTYNDQYITFVFEDGNYEIYVEDLGKDQEKDKVLLRYYDSQFPSSETEGGG
Pc_IL33   SIKEHSASLSTYKDQSITFVFEDGNYEIYVDDLGKDQEKDKVLLRYYDSQFPSSETDGGG
Ba_IL33   SIKEHSASLSTYNDQSITFVFEDGNYEIYVDDLGKDQEKDKVLLRYYDSQFPSSETDGGG

          181                                                       240
Hs_IL33   DGKMLMVTLSPTKD--FWLHANNKEHSVELHKCEKPLPDQAFFVLHNMHSNCVSFECKTD
Bt_IL33   SHRKLMVNLSPTKDKDFLLHANSKEHSVELQKCENQLPEQTFFVLHETSSQCVSFECKSN
Tt_IL33   DHRKLMVNLSPTKDKDFLLHANSKEHSVELQKCENQLPEQAFFVLHEETSQCVSFECKSN
Oo_IL33   DHRKLMVNLSPTKDKDFLLHANSKEHSVELQKCENQLPEQAFFVLHEETSQCVSFECKSN
Pc_IL33   DHRKLMVNLSPTKDKDFLLHANSKEHSVELQKCENQLPEQAFFVLHEETSQCVSFECKSN
Ba_IL33   DHRKLMVNLSPTKDKDFLLHANSKEHSVELQKCENPLPEQAFFVLHEETSQCVSFECKSN

          241                                 277
Hs_IL33   PGVFIGVKDNHLALIKV-DSSENLCTENILFKLSET-
Bt_IL33   PGVFLGVKDNQLALIKRGEHPEDSNSQNITFKLSNLM
Tt_IL33   PGVFLGVKDNHLALIKRGEHPEDSNEENTIFKLSNLI
Oo_IL33   PGVFLGVKDNHLALIKRGEHPEDSNEENTIFKLSNLI
Pc_IL33   PGVFLGVKDNHLALIKRGEHPEDSNEENIIFKLSNLM
Ba_IL33   PGVFLGVKDNHLALIKRGEHPEDSNEENTIFKLSNLI
```

Suppl. Fig. S5. Amino acid sequence alignment of interleukin-1 family (IL1F) proteins. Continued on the next page.

F

```
              1                                                          60
Hs_IL36A      --------------------MEKALKIDTPQQGSIQDINHRVWVLQDQTLIAVPRKDRMSP
Bt_IL36A      MSSHLGRSETPSSKGDGKVKGPVLSRIHPILVNIQDINHLVWVLQGQTLTAVPRKDQMDP

              61                                                        120
Hs_IL36A      VTIALISCRHVETLEKDRGNPIYLGLNGLNLCLMCAKVGDQPTLQLKEKDIMDLYNQPEP
Bt_IL36A      VTVTLVSCKYTETLEKGRGNPVYLGLKEPELCLFCTKVKGQPTLQLQERNIMDLYHQTEP

              121                                                     178
Hs_IL36A      VKSFLFYHSQSGRNSTFESVAFPGWFIAVSSEGGCPLILTQELGKANTTDFGLTMLF-
Bt_IL36A      VKPFLFYHDQNGRASSFESVAFPGWFIGSCSCGGCPVIITQELGKIYTTDFGFTVLQP
```

G

```
              1                                                          60
Hs_IL36B      MN-PQREAAPKSYAIRDSRQMVWVLSGNSLIAAPLSRSIKPVTLHLIACRDTEFSDKEKG
Bt_IL36B      MECPMLREYPSYLHIRDSRQMVWVVKGNSLIAVPSSNNIKPVILSLIACRDMEFNKEGNG

              61                                                        120
Hs_IL36B      NMVYLGIKGKDLCLFCAEIQGKPTLQLKLQGSQDNIGKDTCWKLVGIHTCINLDVRESCF
Bt_IL36B      TPHYLGIKDKNLCLYCTEIQGYPTLQLKEENIMNLYNKPKGEKCFLFY---RNDEGSTVV

              121                                        165
Hs_IL36B      MGTLDQWGIGVGRKKWKSSFQHHHLRKKDKDFSSMRTNIGMPGRM
Bt_IL36B      FQSVSYPGWFIAT----SSEAGHPVTLTKERGTVQSTNYYLEGGL
```

Suppl. Fig. S5. Amino acid sequence alignment of interleukin-1 family (IL1F) proteins. Continued on the next page.

H

```
          1                                                          60
Hs_IL36G  MRGTPGDADGGGRAVYQSMCKPITGTINDLNQQVWTLQGQNLVAVPRSDSVTPVTVAVIT
Bt_IL36G  MACFLGH-DGG--IHTSAMDCPRYVVVSDLSQQVWFLQGQILVVVPRSNNVRPVTVTIIP
Tt_IL36G  MAEIM-EASGP--VYISKMDEPWTGECFDLNQQAWILKGHTLVTAPLNNSVTPVTVTVMP
Oo_IL36G  MAEIT-EASGP--FYKAEMDEPWTGECFDLNQQAWILKGHTLVTVPLNNSVTPVVVTVMP
Lv_IL36G  MAADL-ETSGP--SHKAEMDKPWIGECFDLNQQVWILQGHTLVTTPWNNGVTPATVTVLP
Pc_IL36G  MADDV-ETRAV--YRTEGLDKPWSGQISDVNQQVWILQGQTLVTAPWNSGVTPATVTVLP
Ba_IL36G* MAGAL-EAHEL--VCTV-MEKPRVGEVFDLNQQVWFLQGQTLVAVPWSNDVTPVTVTVAP

          61                                                        120
Hs_IL36G  CKYPEALEQGRGDPIYLGIQNPEMCLYCEKVGEQPTLQLKEQKIMDLYGQPEPVKPFLFY
Bt_IL36G  CKYPECLEKDKGIPIYLGIKQPEMCLCCEDVGGKPELQLKNQKIMDLYNQAEPVKPFLFY
Tt_IL36G  CKNPGSVEEDRGVPIYLGIENPEMCLYCEDVGGQPKLQLKDQKILDLYNRPEPMEPFLFY
Oo_IL36G  CKNPGSVEEDKGVPIYLGIENPEMCLYCEDVGGQPKLQLKDQKILDLYNRPEPMEPFLFY
Lv_IL36G  CKNPGSVEKDKGIPIYLGIQDPEMCLYCEDVGGQPKLQLKDQKILDLYNQAEPVEPFLFY
Pc_IL36G  FKNPDSLEKEKGIPIYLGIQNLEMCLYCEDAGGQPKLQLKDQNILHLYNQAEPIEPFLFY
Ba_IL36G* CKNPGSLEKDKGIPIYLGIQNPEMWLHCEDVGGQPILQLKTP---DLCNQAKPMKPFLFY

          121                                                  177
Hs_IL36G  RAKTGRTSTLESVAFPDWFIASSKRDQPIILTSELGKSYNTAFELNIND--------
Bt_IL36G  RQSTGSTSTFESVAFPGWFIASSERNQPIFLTSELGNIYNTAFQLDSKEFSPSNNSL
Tt_IL36G  HGRTGSTSTFESVAFPDWFIASSEQGQPIFLTSNLGKMYSTAFRIDLRI--------
Oo_IL36G  HGRTGSTSTFESVAFPDWFIASSEQGQPIFLTSNLGKMYSTAFRIDLGFNSA-----
Lv_IL36G  RDQTGSTSTFESVAFPDWFIASSEKGQPIFLTSDLGKMYNTAFRIDLTQPGGSSLVL
Pc_IL36G  RGQTGSTSTFESVAFPGWFIASSKKGQPIVLTSDLGRMYTTDFRIDLSF--------
Ba_IL36G* HVQTDINSTFESVAFPSWFIASSKRGQPIFLTSDLGRMYSTAFRMNLRI--------
```

I

```
          1                                                          60
Hs_IL36RN MVLSGALCFRMKDSALKVLYLHNNQLLAGGLHAGKVIKGEEISVVPNRWLDASLSPVILG
Bt_IL36RN MVLSGALCFRMKDAALKVLYLHDNQLLAGGLQAGKVIKGEEISVVPNRSLDAKLSPVILG
Pc_IL36RN MVLSGALCFRMKDATLKMLYLHNNQLQAGGLQAGKVIKGEAISVVPNRSLDAKLSPVILG
Tt_IL36RN MVLSGALCFRMKDATLKVLYLQDNQLQAGALQAGKVIKGEEISVVPNRYLDAKLSPVILG
Oo_IL36RN MVLSGALCFRMKDATLKVLYLQDNQLQAGGLQAGKVIKGEEISVVPNRYLDAKLSPVILG
Ba_IL36RN MVLSGALCFRMKDARLKGLYLHDNQLQAGGLQAGKVIKGEEISVVPSQSLDAKLSPVMLG

          61                                                        120
Hs_IL36RN VQGGSQCLSCGVGQEPTLTLEPVNIMELYLGAKESKSFTFYRRDMGLTSSFESAAYPGWF
Bt_IL36RN VHGGSQCLSCGTGQEPTLKLEPVNIMELYHSAEKSKKFTFYRRDTGLTSSFESAAYPGWF
Pc_IL36RN VQGGSQCLSCGTGQEPTLKLEPVNIMELYHSAEESKRFTFYRRETGLTSSFELAAFPGWF
Tt_IL36RN VQGGSQCLSCGMEQEPTLKLEPVNIMELYHSAEESKRFTFYRRDTGLTSSFEVATYPGWF
Oo_IL36RN VQGGSQCLSCGMEQEPTLKLEPVNIMELYHSAEESKRFTFYRRDTGLTSSFEVATYPGWF
Ba_IL36RN VQGGSQCLSCGTGQEPTLKLEPVNIMELYHSAEESKRFTFYWRDTGFTSSFELAAYPGWF

          121                               155
Hs_IL36RN LCTVPEADQPVRLTQLPENGGWNAPITDFYFQQCD
Bt_IL36RN LCTVPEADQPLQITQLPKDTSWDNPIIDFYFQQCD
Pc_IL36RN LCTMPEADQPLRVTQLPKDTSWDGPITDFYFQQCD
Tt_IL36RN LCTMPEVDQPLRVTQLPKDTSLDGPITDFYFQQCD
Oo_IL36RN LCTMPEVDQPLRVTQLPKDTSLDGPITDFYFQQCD
Ba_IL36RN FCTVPEADQPLRVTQLLKDTSWDGPITDFYFQQYD
```

Suppl. Fig. S5. Amino acid sequence alignment of interleukin-1 family (IL1F) proteins. Continued on the next page.

J

```
        1                                                          60
Hs_IL37 MSFVGENSGVKMGSEDWEKDEPQCCLEDPAGSPLEPGPSLPTMNFVHTSPKVKNLNPKKF
Bt_IL37 MSVLEENPGMKMDCEDWERDEPQCSSEDSVRDALEPGPSLTSMSAAHAGPRVKANGPEKF

        61                                                        120
Hs_IL37 SIHDQDHKVLVLDSGNLIAVPDKNYIRPEIFFALASSLSSASAEKGSPILLGVSKGEFCL
Bt_IL37 TIHDGDQKVLVLDSKTLRAVPDKTYILPEIFFVLASRVKSAYENKGSPIFLAVSKGQLCL

        121                                                       180
Hs_IL37 YCDKDKGQSHPSLQLKKEKLMKLAAQKESARRPFIFYRAQVGSWNMLESAAHPGWFICTS
Bt_IL37 CCDTNKGHK-PSLQLKKKKLSKLAAQKKGKYLPFIFYRNKVGSRNTLESAAHPGWFVCTF

        181                                  218
Hs_IL37 CNCNEPVGVTDKFENRKHIEFSFQPVCKAEMSPSEVSD
Bt_IL37 PNPGKPVGMTKSHGRRKHTEFSFRRI------------
```

K

```
        1                                                          60
Hs_IL38 MCSLPMARYYIIKYADQKALYTRDGQLLVGDPVADNCCAEKICILPNRGLARTKVPIFLG
Bt_IL38 MCSLPMAKYYIIKDAEQKALYMRDGQFLVGDPNADNCHAETICILPNRGLERTKFPIFLG

        61                                                        120
Hs_IL38 IQGGSRCLACVETEEGPSLQLEDVNIEELYKGGEEATRFTFFQSSSGSAFRLEAAAWPGW
Bt_IL38 VQGGSRCLACVETGEGPSLQLEDVNIEDLYKGGEEATRFTFFQRSSGPAFRLEAAAWPGW

        121                             152
Hs_IL38 FLCGPAEPQQPVQLTKESEPSARTKFYFEQSW
Bt_IL38 FLSGSSEPQQPLRLTKESEPSARTEFYFEQSR
```

Suppl. Fig. S5. Amino acid sequence alignment of interleukin-1 family (IL1F) proteins. The amino acid sequences of IL1F proteins from human (Hs, *Homo sapiens*), cattle (Bt, *Bos taurus*) and cetaceans (Tt, *Tursiops truncatus*; Oo, *Orcinus orca*; Lv, *Lipotes vexillifer*; Pc, *Physeter catodon*; Ba, *Balaenoptera acutorostrata scammoni*) were aligned using the Multalin algorithm. Amino acid residues conserved in all species are shown in red color, amino acid residues conserved in more than 50% of all species are marked in blue. Because of a gap in the genome sequence of the sperm whale (**A**) and the minke whale (**D**), the amino acid sequence prediction of IL1A (**A**) and IL18 (**D**) of these species is incomplete. The missing amino acid residues are indicated by X. Note that in IL36G* of the minke whale (Ba), a sequence gap corresponding to residues 103-105 of human IL36G is caused by the predicted use of a non-homologous splice site to compensate for the mutation of the ancestral splice site (**H**). As there is no evidence for the use of the non-homologous splice site, the Ba_IL36G* protein prediction is uncertain and absence of a functional IL36G gene in this species is possible.

A

```
                      E  I  F  F  A  L  A  S  S  L  S  S  A  S  A  E  K  G
Human           TTCCAGAGATCTTCTTTGCATTAGCCTCATCCTTGAGCTCAGCCTCTGCGGAGAAAGGAA
Minke whale     TTCCAGAGACCTTTTTTGTATCAGCCTCCCACGTGAGGTCAACTTGTGAGGAGAGAGGAA
Cattle          TTCCAGAAATCTTCTTTGTATTAGCCTCCCGTGTGAAGTCAGCTTATGAGAACAAAGGAA
                      E  I  F  F  V  L  A  S  R  V  K  S  A  Y  E  N  K  G


                S  P  I  L  L  G  V  S  K  G  E  F  C  L  Y  C  D  K  D  K
Human           GTCCGATTCTCCTGGGGGTCTCTAAAGGGGAGTTTTGTCTCTACTGTGACAAGGATAAAG
Minke whale     GCCTGATTCTCTTGGCCGTCTCTAAAGGCGAGCTGTGTCTCTTCTGTGACGTGAACAAAA
Cattle          GCCCCATTTTTCTGGCCGTCTCAAAAGGCCAGCTGTGTCTCTGCTGTGACACAAACAAAG
                S  P  I  F  L  A  V  S  K  G  Q  L  C  L  C  C  D  T  N


                G  Q  S  H  P  S  L  Q  L  K
Human           GACAAAGTCATCCATCCCTTCAGCTGAAGGTGAGAGT
Minke whale     GACAAAGCCAGCCATCC-TGCAGCTGAAGTTGAGCTT
Cattle          GACA---CAAGCCATCCCTGCAGCTGAAGGTGAGGGT
                G  H     K  P  S  L  Q  L  K
```

B

```
                 Q  S  S  S  G  S  A  F  R  L  E  A  A  A  W  P  G  W  F  L
Human           CAGAGCAGCTCAGGCTCCGCCTTCAGGCTTGAGGCTGCTGCCTGGCCTGGCTGGTTCCTG
Dolphin         CAATCTTATCCAACAGCCGCCTTCAGGCTGGAGGCTGCTTCCTGACCTGACTGGTTTCTC
Orca            CAATCTTATCCAACAGCCGCCTTCAGGCTGGAGGCTGCTTCCTGACCTGACTGGTTTCTC
Baiji           CAGAGACGCTCAGACCCCGCCTTCAGGCTGGAGGCTGCTGCCTGACCTGGCTGGTTCCTC
Sperm whale     CAGAGACGCTCGGACCCCGCCTTCAGGCTGGAGGCTGCTGCCTGACCTGGCTGGTTTCTC
Minke whale     CAGAGATGCTCGGACCCTGCCTTCAGGCTGGAGGCTGCTGCCTGACCTGGCTGGTTTCCC
Cattle          CAGAGAAGCTCAGGCCCTGCTTTCAGGCTTGAGGCTGCTGCCTGGCCTGGCTGGTTCCTC
                 Q  R  S  S  G  P  A  F  R  L  E  A  A  A  W  P  G  W  F  L
```

Suppl. Fig. S6. The *IL37* and *IL38* genes of cetaceans contain inactivating mutations. (**A**) Nucleotides sequences of the fifth coding exon of human (*Homo sapiens*), cattle (*Bos taurus*), and minke whale (*Balaenoptera acutorostrata scammoni*) *IL37* were aligned. Dashes were introduced to optimize the alignment. Nucleotides conserved in 3 species are highlighted by red letters, nucleotides conserved in 2 species are highlighted by blue letters. The translation of the human and cattle sequences are indicated by in the 1-letter amino acid code. A nucleotide deletion causing a reading frame shift and an in-frame stop codon are highlighted by black shading in the minke whale sequence. Intronic sequences (grey shading) are shown on each end of the exon. The sequence of human *IL37* gene corresponds to the nucleotide region 112913013 - 112918809 of human genomic DNA (GenBank accession number NC_000002.12). The location of the *IL37* gene of the minke whale is indicated in Suppl. Table S1. *IL37* of the baiji contains an inactivating mutation in another exon. (**B**) Nucleotides sequences within the 4th coding exon of the human (*Homo sapiens*) and cattle (*Bos taurus*) *IL38* and their orthologs in cetaceans were aligned. Amino acid sequences encoded by human and cattle *IL38* are shown above and below the corresponding nucleotide sequences. Nucleotides conserved in 3 species are highlighted by red letters, nucleotides conserved in more than 50% of the species are highlighted by blue letters. In-frame stop codons are highlighted by white letters on black background. The sequence corresponding to the nucleotide region 113072739 - 113075364 of human genomic DNA (GenBank accession number NC_000002.12) and the orthologous regions of other species are shown. The locations of the *IL38* genes of cetaceans are indicated in Suppl. Table S1.

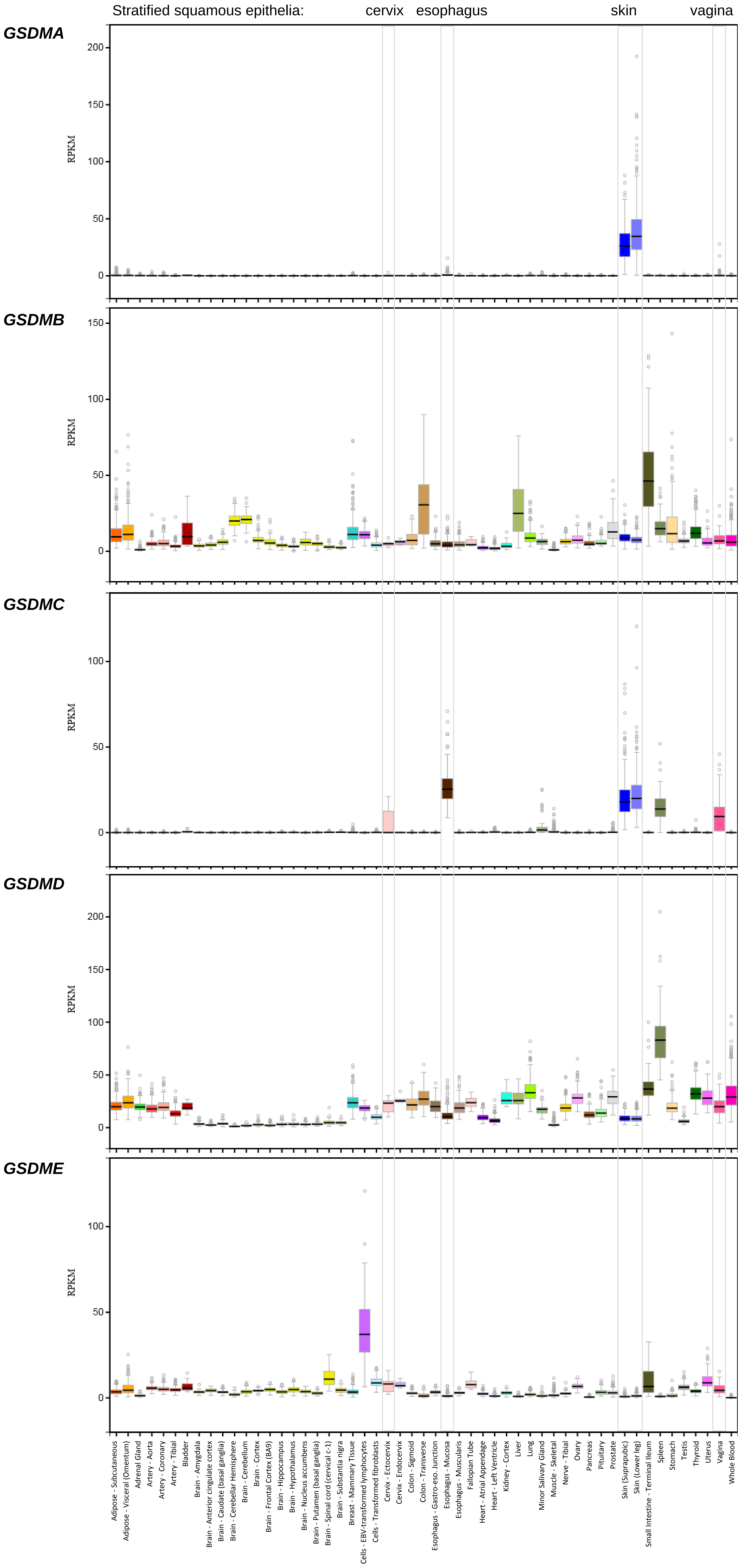

Suppl. Fig. S7. Expression levels of *GSDMA*, *GSDMB*, *GSDMC*, *GSDMD*, and *GSDME* in human organs, tissues, and cell types. The expression values are shown in RPKM (reads per kilobase of transcript per million mapped reads), calculated from a gene model with isoforms collapsed to a single gene. Box plots show the median and the 25th and 75th percentiles. Outliers are indicated. Organs with stratified squamous epithelia (cervix, esophagus, skin, vagina) are marked by grey boxes. Tissue sampling sites and tissue harvesting methods are described at: https://www.gtexportal.org/home/anatomogramPage. The data used for the analyses described in this manuscript were obtained from: GTEx Analysis Release V6p (dbGaP Accession phs000424.v6.p1) the GTEx Portal (https://www.gtexportal.org/) on 06/26/2017. These data are available without restrictions on use or publication. The Genotype-Tissue Expression (GTEx) Project was supported by the Common Fund of the Office of the Director of the National Institutes of Health, and by NCI, NHGRI, NHLBI, NIDA, NIMH, and NINDS.

A

```
          1                                                          60
Hs_GSDMA  MTMFENVTRALARQLNPRGDLTPLDSLIDFKRFHPFCLVLRKRKSTLFWGARYVRTDYTL
Bt_GSDMA  MTMFENVTRALTRQLNPRGDLTPLDSLIDFKRFHPFCLVLRKRKSTLFWGARYVCTDYTF

          61                                                        120
Hs_GSDMA  LDVLEPGSSPSDPTDTGNFGFKNMLDTRVEGDVDVPKTVKVKGTAGLSQNSTLEVQTLSV
Bt_GSDMA  LDILEPGSSPSDPTDSGNFGFKNMLDARVEGEVDVPKTVKVKGTAGLSRNSTLEVQTLSV

          121                                                       180
Hs_GSDMA  APKALETV-QERKLAADHPFLKEMQDQGENLYVVMEVVETVQEVTLERAGKAEACFSLPF
Bt_GSDMA  APKALETLHQERKLMAEHPFLEEMRRRGENLYVVMEVVEAVQEVTLERAGRAEGCFSLPF

          181                                                       240
Hs_GSDMA  FAPLGLQGSINHKEAVTIPKGCVLAFRVRQLMVKGKDEWDIPHICNDNMQTFPPGEKSGE
Bt_GSDMA  FAPLGLQGSVNHKEAVTIPKGCVLAFRVRQLMVKGKDEWDIPHIYNDNMHTFPPGEKPED

          241                                                       300
Hs_GSDMA  EKVILIQASDVGDVHEGFRTLKEEVQRETQQVEKLSRVGQSSLLSSLSKLLGKKKELQDL
Bt_GSDMA  EKFTLIQASDVGAVHEDFRTLKEEVQRETQEVEKLSPEGKSSLLSSLSKLLGKKKELQDL

          301                                                       360
Hs_GSDMA  ELALEGALDKGHEVTLEALPKDVLLSKEAVGAILYFVGALTELSEAQQKLLVKSMEKKIL
Bt_GSDMA  ELTLEEALGKGHEETLEALPKNVLLSKGAMDAILYFLGALAELSEAQQKLLVKSMEKKIL

          361                                                       420
Hs_GSDMA  PVQLKLVESTMEQNFLLDKEGVFPLQPELLSSLGDEELTLTEALVGLSGLEVQRSGPQYM
Bt_GSDMA  PVQLKLVESTMEQNFLQDKEGVFPLRPELLSSLGEEELTLTEALVGLSGLEVQRSGPQYT

          421                     446
Hs_GSDMA  WDPDTLPRLCALYAGLSLLQQLTKAS
Bt_GSDMA  WDPDTLPRLCALYAGLSLLRLLTKAS
```

Suppl. Fig. S8. Amino acid sequence alignment of gasdermins. Continued on the next page.

B

```
              1                                                         60
Hs_GSDMB      MFSVFEEITRIVVKEMDAGGDMIAVRSLVDADRFRCFHLVGEKRTFFGCRHYTTGLTLMD
Bt_GSDMB      MPTVFESISRAVVKEIDTSGETIAVRSLSDADKFHCSYLVKKRRRFFGYQYDKTNLTLKD

              61                                                       120
Hs_GSDMB      ILDTDG--DKWLDEL-----DSGLQ----------------------GQKAEFQILDNVD
Bt_GSDMB      ILECEVPFDMVVPELQGDLPDSGIKPASAALPEDSLPLSHLGNPHNKGQGDNFEILDVVD

              121                                                      180
Hs_GSDMB      STGELIVRLPKEITISGSFQGFHHQKIKISENRISQQYLATLENRKLKRELPFSFRSINT
Bt_GSDMB      SKGSLAVKFHPQMTFKIAFHIFQEKNIKLEKSEIPQEFLDSLKNKKLKKELPPSFQSIQA

              181                                                      240
Hs_GSDMB      -RENLYLVTETLETVKEETLKSDRQYKFWSQISQGHLSYKHKGQREVTIPPNRVLSYRVK
Bt_GSDMB      KREDLYLVTETLKTKRTETLKYETQFGFQILLKMFGFQCKHKHQKEVTITPEKVLAYRVK

              241                                                      300
Hs_GSDMB      QLVFPNKETM-------------KKDGASSCLGKSLGSEDSRNMKEKLEDMESVLKDLTE
Bt_GSDMB      QLVFPSAERMDICFLDKTRSFPEEKDGGSSWLGKSLNLENFRSIKERVQDMVRVLQDLTP

              301                                                      360
Hs_GSDMB      EKRKDVLNSLAKCLGKEDIRQDLEQRVSEVLISGELHMEDPDKPLLSSLFNAAGVLVEAR
Bt_GSDMB      EEQKEALNCFTKCLSSEEELQDLEQRVSEVQCSGELQMNSPANSLISSLFNAAGALIEAR

              361                                                      420
Hs_GSDMB      AKAILDFLDALLELSEEQQFVAEALEKGTLPLLKDQVKSVMEQNWDELASSPPDMDYDPE
Bt_GSDMB      AETIWDVLDALMELSEYRQFVAEVLDKGELRPVKDKVESILEENRCE---GPLDVSCDPE

              421                      446
Hs_GSDMB      ARILCALYVVVSILLELAEGPTSVSS
Bt_GSDMB      ARTLCALYVVVSILLQLSEKPTSVSS
```

Suppl. Fig. S8. Amino acid sequence alignment of gasdermins. Continued on the next page.

C

```
           1                                                          60
Hs_GSDMC   MPSMLERISKNLVKEIGSKDLTPVKYLLSATKLRQFVILRKKKDSRSSFWEQSDYVPVEF
Bt_GSDMC   MPSLFEHTSKNLVKELGDKDFRPLQNLLSAHKFCQLKLLRKKRRTLSQFWEQPD-VPVDH

           61                                                        120
Hs_GSDMC   SLNDILEPSSSVLETVVTGPFHFSDIMIQKHKADMGVNVGIEVSVSGEASVDHGCSLEFQ
Bt_GSDMC   TLTDILEPSPSVPEPVLSKKFIFIDKTVWKGAAEVDVTAGLEVSVSGTATQSCECSLEVQ

           121                                                       180
Hs_GSDMC   IVTIPSPNLEDFQKRKLLDPEPSFLKECRRRGDNLYVVTEAVELINNTVLYDSSSVNILG
Bt_GSDMC   SVTISPWDWEDLQKRKVLDQEPSFLQECRTRGDNLYVVTEAVKLVNETVLQDSSSVNATG

           181                                                       240
Hs_GSDMC   KIAL-WITYGKGQGQGESLRVKKKALTLQKGMVMAYKRKQLVIKEKA---ILISDDDEQR
Bt_GSDMC   TFSIPWSFYAKSTAEGSGLKERKRTMTVPQGTVMAYKKKQLVFREDGRAILLISDDDKQK

           241                                                       300
Hs_GSDMC   TFQDEYEISEMVGYCAARSEGLLPSFHTISPTLFNASSNDMKLKPELFLTQQFLSGHLPK
Bt_GSDMC   TFPE------------IKLQGL-----PLSPEILSISDDN---KRKAFFVGKVQRQSAMG

           301                                                       360
Hs_GSDMC   YEQVHILPVGRIEEPFWQNFKHLQEEVFQKIKTLAQLSKDVQDVMFYSILAMLRDRGALQ
Bt_GSDMC   TKRRPILPIGRIQEPISRDFKSLQNEVSREIEAVAELPRDIRDALFHTILAKLKDQGALQ

           361                                                       420
Hs_GSDMC   DLMNMLELDSSGHLDGPGGAILKKLQQDSNHAWFNPKDPILYLLEAIMVLSDFQHDLLAC
Bt_GSDMC   DLTDMLD----GNLWDHTGSFLSEMREDSRNVWLESRPCVIYLLEALLVLSDIQHELLAW

           421                                                       480
Hs_GSDMC   SMEKRILLQQQELVRSILEPNFRYPWSIPFTLKPELLAPLQSEGLAITYGLLEECGLRME
Bt_GSDMC   SMEKRILPQQRELVESILEPNFRYPWNIPFTLDPKLLASLQDEGLAVTFGLLQECGLRVA

           481                             512
Hs_GSDMC   LDNPRSTWDVEAKMPLSALYGTLSLLQQLAEA
Bt_GSDMC   PDNPKGTWDLEAKKPLSALYGSLSVLQQLAEA
```

Suppl. Fig. S8. Amino acid sequence alignment of gasdermins. Continued on the next page.

D

```
         1                                                          60
Hs_GSDMD MGSAFERVVRRVVQELDHGGEFIPVTSLQSSTGFQPYCLVVRKPSSSWFWKPRYKCVNLS
Bt_GSDMD MASAFEKVVRSVVRELDHK-DLTPVDSLWSSTSFQPYTLLSRKPLSSRFWRPRYKCVNLS
Tt_GSDMD MASAFARVVRSVVQELDHGGELTPVDSLQSSTSFQLYCLLGRKSSSSRFWKHRYTRVNLS
Oo_GSDMD MASAFARVVRSVVQELDHGGELTPVDSLQSSTSFQLYCLLGRKSSSSRFWKHRYTRVNLS
Lv_GSDMD MASAFARVARSVVQELDHGGELTPVDSLQSSTSFQLYCLLGRKSSSSRFWKHRYTRVNLS
Pc_GSDMD MAAAFARVVKSVVRELDHGGELTPVDSLQSSSSFQLYCLLGRKPSSSRFWRHRYTRVNLS
Ba_GSDMD MASAFARVVKSVVRELDHSGELTPVDSLQSSTSFQLYCLLGRRSSSSRFWRHRYTRVNLS

         61                                                        120
Hs_GSDMD IKDILEPDAAEPDVQRGRSFHFYDAMDGQIQGSVELAAPGQAKIAGGAAVSDSSSTSMNV
Bt_GSDMD IRDILEPDAPEPALECGRTFQFHDAMDGQLQGSVKLAAPGQGRLSGGAAVSGSSSASMDL
Tt_GSDMD IRDILEPDAPEPAVECGNTFHFHDAMDGKMQGSVELAAPGQGKLSGGAAVSGSSSASMIV
Oo_GSDMD IRDILEPDAPEPAVECGNTFHFHDAMDGKMQGSVELAAPGQGKLSGGAAVSGSSSASMIV
Lv_GSDMD IRDILEPDAPEPAVECGSTFHFHDAMDGQLQGSVELAAPGQGKLSGGAAVSGSSSASMIV
Pc_GSDMD IRDILEPDAPEPAVECGSTFHFHDATDGQLQGSVELAAPGQGKLSGGAAVSGSSSASMNV
Ba_GSDMD IRDILEPDAPEPAVERGSTFHFHDAMDGQLQGSVELAAPGQGKLSGGAAVSGSSSTSMHV

         121                                                       180
Hs_GSDMD YSLSVDPNTWQTLLHERHLRQPEHKVLQQLRSRGDNVYVVTEVLQTQKEVEVTRTHKREG
Bt_GSDMD CTLRVTPNTWEAMHHERRLRQPEPKTLQQLRSRGDDVFVVTEVLQTQKEVEVTRTHKQEG
Tt_GSDMD CTLRVAPNTWEAMHRERRLRRPEHKVLQQLRNRGDDVFVVTEVLQTQQEVEVTRTQKQEG
Oo_GSDMD CTLRVAPNTWEAMHRERRLRRPEHKVLQQLRNRGDDVFVVTEVLQTQQEVEVTRTQKQEG
Lv_GSDMD CMLRVAPNTWEAMHRERRLRQPEHKVLQQLRNRGDDVFVVTEVLQTQQEVEVTRTQKQEG
Pc_GSDMD CVLRVAPNTWEAMHRERRLRQPEHKVLQQLRNRGHDVFVVTEVLQTQKEVEVTRTQKQEG
Ba_GSDMD CMLRVAPNTWEAMHRERRLRQPEHKILQQLRNRGYDVFVVTEVLQTQKEAEVTRTQKQEG

         181                                                       240
Hs_GSDMD SGRFSLPGATCLQGEGQGHLSQKKTVTIPSGSTLAFRVAQLVIDSDLDVLLFPDKKQRTF
Bt_GSDMD SGQFALPGAFCLQGKGEGHLSQKKTVTIPSGSTLAFRAAQLVIGSDWDILLFPDKKQRTF
Tt_GSDMD SGQFALPGAMGLQGRGEGHLSQNKMVTIPAGSILAFRVAQLVIGSDWDILFFPDKKQTTF
Oo_GSDMD SGQFALPGAMGLQGRGEGHLSQNKMVTIPAGSILAFRVAQLVIGSDWDILFFPDKKQTTF
Lv_GSDMD SGQFALPGAMCLQGRGEGHLSQKKMVTIPSGSILAFRVAQLVIDSDWDILFFPDKKQTTF
Pc_GSDMD SGQFAFPGATCLQGRGEGHLSQKKMVTIPSGSILAFRVAQLVIGPDWDILFFPDKKQTTF
Ba_GSDMD SGQFALPGAMCLQGRGEGHLSQKKMVTIPSGSILAFRVAQLVIGPDWDILFFPDKKQTTF

         241                                                       300
Hs_GSDMD QPPATGHKRSTSEGAWPQLPSGLSMMRCLH---NFLTDG-VPAEGAFTEDFQGLRAEVET
Bt_GSDMD LSLQAGRRPSSSADSHPHPCFSLASIRALSDYFHFQPDGSTEHWLATTDDYQGLRAEVKA
Tt_GSDMD RPQQEDYRPSYIAGGQLQRSSCLTSTKFLSLRFKFLSDGPMEDRLVTTEGFQGLQAEVEA
Oo_GSDMD RPQQEDYRPSYIAGGQLQRSSCLTSTKFLSLRFKFLSDGPMEDRLATTEGFQGLQAEVEA
Lv_GSDMD RPQQKGYRPSYIAGGQPQRSSCLASMRFFSVHFKFLSDGPVEDRLAITEDFQGLQAEVEA
Pc_GSDMD RPQQEGYRPSYVAGGQLQQSSCLASIRFLSAHFRFLSDGPVEDRLAITEDFQGLQAEVGA
Ba_GSDMD RPQQQGYGPSYIAGGQLPWSSCLTSIRFPSVHFKFLSDGPVEGRLATTEDFQGLQAEVGA

         301                                                       360
Hs_GSDMD ISKELELLDRELCQLLLEGLEGVLRDQLALRALEEALEQGQSLGPVEPLDGPAGAVLECL
Bt_GSDMD WAMGLEGLSKGLCGQLLGGLGQVLRDEPALQALEDSLEQGLCSGPVEPRDGPVGAILESL
Tt_GSDMD WAVGLEGLSREPCRQLLGALGQVLRDEAALQALDESLEHGLRGGLLESRDGPVGAVLECL
Oo_GSDMD WAVGLEGLSREPCRQLLGALGQVLRDEAALQALDESLEHGLRGGLLESRDGPVGAVLECL
Lv_GSDMD WAVGLEGLSREPCGQLLGALGQVLRDEAALQALDESLEHGLRGGLLEPRDGPVGAVLECL
Pc_GSDMD WAVGLNGLSKEPCGQLLGALGQVLRDEAALQALDESLEHGLCGGLLEPRDGPVGAVLECL
Ba_GSDMD WAVGLEGLSKEPCGQLLGALGQVLRDKAALQALDESVSEXXXXXXXXXXXXXXXXXXXXX
```

Suppl. Fig. S8. Amino acid sequence alignment of gasdermins. Continued on the next page.

```
             361                                                         420
Hs_GSDMD     VLSSGMLVPELAIPVVYLLGALTMLSETQHKLLAEALESQTLLGPLELVGSLLEQSAPWQ
Bt_GSDMD     VLPSGQLETELAGPVFYLLQALAVLSEAQHVLLAEVLEMGALSGTFWLVESLVEQSSPWQ
Tt_GSDMD     VFSSRRLEKRLAGPVFYLLQALAVLSATQHVLLAEVLEMGALSGAFKLVESLLEQSTPWQ
Oo_GSDMD     VFSSRRLEKRLAGPVFYLLQALAVLSATQHVLLAEVLEMGALSGAFKLVESLLEQSTPWQ
Lv_GSDMD     VPSSRRLEEKLAGPVFYLLQALAVLSATQHVLLAEVLEMGALSGAFKLVESLLEQSTPWQ
Pc_GSDMD     VFSSRRLEEELAGPVFYLLQALAVLSATQHVLLAEVLEMGALSGAFKLVESLLEQSTPWQ
Ba_GSDMD     XXXXXXXXXXXXXXXXXXXXXXLALSATQHVLLAEVLETGALSGTFKLVESLLEQSTPWQ

             421                                                         480
Hs_GSDMD     ERSTMSLPPGLLGNSWGEGAPAWVLLDECGLELGEDTPHVCWEPQAQGRMCALYASLALL
Bt_GSDMD     EHRAVSLPPERLGDSWGSEAPGWALLEACGLEPQVGTPQVCWEPGARGCACALYACLALL
Tt_GSDMD     EHRAVSLPHELLGSSWGSEAPTWVLLEECGLEPQVGAPQVCWKPEAQGCANALYACLTLL
Oo_GSDMD     EHRAVSLPHELLGSSWGSEAPTWVLLEECGLEPQVGAPQVCWKPEAQGCANALYACLALL
Lv_GSDMD     ERRAVSLPPELLGSSWGSEAPTWVLLEECGLEPQVGAPQVCWKPEAQGCACALYACLALL
Pc_GSDMD     ECRAVSLPAELLGDSWGSEAPTWVLLEECGLEPQVGAPQVCWKPEAQGCAYALYACLALL
Ba_GSDMD     ERRAVSLPPELLGSSWDSEAPTWVLLEECGLEPQVGAPQVCWKPEAQGCACALYACLALL

             481                                             529
Hs_GSDMD     SGLSQEPH-----------------------------------------
Bt_GSDMD     LRLSQLC------------------------------------------
Tt_GSDMD     FRLSRLC------------------------------------------
Oo_GSDMD     FRLSRLC------------------------------------------
Lv_GSDMD     FRLSRLC------------------------------------------
Pc_GSDMD     FRLSRLC------------------------------------------
Ba_GSDMD     FRLSRLCQPAVRPQGSSPVWAELTPTQLPGATPSHLGSSPQGLGVEESQ
```

Suppl. Fig. S8. Amino acid sequence alignment of gasdermins. Continued on the next page.

E

```
          1                                                          60
Hs_GSDME  MFAKATRNFLREVDADGDLIAVSNLNDSDKLQLLSLVTKKKRFWCWQRPKYQFLSLTLGD
Bt_GSDME  MFAKATRNFLKEVDAGGNLIAVSNLNDSDKLQLLSLVTKKKRYWCWQRPKYQFLSVTLGD
Tt_GSDME  MFAKATRNFLREVDAGGNLITVSNLNDSDKLQLLSLVTKKKRLWCWQRPKYQFLSVTLGD
Oo_GSDME  MFAKATRNFLREVDAGGNLITVSNLNDSDKLQLLSLVTKKKRLWCWQRPKYQFLSVTLGD
Lv_GSDME  MFAKATRNFLREVDAGGNLITVSNLNDSDKLQLLSLVTKKKRLWCWQRPKYQFLSVTLGD

          61                                                        120
Hs_GSDME  VLIEDQFPSPVVVESDFVKYEGKFANHVSGTLETALGKVKLNLGGSSRVESQSSFGTLRK
Bt_GSDME  VLTEDQFLSPVVVESDFVKYEGKFENHVSGTLETALGKVKLNIGGKGLVESQSSFGTLRK
Tt_GSDME  VLTEDQFLSPVVVESDFVKYEGKFENHVSGTLETALGKVKLNIGGKGLVESQSSFGTLRK
Oo_GSDME  VLTEDQFLSPVVVESDFVKYEGKFENHVSGTLETALGKVKLNIGGKGLVESQSSFGTLRK
Lv_GSDME  VLTEDQFLSPVVVESDFVNYEGKFENHVSGTLETAMGKVKLNLGGKGLVESQSSFGTLRK

          121                                                       180
Hs_GSDME  QEVDLQQLIRDSAERTINLRNPVLQQVLEGRNEVLCVLTQKITTMQKCVISEHMQVEEKC
Bt_GSDME  QEVDLQQLIGDAQERTINLKNPVLQQVLERKNAVLCVLTQKIVTTQKCVISEHVQIEETC
Tt_GSDME  QEVDLQQLIGVAQERTINLKNSVLQQVLERKNEVLCVLTQKIVTTRKCVISEHVQIEEKC
Oo_GSDME  QEVDLQQLIGVAQERTINLKNSVLQQVLERKNEVLCVLTQKIVTTRKCVISEHVQIEEKC
Lv_GSDME  QEVDLQQLIGVAQERTINLKNSVLQQVLERKNEVLCVLTQKIVTTRKCVISEHVRIEEKC

          181                                                       240
Hs_GSDME  GGIVGIQTKTVQVSATEDGNVTKDSNVVLEIPAATTIAYGVIELYVKLDGQFEFCLLRGK
Bt_GSDME  GGMVGIQTRTVQVSAMEDGNIIKDTNVVLEIPAPTTIAYGVIELYVRADGQFEFCLLQGK
Tt_GSDME  GGMVGIQTKTVQVSATEDGNIIKDSNVVLEIPAPTTIAYGVIELYVRADGQFEFCLLQGK
Oo_GSDME  GGMVGIQTKTVQVSATEDGNIIKDSNVVLEIPAPTTIAYGVIELYVRADGQFEFCLLQGK
Lv_GSDME  GGTVGIQTKTVQVSATEDGNIIKDSNVVLEIPAPTTIAYGVIELYVRADGQFEFCLLQGK

          241                                                       300
Hs_GSDME  QGGFENKKRIDSVYLDPLVFREFAFIDMPDAAHGISSQDGPLSVLKQATLLLERNFHPFA
Bt_GSDME  HGGFEQERRSNSVLLDPLPFREFVFWDMPDAGQGLPAQDKPLSVLKQATLLLERNFHPFM
Tt_GSDME  HGGFEHERRSDSAFLDLLPLREFAFWDTPDAGQGLPASDGPLSVLKQAPLFLERNFHPFV
Oo_GSDME  HGGFEHERRSDSAFLDLLPLREFAFWDTPDAGQGLPASDGPLSVLKQAPLFLERNFHPFV
Lv_GSDME  HGGFEHERRSVSAFLDLLPLQEFAFWDTPDAGQGLPASDGPLSVLKQATLFLERNFHPFA

          301                                                       360
Hs_GSDME  ELPEPQQTALSDIFQAVLFDDELLMVLEPVCDDLVSGLSPTVAVLGELKPRQQQDLVAFL
Bt_GSDME  ELPEQQQIALNDVLQAVLLDEELLVVLEQVCDDIVSSLSPSLVALGELKPSQKHNLTTFL
Tt_GSDME  ELPEQQQRALNDVLQAVLFDEELLLVLEQVCEDIVSSLSPSLAVLGKLKPSQQRDLVAFL
Oo_GSDME  ELPEQQQRALNDVLQAVLFDEELLLVLEQVCEDIVSSLSPSLAVLGKLKPSQQRDLVAFL
Lv_GSDME  ELPEQQQRALNDVLQAVLFDEELLLVLEQVCEDIVSSLSPSLAVLGKLKPSQQRDLVAFL

          361                                                       420
Hs_GSDME  QLVGCSLQGGCPGPEDA-GSKQLFMTAYFLVSALAEMPDSAAALLGTCCKLQIIPTLCHL
Bt_GSDME  RLVGCSVQGERLGSQDVVDNQKLFSTAFFLVSALAEMPDNAAALLGTCCKLQIIPLLYHL
Tt_GSDME  RLVGYSVQGECPGPENVVHNQKLLSTAYFLVSALAEMPDNAAGLLGTCCKLQIIPTLCHL
Oo_GSDME  RLVGYSVQGECPGPEDVVHNQKLLSTAYFLVSALAEMPDNAAGLLGTCCKLQIIPTLCHL
Lv_GSDME  RLVGYSVQGECPGPEDVVHNQELLSTAYFLVSALAEMPDNAAVLLGTCCKLQIIPTLCHL
```

Suppl. Fig. S8. Amino acid sequence alignment of gasdermins. Continued on the next page.

```
            421                                                          480
Hs_GSDME    LRALSDDGVSDLEDPTLTPLKDTERFGIVQRLFASADISLERLKSSVKAVILKDSKVFPL
Bt_GSDME    LRALSHDGVSDLEDPALAPLKDREKFGIVQRLFAATDINLERMQSSVKAASREDPNVLPL
Tt_GSDME    LCALSDDGVSDLEDPVLAPLKDTERFGIVQRLFASADINLERLQPSVKVTTRKDPNVFPL
Oo_GSDME    LHALSDDGVSDLEDPVLAPLKDTERFGIVQRLFASADINLERLQPSVKVATRKDPNVFPL
Lv_GSDME    LRALSDDGVSDLEDPVLAPLKDTERFGIVQRLFASADINLERLQPSVKAATRKDPNVFPL

            481             497
Hs_GSDME    LLCITLNGLCALGREHS
Bt_GSDME    ILYISLSGLCALGRAQ-
Tt_GSDME    ILYISLNGLCALGRAQ-
Oo_GSDME    ILYISLNGLSALGRAQ-
Lv_GSDME    ILYISLNGLCALGRAR-
```

Suppl. Fig. S8. Amino acid sequence alignment of gasdermins (GSDMs). The amino acid sequences of gasdermin proteins from human (Hs, *Homo sapiens*), cattle (Bt, *Bos taurus*) and cetaceans (Tt, *Tursiops truncatus*; Oo, *Orcinus orca*; Lv, *Lipotes vexillifer*; Pc, *Physeter catodon*; Ba, *Balaenoptera acutorostrata scammoni*) were aligned with the Multalin algorithm. Amino acid residues conserved in all species are shown in red color, amino acid residues conserved in more than 50% of all species are marked in blue. Because of a gap in the genome sequence of the minke whale, the amino acid sequence of GSDMD of this species could not be predicted completely (**D**). Missing amino acid residues are indicated by X. Also in the minke whale, the ancestral stop codon of GSDMD is mutated, leading to an extension of the coding sequence on the C-terminus (**D**). It is unknown whether this elongation of the protein is compatible with the normal function of GSDMD.

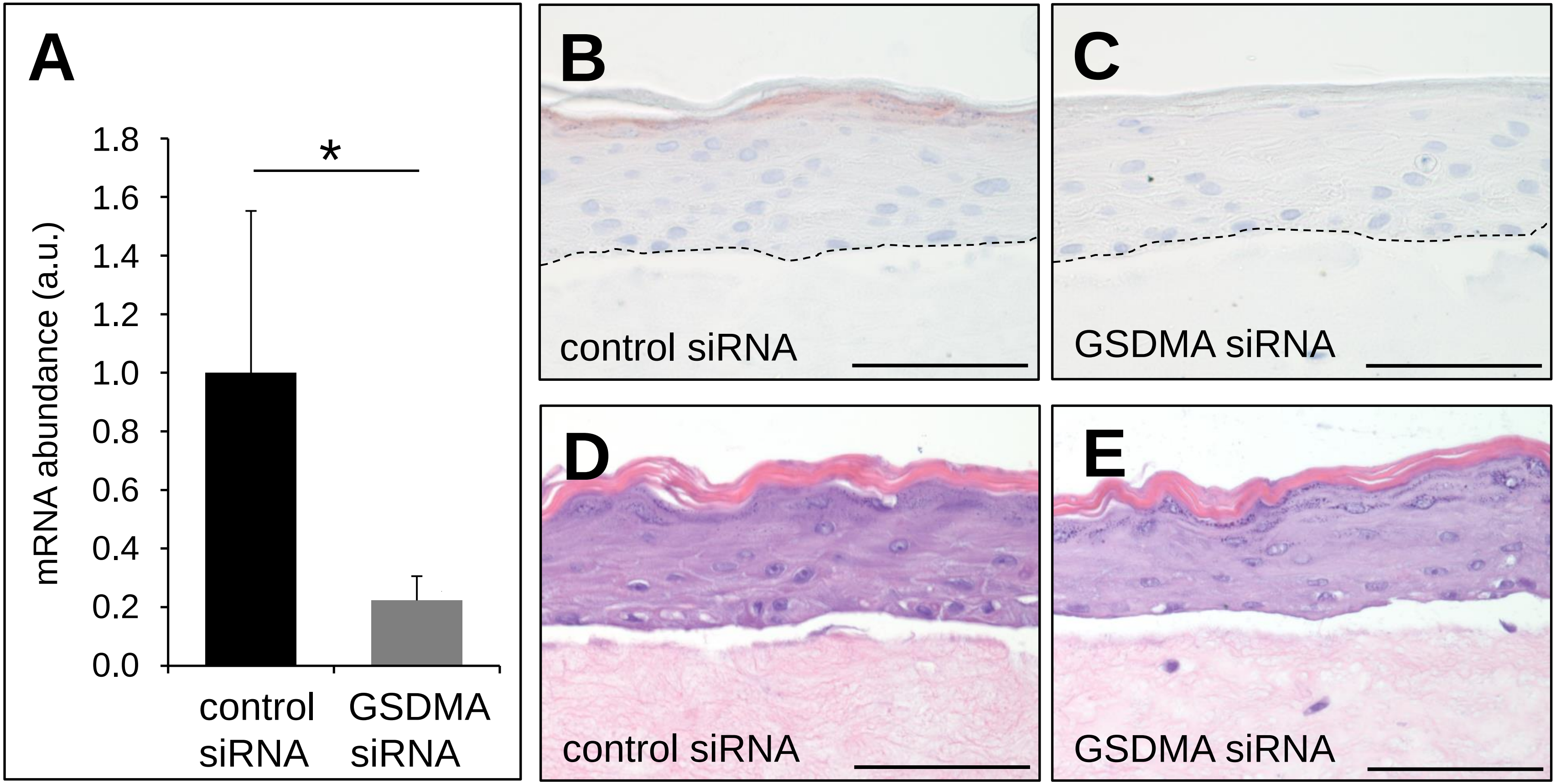

Suppl. Fig. S9. Knockdown of *GSDMA* in human skin equivalents does not suppress cornification. (**A**) Quantitative RT-PCR analysis of *GSDMA* expression in keratinocytes treated with control siRNA or siRNA specific for *GSDMA*. The *GSDMA* mRNA abundance was normalized to that of the house-keeping gene *B2M*. Bars show the mean (n=4) and error bars show standard deviations. *, $p<0.05$ (t-test). a.u., arbitrary units. (**B, C**) Immunohistochemical detection of GSDMA (red) in skin equivalents comprising keratinocytes treated with control siRNA or *GSDMA*-specific siRNA. The epidermal-dermal junction is indicated by a broken line. (**D, E**) Hematoxylin and eosin staining of skin equivalents comprising keratinocytes treated with control siRNA or *GSDMA*-specific siRNA. Note that the stratum corneum devoid of nuclei is present in control and *GSDMA* knockdown skin equivalents. Scale bars, 100 µm.

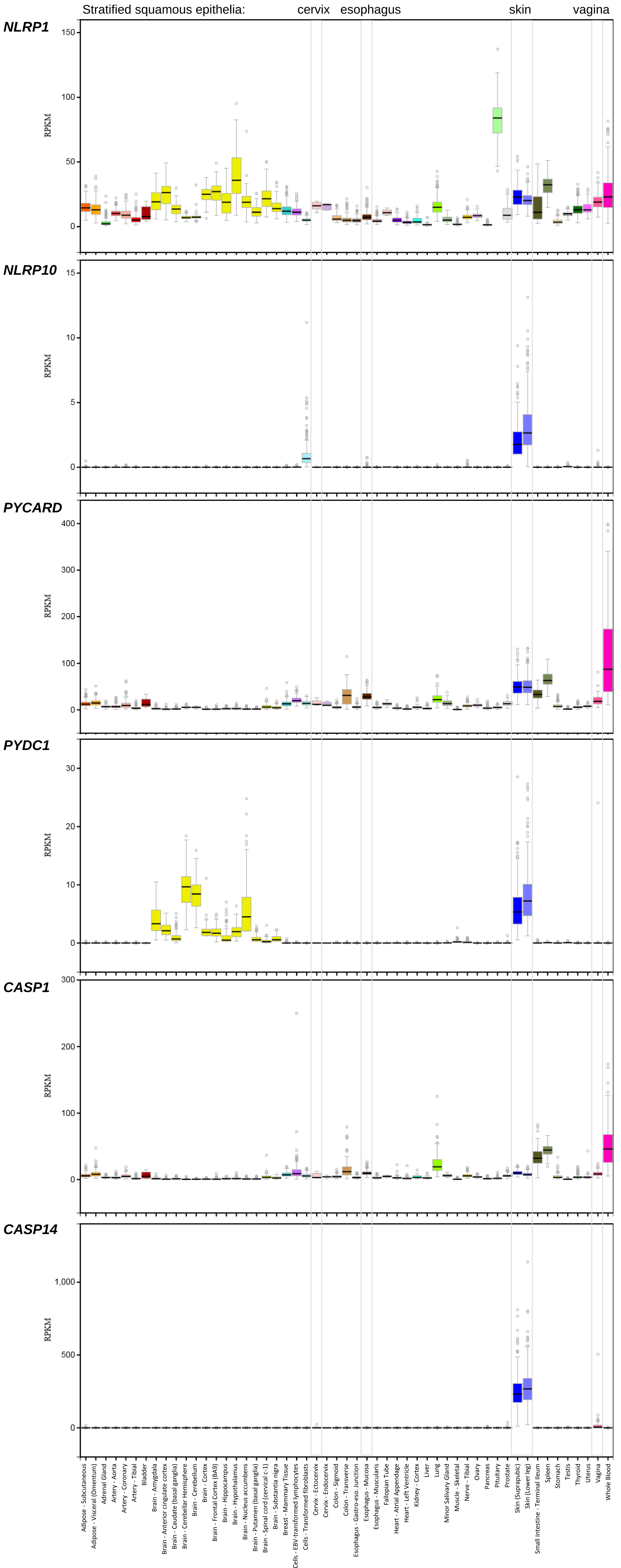

Suppl. Fig. S10. Expression levels of *NLRP1*, *NLRP10*, *PYCARD*, *PYDC1*, *CASP1*, and *CASP14* in human organs, tissues, and cell types. The expression values are shown in RPKM (reads per kilobase of transcript per million mapped reads), calculated from a gene model with isoforms collapsed to a single gene. Box plots show the median and the 25th and 75th percentiles. Outliers are indicated. Organs containing stratified squamous epithelia (cervix, esophagus, skin, vagina) are marked by grey boxes. Tissue sampling sites and tissue harvesting methods are described at: https://www.gtexportal.org/home/anatomogramPage. The data used for the analyses described in this manuscript were obtained from: GTEx Analysis Release V6p (dbGaP Accession phs000424.v6.p1) the GTEx Portal (https://www.gtexportal.org/) on 06/26/2017. These data are available without restrictions on use or publication. The Genotype-Tissue Expression (GTEx) Project was supported by the Common Fund of the Office of the Director of the National Institutes of Health, and by NCI, NHGRI, NHLBI, NIDA, NIMH, and NINDS (GTEx Consortium. Science. 2015;348:648-660. doi: 10.1126/science.1262110).

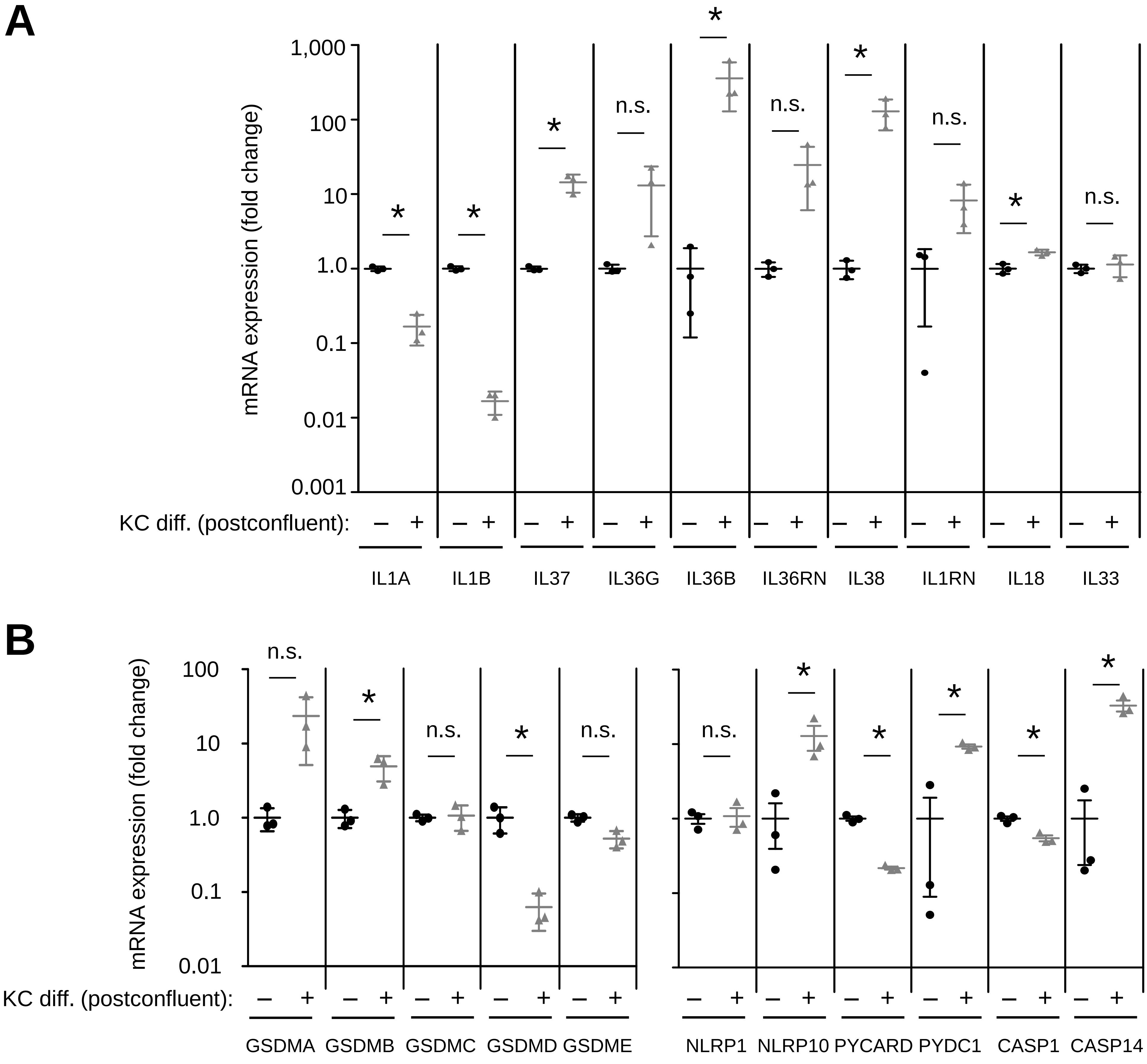

Suppl. Fig. S11. Gene expression analysis in subconfluent (undifferentiated) versus postconfluent (differentiated) keratinocytes. Human keratinocytes (KC) were cultured in subconfluent monolayer culture under conditions that do not (-) induce differentiation and in postconfluent culture (until day 7 after reaching confluence) which induces differentiation (+) (n=3). Note that the differentiation program is less complete in postconfluent cell cultures than in skin equivalent cultures. Quantitative RT-PCR analyses of the indicated genes of the interleukin-1 family (**A**) and of pyroptosis-related gene families (**B**) were performed as described in Materials and Methods. Individual values are indicated by circles and squares; the horizontal line indicates the mean, and error bars indicate the standard deviation. Note the logarithmic scale on vertical axes. *, p<0.05 (two-sided t-test); n.s., not significant.

A

```
               1                                                          60
Hs_PYCARD      MGRARDAILDALENLTAEELKKFKLKLLSVPLREGYGRIPRGALLSMDALDLTDKLVSFY
Bt_PYCARD      MGCTRDAILDALENLTADELKKFKMKLLSVPLREGYGRIPRGTLLPLDAVDLTDKLVSYY
Tt_PYCARD      MGCTRDAILDALENLTADEFKKFKMKLLSVPLREGYGRIPRGTLLPMDAVDLTDKLVSYY
Oo_PYCARD      MGCTRDAILDALENLTADEFKKFKMKLLSVPLREGYGRIPRGTLLPMDAVDLTDKLVSYY
Lv_PYCARD      MGCTRDAILDALENLTADEFKKFKMKLLSVPLREGYGRIPRGTLLPMDAVDLTDKLVSYY
Pc_PYCARD      MGCTRDAILAALENLTADEFKKFKMKLLSVPLREGYGRIPRGTLLPMDAMDLTDKLVSYY
Ba_PYCARD      MGCTRDAILDALENLTADEFKKFKMKLVSVPLREGYGRIPRGTLLPMDPVDLTDKLVSYY

               61                                                        120
Hs_PYCARD      LETYGAELTANVLRDMGLQEMAGQLQAATHQGSGAAPAGIQAPPQSAAKPGLHFIDQHRA
Bt_PYCARD      LEAYGAELTALVLRDMGMQEVAEQLQETMSKGPRNVLAEVRDPLQKTAKPGLHFVDQHRA
Tt_PYCARD      LEAYSAELTVFVLCNIGMQEVAEQLQETLCKGPGTTPAGIKAPPQTAAKPALHFVEQHRA
Oo_PYCARD      LEAYSAELTVFVLCNIGMQEVAEQLQETLRKGPGTTPAGIKAPPQTAAKPALHFVEQHRA
Lv_PYCARD      LEAYSAELTILVLCNIGMQEVAEQLQETLRKGPGTTPAGIKAPPQTAAKPALHFVEQHRA
Pc_PYCARD      LEAYGAELTVLVLCNIGMQEVAEQLQETLRKGPGTTPAGIKAPPQTAAKPALHFVDQHRA
Ba_PYCARD      LEAYGAELTVLVLCNMGMQEVAEQLQETLRKGPGTTPAGIKEPPQTAAKPALHFVDQHRA

               121                                                       180
Hs_PYCARD      ALIARVTNVEWLLDALYGKVLTDEQYQAVRAEPTNPSKMRKLFSFTPAWNWTCKDLLLQA
Bt_PYCARD      ALIARVTVVDGVLDALYGKVLTEEQYQAVRAERTSSDKMRKLFSFSPAWNMTCKDLLLQA
Tt_PYCARD      ALIARVTDVDGVLDALYGKVLTEDQYQAVRAECTNSNKMRKLFSFAPAWNLTCKNLLLQA
Oo_PYCARD      ALIARVTDVDGVLDALYGKVLTEDQYQAVRAECTNSNKMRKLFSFAPAWNLTCKNLLLQA
Lv_PYCARD      ALIARVTDVDGVLDALYGKVLTEEQYQAVRAERTNPTKMRKLFSFAPAWNLTCKDLLLQA
Pc_PYCARD      ALIARVTAVDGVLDALYGKVLTEEQYQTVRAERTNPTKMRKLFRFAPAWNLACKDLLLQA
Ba_PYCARD      ALIARVTDVDGMLDALYGKVLTEQQYQAVRAERTNPTKMRMLFSFAPAWNLTCKDLLLQA

               181         195
Hs_PYCARD      LRESQSYLVEDLERS
Bt_PYCARD      LRDTQPYLVDDLEQS
Tt_PYCARD      LRDTQPYLVVDLEQS
Oo_PYCARD      LRDTQPYLVVDLEQS
Lv_PYCARD      LRDTQPYLVVDLEQS
Pc_PYCARD      LRDTQPYLVVDLEQS
Ba_PYCARD      LRDTQPYLVVDLEQS
```

B

```
               1                                                          60
Hs_CASP1       MADKVLKEKRKLFIRSMGEGTINGLLDELLQTRVLNKEEMEKVKRENATVMDKTRALIDS
Bt_CASP1       MADKLLKEKRKLFVHSVSKGTINGLLDELLEKRVLNQEEMEKIRDENDTAMDRARVLIDT
Tt_CASP1       MADKVLKEKRKLFVHSVGLGTINGLLDELLEKRVLNQEEMEKVRDENATVMDKARALIDS
Oo_CASP1       MADKVLKEKRKLFVHSVGMGTINGLLDELLEKRVLNQQEMEKVRDENATVMDKARALIDS
Lv_CASP1       MADKVLKEKRKLFVHSVGMGTINGLLDELLEKRVLNQEEMEKVRDENATVMDKARALIDS
Pc_CASP1       MADKVLKEKRKLFVHSVSMGTINGLLDELLEKRVLNQEEMEKVRDENATVMDKARALIDS
Ba_CASP1       MADKVLKEKRNLFVHSVGMGTINGLLDELLEKRVLNQAEMEKVRDENATVMDKARALIDS
```

Suppl. Fig. S12. Amino acid sequence alignment of pyroptosis-related proteins. Continued on the next page.

```
         61                                                         120
Hs_CASP1 VIPKGAQACQICITYICEEDSYLAGTLGLSADQTSGNYLNMQDSQGVLSSFPAPQAVQDN
Bt_CASP1 VIRKGPQACQICIGHICEEDSHLAGILGLTSGSQSENYLR-QKPQAVVPPFPAPQAMLDN
Tt_CASP1 VIRKGPQACQICISHIREDDSLLAGTLGLSSGSQSGNYLNIQESQVVVPPFPAPQAMQDN
Oo_CASP1 VIRKGPQACQICISHIREDDSLLAGTLGLSSGSQSGNYLNTQESQVVVPPFPAPQAMQDN
Lv_CASP1 VIRKGPQACQICISHISEDDSLLAGTLGLSSGSQSGNYLNTQESQVVFPPFPAPQAMQDN
Pc_CASP1 VIRKGPQACQICIRHISEDDSLLAGTLGLSSGSQSGNFLNTQESQAVVPPFPAPQAMQDN
Ba_CASP1 VIPKGSQACQICISHIREDDSLLAGTLGLSSGSQSGNYLNTQESQAVVPPFPAPQAMQDN

         121                                                        180
Hs_CASP1 PAMPTSSGSEGNVKLCSLEEAQRIWKQKSAEIYPIMDKSSRTRLALIICNEEFDSIPRRT
Bt_CASP1 PVKLASSGPGGNLKLCPPETAQRIRKEKSGEIYPIMERSNRTRLALIICNTEFENLTRRD
Tt_CASP1 PVKFASSGPGGNLKLCPLETAQRIWKEKSAEIYPIMERSIRTRIALIICNTEFENLPRRD
Oo_CASP1 PVKLASSGPGGNLKLCPLETAQRIWKEKSAEIYPIMERSIRTRIALIICNTEFENLPRRD
Lv_CASP1 PVKLAPSGPGGNLKLCPLETAQRIWKEKSAEIYPIMERSVRTRIALIICNTEFENLPRRD
Pc_CASP1 PVKLASSGPGGNLKLCPLETAQRIWKEKSAEIYPIMERSIRTRIALIICNTEFENLPRRD
Ba_CASP1 PVKLASSGPEGNLKLCPLETAQRIWKEKSAEIYPIMERSIRTRIALIICNTEFENLPRRD

         181                                                        240
Hs_CASP1 GAEVDITGMTMLLQNLGYSVDVKKNLTASDMTTELEAFAHRPEHKTSDSTFLVFMSHGIR
Bt_CASP1 GADVDTRNMKVLLEGLGYKVDVKENLTASEMILELKAFAAHSEHRTSDSTFLVFMSHGIR
Tt_CASP1 GADVDIRNMKMLLEGLGYSVDVKENLTASDMTIELKAFAARPEHRTSDSTFLVLMSHGIR
Oo_CASP1 GADVDIRNMKMLLEGLGYSVDVKENLTASDMTIELKAFAARPEHRTSDSTFLVLMSHGIR
Lv_CASP1 GADVDIRNMKMLLEGLGYSVDVKENLIASDMTIELKAFAARPEHRTSDSTFLVLMSHGIR
Pc_CASP1 GADVDIRSMKMLLEGLGYSVDVKENLTASDMTIELKAFAARPEHRTSDSTFLVLMSHGIQ
Ba_CASP1 GADVDIRNMKMLLEGLGYSVDVKENLTASDMTIELKSFAARPEHRTSDSTFLVLMSHGIR

         241                                                        300
Hs_CASP1 EGICGKKHSEQVPDILQLNAIFNMLNTKNCPSLKDKPKVIIIQACRGDSPGVVWFKDSVG
Bt_CASP1 AGICGKKYSEEVPDILKVDDIFHILNTGNCPALKDKPKVIIIQACRGEKQGMVWVNDSVA
Tt_CASP1 TGVCGKKYSEEVQDVLKVNTIFQILNTWNCPSLKDKPKVIIIQACRGENQGVVWLKDSVE
Oo_CASP1 TGVCGKKYSEEVQDVLKVNTIFQILNTWNCPSLKDKPKVIIIQACRGENQGVVWLKDSVE
Lv_CASP1 AGICGKKYSEEVQDVLKVNTIFQILNTWNCPSLKDKPKVIIIQACRGENQGVVWLKDSVE
Pc_CASP1 TGICGKKYSEEVPDVLKVNTIFQILNTWNCPSLKDKPKVIIIQACRGENQGVVWLKDSVE
Ba_CASP1 TGVCGKKYSEEVPDVLKVNTIFQILNTWNCPSLKDKPKVIIIQACRGENQGVVWLKDSVE

         301                                                        360
Hs_CASP1 VSGNLSLPTTEEFEDDAIKKAHIEKDFIAFCSSTPDNVSWRHPTMGSVFIGRLIEHMQEY
Bt_CASP1 ASGNCSLVAPEDFESDAIKKAHIEKDFIAFCSSTPDNVSWRHPIFGSLFIIKLIETFQEY
Tt_CASP1 ASGNCSLLAPEDFEDDAIKKAHVEKDFIAFCSSTPDNVSWRHPVLGSLFIIKLIEHLQEY
Oo_CASP1 ASGNCSLLAPEDFEDDAIKKAHVEKDFIAFCSSTPDNVSWRHPVLGSLFIIKLIEHLQEY
Lv_CASP1 ASGNCSLLAPEDFEDDAIKKAHVEKDFIAFCSSTPDNVSWRHPVLGSLFIIKLIEHLQEY
Pc_CASP1 ASGKCSLLAPEDFENDAIKKAHVEKDFIAFCSSTPDNVSWRHPILGSLFIIKLIENFQEY
Ba_CASP1 ASGNCSLLAPEDFEDDAIKKAHVEKDFIAFCSSTPDNVSWRHPILGSLFIIKLIENFQEY

         361                                     404
Hs_CASP1 ACSCDVEEIFRKVRFSFEQPDGRAQMPTTERVTLTRCFYLFPGH
Bt_CASP1 AWSCDLEEIFRKVRFSFELPDGKVQMPTAERVTLTRCFYLFPGY
Tt_CASP1 AWFCDLEEIFRKVRFSFELPDGRAQMPTAERVTLTRCFYLFPGY
Oo_CASP1 AWFCDLEEIFRKVRFSFELPDGRAQMPTAERVTLTRCFYLFPGY
Lv_CASP1 AWLCDLEEIFRKVRFSFELPDGKAQMPTAERVTLTRCFYLFPGY
Pc_CASP1 AWFCDLEEIFRKVRFSFELPDGKAQMPTAERVTLTRCFYLFPGY
Ba_CASP1 AWFCDLEEIFRKVRFSFELPDGKAQMPTAERVTLTRCFYLFPGY
```

Suppl. Fig. S12. Amino acid sequence alignment of pyroptosis-related proteins. Continued on the next page.

C

```
                1                                                           60
Hs_NLRP1   MAGGA-WGRLACYLEFLKKEELKEFQLLLANKAHSRSSSGETPAQPEKTSGMEVASYLVA
Dn_NLRP1   MAGGI-KLRLAWYLELLSKEEMAEFQLQLPSKALHEGSSGVTPAETGTANGMEVASRLVA
Ee_NLRP1   MACRAQTQQLVECLEKLSKEELKEFQLQLSKEAPYGGAPGAFPAQPEM-RGVEVASHLVA

                61                                                         120
Hs_NLRP1   QYGEQRAWDLALHTWEQMGLRSLCAQAQEGA--GHSPSFPYSPSEPHLGSPSQPTSTAVL
Dn_NLRP1   QYGEQQAWDLALHTWQQMGLDRLYTQARAEANLGSHTALLTSPSAPGLESPSWPTSTEVL
Ee_NLRP1   QYGEQQAWDLALRTWKKMGLGGLCSQNTEAH------LMSGSPSAPNLESPSWPTSTKVL

                121                                                        180
Hs_NLRP1   MPWIHELPAGCTQGSERRVLRQLPDTSGRRWREISASLLYQALPSSPDHESPSQESPNAP
Dn_NLRP1   SLWGSKYL--CSQDSDPKAPK-----AGCSWQDCSSWLGH--ISSSPSHTSPNQQSPTAP
Ee_NLRP1   -----KVLMPCKVQRRTALSFH----ADRSEHEFAASILP--FTPSPVHNSPSQTSPNAP

                181                                                        240
Hs_NLRP1   TSTAVLGSWGSPPQPSLAPREQEAPGTQWPLDETSGIYYTEIREREREKSEKGRPPWAAV
Dn_NLRP1   MSTAVLGGWVPPPQPSPDPREQAASQAKGLLTEGS--------HKENRKTYKNP------
Ee_NLRP1   TSTGVLGAWEPPPQPSTKPREQEDPRTGQLLGESS-----KDCLTENREYYQNQ------

                241                                                        300
Hs_NLRP1   VGTPPQAHTSLQPHHHPWEPSVRESLCSTWPWKNEDFNQKFTQLLLLQRPHPRSQDPLVK
Dn_NLRP1   --IRGRSHFTLGQ--------------KSQSWKKEDFHQKFTELILLHRTGPQVQEFLFT
Ee_NLRP1   ---KGEKTCPTGDQ-------------NSQSWKNEVLYQKFVQLLLLERPPSKGHVPLMT

                301                                                        360
Hs_NLRP1   RSWPDYVEENRGHLIEIRDLFGPGLDTQE-PRIVILQGAAGIGKSTLARQVKEAWGRGQL
Dn_NLRP1   GSWPQEAKE-QGHLIEVQDLFGPGLGTQEGPRTVLLMGAAGIGKSTLARQVRRAWEDGQL
Ee_NLRP1   DHGMMVEQK---RLIEVQDLFGPDLGTQEKPHTVILHGAGGIGKSTLARQVRGAWEQGQL

                361                                                        420
Hs_NLRP1   YGDRFQHVFYFSCRELAQSKVVSLAELIGKDGTATPAPIRQILSRPERLLFILDGVDEPG
Dn_NLRP1   YRDRFQHVFYLNCRELAQTKVVSLAELMA-DQAAPLAPIGQILSQPTQLLLILDGFDEPG
Ee_NLRP1   YRDRFRHVFYFNCKELVPSNMVSLVELLAKDWPHPLAPIRQILSQPGQLLLILDGLDEPE

                421                                                        480
Hs_NLRP1   WVLQEPSSELCLHWSQPQPADALLGSLLGKTILPEASFLITARTTALQNLIPSLEQARWV
Dn_NLRP1   WIFKKHRSELCFLWSQQQPVHILLSSLLRKTLLPEASLLITAQTTSLLELLFFVEQLHWV
Ee_NLRP1   WVFERQTPEMCLHWSQQQPVHMLLNSLLEKTTLPEASLLITARRTALGKLLPSLGQARCV

                481                                                        540
Hs_NLRP1   EVLGFSESSRKEYFYRYFTDERQAIRAFRLVKSNKELWALCLVPWVSWLACTCLMQQMKR
Dn_NLRP1   EVLGFSEAGRKEYFYNYFTDDSQATRAFSLVESNQALLTMCLVPWVSRLVCTCLKQQMER
Ee_NLRP1   EVLGFSEAGRKDFFYKYFPDESQAVMAFSSIASNHALLILCLRPWVSWLVSTCLKLQMEQ

                541                                                        600
Hs_NLRP1   KEKLTLTSKTTTTLCLHYLAQALQAQPLGPQLRDLCSLAAEGIWQKKTLFSPDDLRKHGL
Dn_NLRP1   GEELPLTSQTTTALCLHYLSQAFPAQPLRAQLKSLCSLAAKSTEQGKALFSLCYLKKQGF
Ee_NLRP1   GEPPALTAQTTTDLCLHYLSQALRAQLLGTHLRALCCLATEGIWQEKTLFSGKELRKYGL
```

Suppl. Fig. S12. Amino acid sequence alignment of pyroptosis-related proteins. Continued on the next page.

```
          601                                                         660
Hs_NLRP1  DGAIISTFLKMGILQEHPIPLSYSFIHLCFQEFFAAMSYVLEDEKGRGKHSNCIIDLEKT
Dn_NLRP1  SKAVISTFVKMGVFQK-PTFLSFSFAHSGLQEFFAAIFYALGDEE-RSHYPKSTHDVRKL
Ee_NLRP1  DGAVIFTLLRTGVLQKHPTLLRYHFRHLFFQEFLAAVFYALEEEEEGGKL-DSIKSVKKL

          661                                                         720
Hs_NLRP1  LEAYGIHGLFGASTTRFLLGLLSDEGEREMENIFHCRLSQGRN--LMQWVPSLQ----LL
Dn_NLRP1  LRVCGRQVVFGTLTTRFLFGLAGEHGARELENIFNLKPSGELRGELLRWVETELRCGGFS
Ee_NLRP1  QEAYEMYGMFGAPVTHFLFGLLSERGTRELESIFSCQLSKKAKWELLQWVELEVWHKHSW

          721                                                         780
Hs_NLRP1  LQPHSLESLHCLYETRNKTFLTQVMAHFEEMGMCVETDMELLVCTFCIKFSRHVKKLQLI
Dn_NLRP1  KQPYRLELLLCLYEIQDEEFLTQVMAYFQEARVWIQTDVDLLVFTFCLQFCRNVKRLQL-
Ee_NLRP1  PQLYSWAVLHCLYEIQDEAFLTRAMAHFQGARMWVHTDIELLVFTFCLKFCCNVKSLQVN

          781                                                         840
Hs_NLRP1  EGRQHRSTWSPTMVVLFRWVPVTDAYWQILFSVLKVTRNLKELDLSGNSLSHSAVKSLCK
Dn_NLRP1  -SGQHGPAWRAHSVVLCMEVPITDARWQILFSILGVAGSLRELDLSGNSLSHSAVQGLCK
Ee_NLRP1  VNSACGQALKSPSVVLFRSALVTDACWQDLFAILGVTQSLQELDLSGNPLSCSVVQSLCE

          841                                                         900
Hs_NLRP1  TLRRPRCLLETLRLAGCGLTAEDCKDLAFGLRANQTLTELDLSFNVLTDAGAKHLCQRLR
Dn_NLRP1  ALRHPQCHLETLRLVGCGLT----------------------------------------
Ee_NLRP1  ALRHPHCHLEILRLVSCGLT----------------------------------------

          901                                                         960
Hs_NLRP1  QPSCKLQRLQLVSCGLTSDCCQDLASVLSASPSLKELDLQQNNLDDVGVRLLCEGLRHPA
Dn_NLRP1  -----------------SSCCADLASALGASPSLRELDLQQNDLDAAGVGLLCEGLRHPA
Ee_NLRP1  -----------------SDCCQALASVLSTTPTLTELDLQQNDVGSQGVRLLCKGLHHPN

          961                                                        1020
Hs_NLRP1  CKLIRLGLDQTTLSDEMRQELRALEQEKPQLLIFSRRKPSVMTPTEGLDTGEMSNSTSSL
Dn_NLRP1  CPLKLLQLGQIPWSDKVSQELRALEEEKPQLLVTSRWKQSVAMAKEDPDWGETSSETSSL
Ee_NLRP1  CQLKRLWLDQIHLNEEVSVELRALQVVKPQLLVSSRWEQRTVNPTEGQDGGEMSIE-SSL

          1021                                                       1080
Hs_NLRP1  KRQRLGSERAASHVAQANLKLL--DVSKIFPIAEIAEESSPEVVPVELLCVPSPASQGDL
Dn_NLRP1  KRQR----------------------------SESAEEHSPQATLAEPRWLAGPSLPEDL
Ee_NLRP1  KRQRTESDTALEKARWRDPWNICQENLELTTALPVSGESSPQAVQGKSACLSSPALLEDL

          1081                                                       1140
Hs_NLRP1  HTKPLGTDDDFWGPTGPVATEVVDKEKNLYRVHFPVAGSYRWPNTGLCFVMREAVTVEIE
Dn_NLRP1  LTEPLATEDDFQGPTGLVATQVVDEEKGQYRVHFPMAGTYRWPSTRLCFVVRWAVTLEIE
Ee_NLRP1  HMEPLGIAENFWGPTGPVATRMIDKERSLYRVHFPAAGSYHWPNTGLRFIVKGEVTIEIE

          1141                                                       1200
Hs_NLRP1  FCVWDQFLGEINPQHSWMVAGPLLDIKAEPGAVEAVHLPHFVALQGGHVDTSLFQMAHFK
Dn_NLRP1  FCAWSQFLDGTSLQHGWMVAGPLFDIKAEPGAVAAVHLPHFVNLEEGNVDISLFQVAHIK
Ee_NLRP1  ICNWGKFLNESASQHNWMVAGPLFDIKAEPGAVAAVYLPHFVALQGGHVDTSMFRVAHFK
```

Suppl. Fig. S12. Amino acid sequence alignment of pyroptosis-related proteins. Continued on the next page.

```
         1201                                                        1260
Hs_NLRP1 EEGMLLEKPARVELHHIVLENPSFSPLGVLLKMIHNALRFIPVTSVVLLYHRVHPEEVTF
Dn_NLRP1 EEGTLLEKPARVEPEHAVLESPSFSPMGVLLRVIHAALR-IPITSIVLLYHRRHPEEVVF
Ee_NLRP1 EEGMLLEMPARVEPSSTVLENPSFSPMGVLLRMVHAALRFIPITSTVLLYHHLLPEEVTF

         1261                                                        1320
Hs_NLRP1 HLYLIPSDCSIRKAIDDLEMKFQFVRIHKPPPLTPLYMGCRYTVSGSGSGMLEILPKELE
Dn_NLRP1 HLYLVPSDCSIRKAIDDEEKMSQFVQLRKPPPLTPLYMGSRYVVSGSEN--LEMIPKELE
Ee_NLRP1 HLYLIPSDCSIQKAIDDEEKKFEFVRLNKPPPLTPLYIGSRYVVSGSNN--LEMIPEELE

         1321                                                        1380
Hs_NLRP1 LCYRSPGEDQLFSEFYVGHLGSGIRLQVKDKKDETLVWEALVKPGDLMPATTLIPPARIA
Dn_NLRP1 LCYRSPGQPQLFSEFYVGHLGSGIRLQVKDKKCGTMVWEALVKPGDLRTAVTPLPPSPKA
Ee_NLRP1 LCYRNPGQSQLFSEFYVDHLGSGIRLEMKDKKDGIVVWKALVKQ----------------

         1381                                                        1440
Hs_NLRP1 VPSPLDAPQLLHFVDQYREQLIARVTSVEVVLDKLHGQVLSQEQYERVLAENTRPSQMRK
Dn_NLRP1 SP-PLPYAPALHFVDRHREQLVARVTSVEPILDKLHGWVLSEEQYERVRAEATTLDQMRK
Ee_NLRP1 -----EVQDRLHFVDWYREQLVARVTLVDPVLDKMHRHVLSEEQCESVRAEATKPAQMRK

         1441                                           1492
Hs_NLRP1 LFSLSQSWDRKCKDGLYQALKETHPHLIMELWEKGSKK----GLLPLSS---
Dn_NLRP1 LFSFSRSWNLACKDRLYQALEEIHPHLTVELRKCDSHR-DQEGLLTNSAAEV
Ee_NLRP1 LFSFSRSWDWARKNRFYQALRETHPYLIMELWEKWGSIGNQEVS-------
```

D

```
          1                                                           60
Hs_NLRP10 MAMAKARKPREALLWALSDLEENDFKKLKFYLRDMTLSEGQPPLARGELEGLIPVDLAEL
Ee_NLRP10 --MTLSRGPEETLLFALNDLEDESFKTLKFHLRIMTPRDGHQQLTRGELQDLKRVELASR

          61                                                         120
Hs_NLRP10 LISKYGEKEAVKVVLKGLKVMNLLELVDQLSHICLHDYREVYREHVRCLEEWQEAGVNGR
Ee_NLRP10 LIQIYGAYEAVKIVLESLKKMNLLELVGQLSLVCLNDYREKYREHVRCLEEKQEVGINGS

          121                                                        180
Hs_NLRP10 YNQVLLVAKPSSESPESLACPFPEQELESVTVEALFDSGEKPSLAPSLVVLQGSAGTGKT
Ee_NLRP10 YNHLLLVSGSSSGSPEVSTCPTLEQEL--VLVEDLFGSGERHHT--STVVLQGLAGTGKT

          181                                                        240
Hs_NLRP10 TLARKMVLDWATGTLYPGRFDYVFYVSCKEVVLLLESKLEQLLFWCCGDNQAPVTEILRQ
Ee_NLRP10 TLARKMVLDWARGTLFMGRFDYVFYVSCREVVLLRGCKLEQLLCWCCGDNQAPVEEILRH

          241                                                        300
Hs_NLRP10 PERLLFILDGFDELQRPFEEKLKKRGLSPKESLLHLLIRRHTLPTCSLLITTRPLALRNL
Ee_NLRP10 PERLLFILDGFDELQKSFAEQLMKSSLSSKECELHLLIKRTILCKSSLFITIRPLALQNL

          301                                                        360
Hs_NLRP10 EPLLKQARHVHILGFSEEERARYFSSYFTDEKQADRAFDIVQKNDILYKACQVPGICWVV
Ee_NLRP10 RGLLKKPRYVHILGFSEEEKRRYFSFYFTDEEQARNAFDIVQRYKVLHKECQVPGICWMV

          361                                                        420
Hs_NLRP10 CSWLQGQMERGKVVLETPRNSTDIFMAYVSTFLPPDDDGGCSELSRHRVLRSLCSLAAEG
Ee_NLRP10 CSWIKRQMERGREISNIFRNSTDIYMAYVSTFLPPNDDGDGLNLPRYSALTGLCTLATEG
```

Suppl. Fig. S12. Amino acid sequence alignment of pyroptosis-related proteins. Continued on the next page.

```
           421                                                        480
Hs_NLRP10  IQHQRFLFEEAELRKHNLDGPRLAAFLSSNDYQLGLAIKKFYSFRHISFQDFFHAMSYLV
Ee_NLRP10  IQKQQFLFEEADLRKHNLDGPWLADFLSSIDYHEGFAVKRFYSFRHISFQEFFHAISYLV

         481                                                        540
Hs_NLRP10  KEDQSRLGKESRREVQRLLEVKEQEGNDEMTLTMQFLLDISKK-DSFSNLELKFCFRISP
Ee_NLRP10  KEEHNQLGQESHSELKRLLDTK----SDDRTLDVRFLLEILKNGETASILELKLCFKISS

           541                                                        600
Hs_NLRP10  CLAQDLKHFKEQMESMKHNRTWDLEFSLYEAKIKNLVKGIQMNNVSFKIKHSNEKKSQSQ
Ee_NLRP10  TITQNLKDLKKQLESMRHSRPWNLEFSLNGSKIKDLKNSVQMSEASFSWKNKNVSQGSKG

           601                                                        660
Hs_NLRP10  NLFSVKSSLSHGPKEEQKCPSVHGQKEGKDNIAGTQKEASTGKGRGT-EETPKNTYI---
Ee_NLRP10  RSFSVKTSLRNEQEEEEKSHPMD-----KENEAGTQNETSNGKDRDTRDKRVMSSWVEKC

           661                                   699
Hs_NLRP10  ---------------------------------------
Ee_NLRP10  VNGNNRDEVAEECWVTETGTLGKARQSKKDRSNKTDNGF
```

E

```
           1                                                           60
Hs_PYDC1   MGTKREAILKVLENLTPEELKKFKMKLGTVPLREGFERIPRGALGQLDIVDLTDKLVASY
Dn_PYDC1   MGKKRDAILEALENLTSDELKKFKLKLGVVPLREGFRNIPRGALGPLDPVDLTDKLVSFY

           61                           89
Hs_PYDC1   YEDYAAELVVAVLRDMRMLEEAARLQRAA
Dn_PYDC1   REDYGAELAATVLLDMGMQEEASRLQAVA
```

Suppl. Fig. S12. Amino acid sequence alignment of pyroptosis-related proteins. The amino acid sequences of pyroptosis-related proteins from human (Hs, *Homo sapiens*), cattle (Bt, *Bos taurus*) and cetaceans (Tt, *Tursiops truncatus*; Oo, *Orcinus orca*; Lv, *Lipotes vexillifer*; Pc, *Physeter catodon*; Ba, *Balaenoptera acutorostrata scammoni*) were aligned with the Multalin algorithm using default parameters (**A**, **B**). For proteins not conserved in cattle and cetaceans, orthologous sequences were identified in two basal placental mammalian species, the armadillo (Dn, *Dasypus novemcinctus*) and the elphant shrew (Ee, *Elephantulus edwardii*), and used for alignments (**C**-**E**). Amino acid residues conserved in all species are shown in red color, and amino acid residues conserved in more than 50% of all species are marked in blue. GenBank accession numbers of proteins: Ba_CASP1, XP_007191299; Ba_PYCARD, XP_007107499; Bt_CASP1, XP_002692967; Bt_PYCARD, NP_777155; Dn_NLRP1, XP_004483564; Dn_PYDC1, XP_004479485; Ee_NLRP1, XP_006899866; Ee_NLRP10, XP_006885666; Hs_CASP1, NP_150634; Hs_NLRP1, NP_127497; Hs_NLRP10, NP_789791; Hs_PYCARD, NP_037390; Hs_PYDC1, NP_690865; Lv_CASP1, XP_007461943; Lv_PYCARD, XP_007452741; Oo_CASP1, XP_004282232; Oo_PYCARD, XP_004268772; Pc_PYCARD, XP_007107499; Pc_CASP1, XP_007124637; Tt_CASP1, XP_004319499. The amino acid sequence of the *T. truncatus* PYCARD protein was obtained by translation of the coding sequence of the *PYCARD* gene identified in Tursiops truncatus Contig537899, whole genome shotgun sequence (Accession number ABRN02523200, nucleotide positions: 3141-3414 (exon 1), 3683-3740 (exon 2), 4104-4360 (exon 3)).

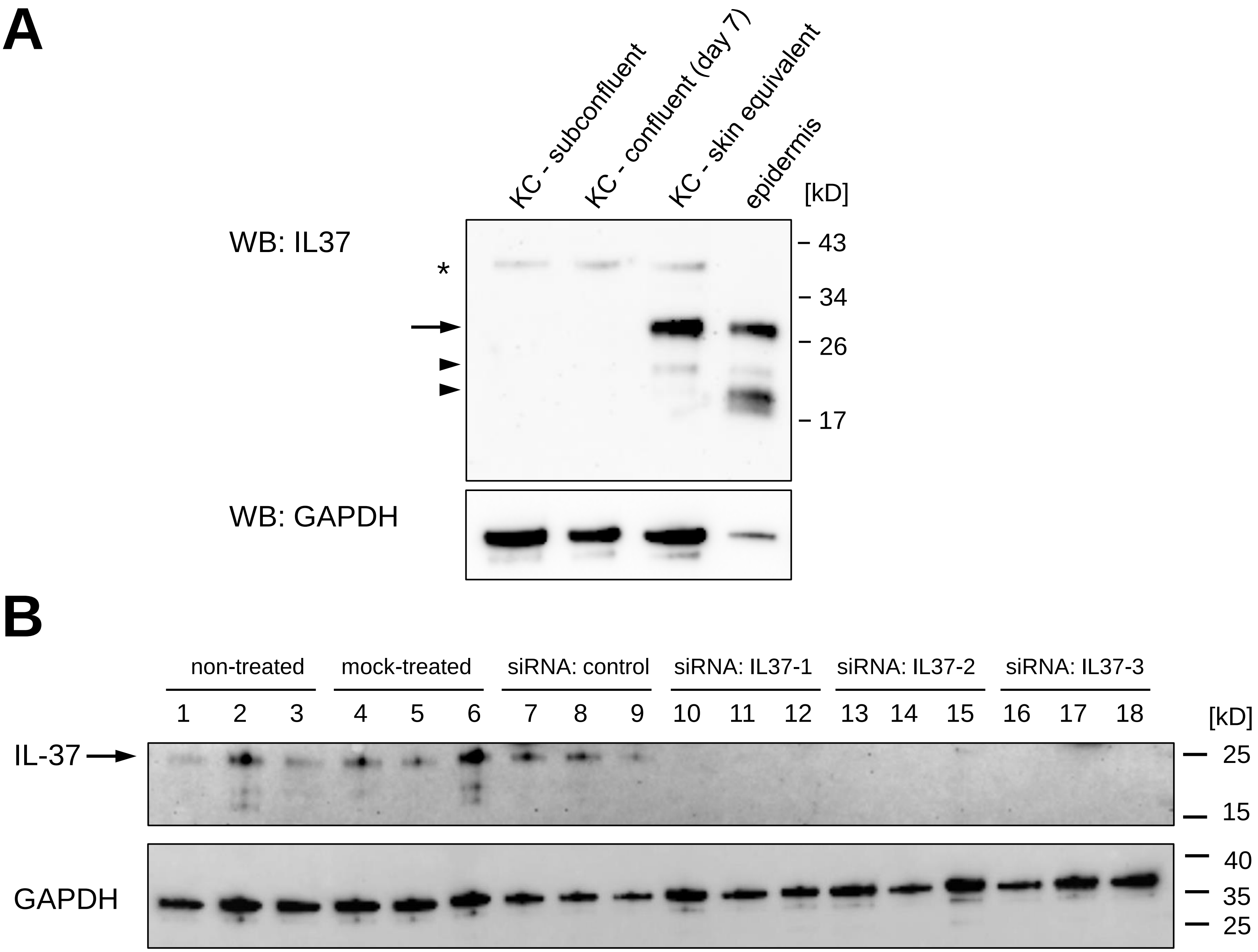

Suppl. Fig. S13. Western blot analysis of IL-37. (**A**) Western blot (WB) analysis of IL-37 and GAPDH in cultured keratinocytes and human epidermis. The main band (arrow) corresponds to the siRNA-sensitive band in panel **B**. *, presumably unspecific band; arrowheads, bands of unknown identity possibly corresponding to IL37 isoforms or proteolytic fragments. Note that the intensity of the main band (indicated by the arrow) correlates with IL37 mRNA abundance (Fig. 1E; Suppl. Fig. S11). d7, day 7 of confluence. Antibodies: anti-IL-37 (HPA054371, Sigma-Aldrich), anti-GAPDH (5G4 MAb 6C5, HyTest Ltd.). (**B**) IL37-knockdown experiment and Western blot analysis of IL-37 (upper panel) in skin equivalents. Keratinocytes were subjected to different treatments, as indicated, and subsequently used to make 3 skin equivalents per treatment. Protein lysates of the epidermal compartment of each skin equivalent were analysed by WB for IL-37 and GAPDH. The positions of size markers (kD, kilo-Dalton) are indicated on the right. Only the expected size range of IL-37 proteins is shown here whereas an image of the full molecular weight range is shown in Suppl. Fig. S14G. The protein preparation and western blot protocols were as follows: Proteins were extracted from cells by sonication and from tissues with the Precellys homogenizer (VWR) in a buffer containing 2% SDS. Protein concentrations were measured with the Micro BCA Protein Assay Kit (Thermo Fisher Scientific). 30 µg protein per lane were electrophoresed through an ExcelGel SDS gradient 8-18% polyacrylamide gene (GE Healthcare Life Sciences) and blotted onto a nitrocellulose membrane. The membranes were incubated with affinity isolated polyclonal rabbit anti-IL37 (1:200, HPA054371, lot R72671, Sigma-Aldrich) at 4°C over night. After washing the membrane was incubated with a second step antibody coupled to horse-raddish peroxidase for 1 h at room temperature. Finally, the immunolabeled proteins were visualized using the enhanced chemoluminescence system (SuperSignal West Dura Extended Duration Substrate, 34075, lot RD229335, Thermo Fisher Scientific). The membrane was re-probed with mouse monoclonal anti-GAPDH (1:2000, 5G4 MAb 6C5, lot 16/06-G4-C5, HyTest Ltd, Turku, Finland).

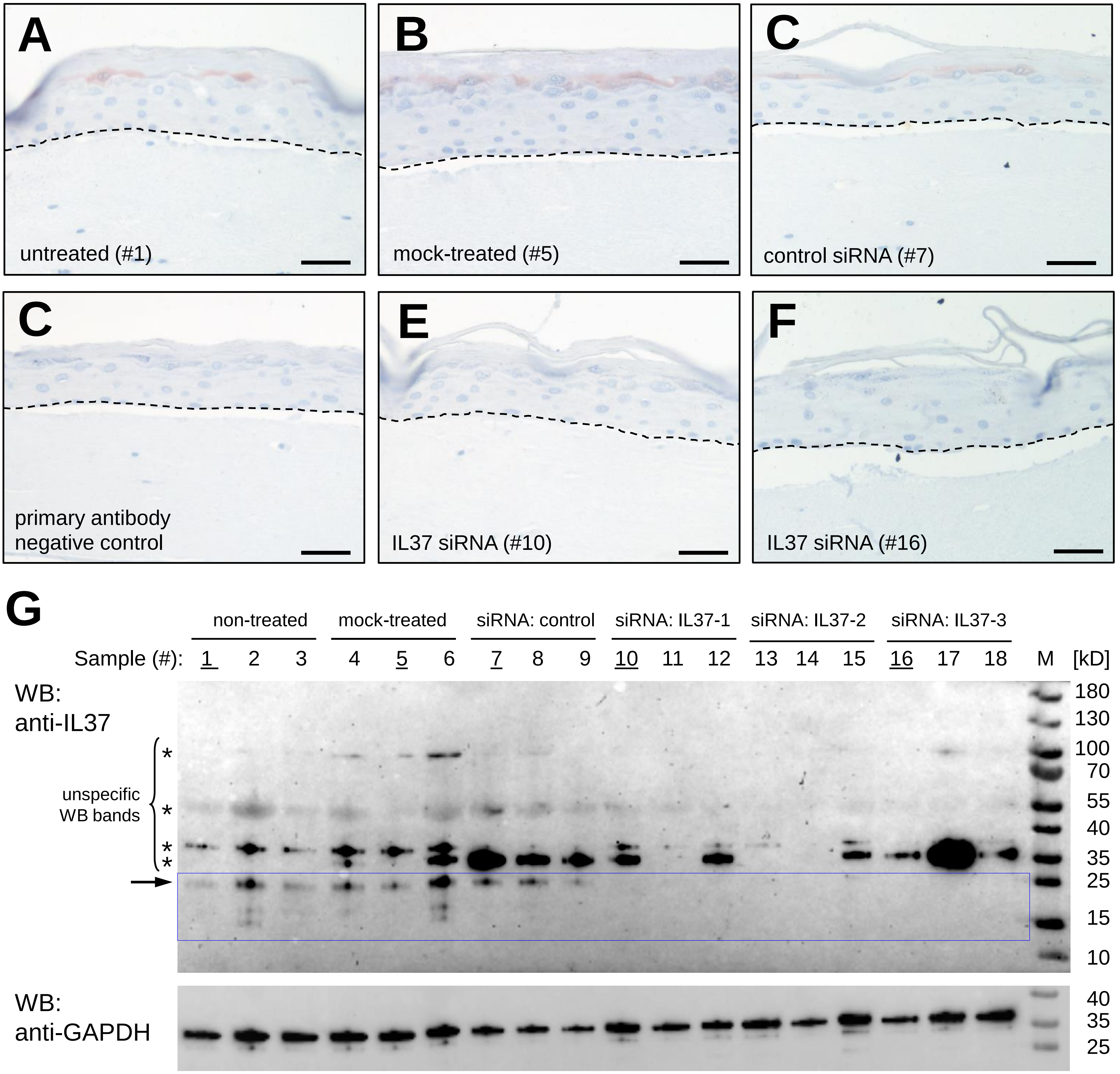

Suppl. Fig. S14. Comparison of IL-37 immunohistochemical staining and IL-37 western blot analysis of skin equivalents containing keratinocytes either transfected with IL37-specific siRNA or subjected to control treatments. (**A-C**, **E-F**) Immunohistochemical staining of IL-37 (red) in skin equivalents subjected to the indicated treatments. A negative control experiment with an unrelated primary antibody (on the control siRNA-treated skin equivalent sample #7) (**D**) did not yield a staining. The numbers (#) indicate experiments analyzed by western blot, as depicted in panel **G**. Scale bars, 50 µm (**A-F**). (**G**) Western blot (WB) analysis of IL-37 in skin equivalents containing keratinocytes subjected to different treatments, as indicated. Three skin equivalents were made per treatment, and the proteins were prepared from the epidermal compartment of each skin equivalent. The protocol of the western blot for IL-37 and GAPDH is described in the legend of Suppl. Fig. S13B. Bands over the complete molecular weight range are shown. The part of the western blot scan shown in Suppl. Fig. S13B is indicated by a blue frame. The GAPDH western blot (same image as in Suppl. Fig. S13B) is shown to confirm equal loading of lanes. A marker (M) of protein sizes (kD, kilo-Dalton) is included on the right. Bands, that are larger than any known isoform of IL-37 and that are not specifically suppressed by IL37-siRNA, are indicated by asterisks on the left. Of note, different secondary antibodies were used in western blot and immunhistochemical analyses so that cross-reactivities in the western blot do not correlate with cross-reactivities in immunohistochemistry. For example, skin equivalents #10 and #16 (both containing keratinocytes in which IL37 expression was knocked down) did not show an IL-37 immunohistochemistry signal (**E**, **F**) although unspecific bands were present in the IL-37 western blot (**G**). Instead, the immunohistochemical signals correlated with the presence of the IL-37 WB band indicated by the arrow.

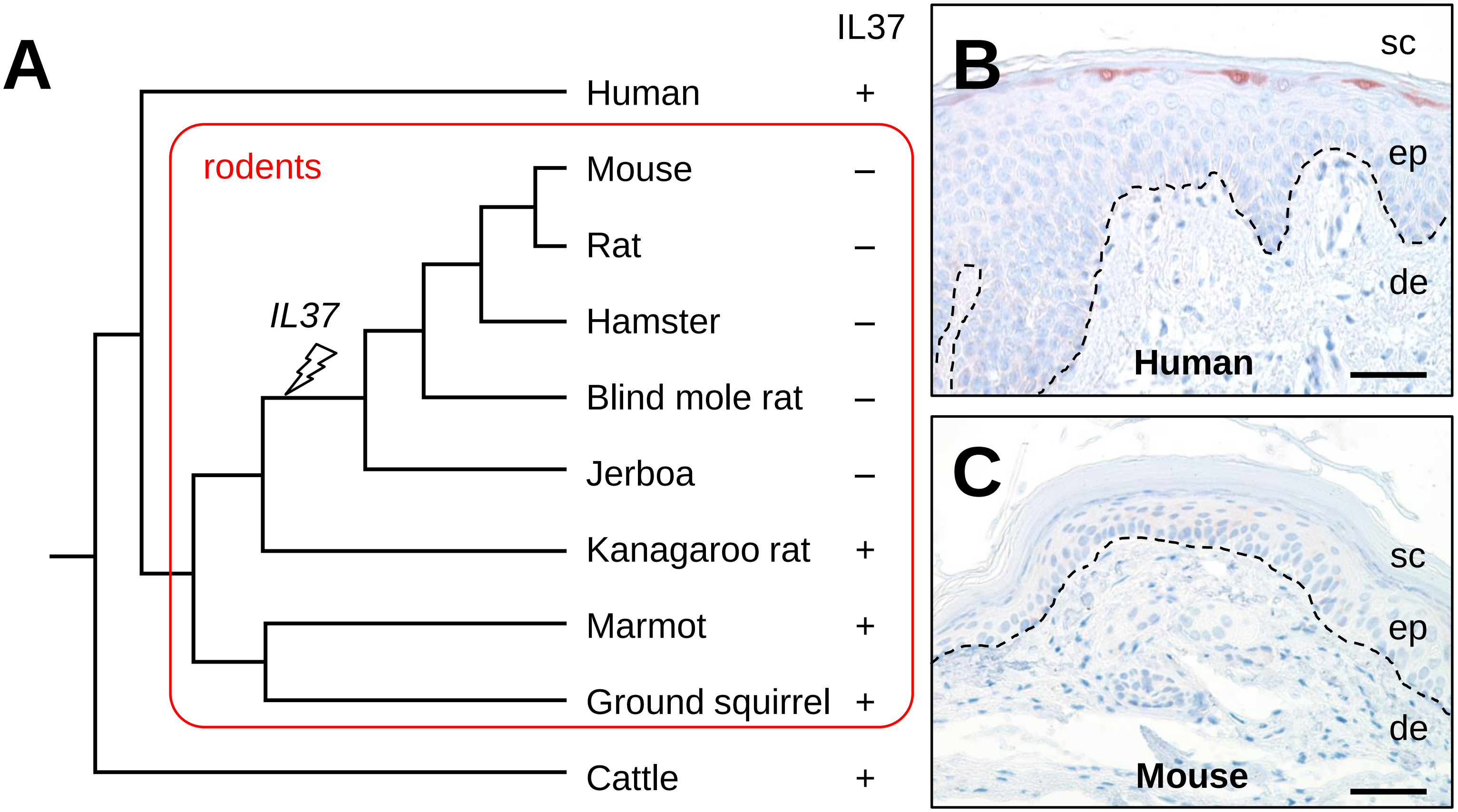

Suppl. Fig. S15. The *IL37* gene has been inactivated in a subclade of rodents. (**A**) The presence and absence of functional gene orthologs (indicated by + and –, respectively) of IL37 was mapped onto a phylogenetic tree of rodents using human and cattle as outgroups. The strike symbol indicates the putative gene inactivation event leading to the distribution of the functional IL37 genes in the species investigated. The *IL37* gene is functional (no inactivating mutations) in the thirteen-lined ground squirrel (*Ictidomys tridecemlineatus*) (NW_004936783), the marmot (*Marmota marmota marmota*) (NW_015351273), and the kangaroo rat (*Dipodomys ordii*) (NW_012267263.1, second coding exon absent in the GenBank gen prediction but identified at nucleotides 41682-41744 on the reverse strand). The *IL37* gene orthologs carries inactivating mutations in the jerboa (*Jaculus jaculus*) (NW_004504449). No *IL37* ortholog could be identified in IL1F gene cluster of the blind mole rat (*Nannospalax galili*) (NW_008344179), hamster (*Mesocricetus auratus*) (NW_004801618), rat (*Rattus norvegicus*) (NC_005102) and mouse (*Mus musculus*) (NC_000068). Based on the estimates of the indicated phylogenetic divergence times at http://www.timetree.org/, the time of *IL37* inactivation is estimated at 70-55 million years ago. (**B**) Immunohistochemistry with the anti-IL37 antibody (Materials and Methods) detected IL-37 in the granular layer of human epidermis which is in agreement with the data shown in Fig. 1A. By contrast, no immunostaining with anti-IL37 was obtained in the epidermis on the sole (**C**) and on the back (not shown) of mice. Epidermal-dermal junctions are indicated by discontinous lines. Scale bars, 50 µm. de, dermis; ep, epidermis; sc, stratum corneum.

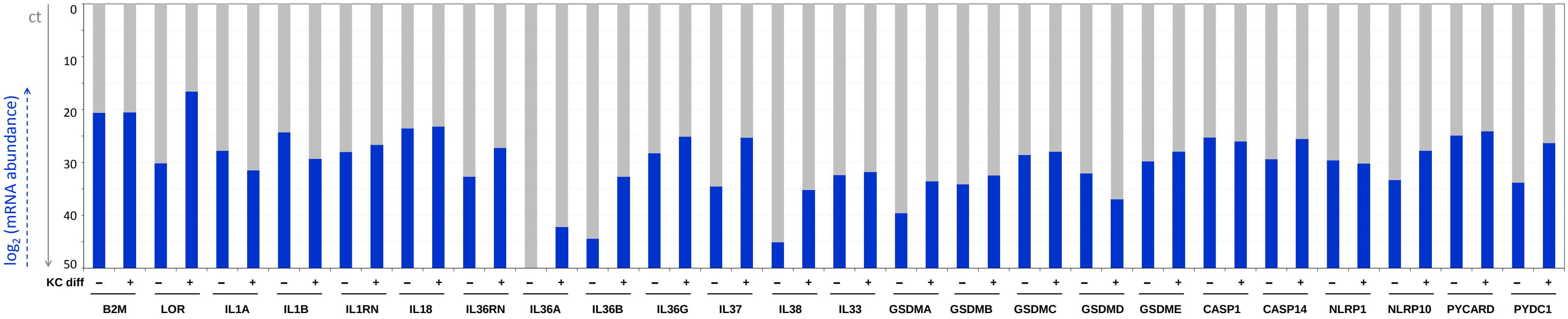

Suppl. Fig. S16. Comparison of real time PCR cycle threshold (ct) values. Mean ct values of the results displayed in Figures 1E, 2E and 3A are shown (n=3). The ct values are represented by grey bars with values on the vertical axis oriented from top down. ct values correlate negatively with the logarithm of the mRNA abundance, indicate by blue bars. Note that this estimate of mRNA abundance is simplified by assuming a PCR amplification efficiency equal to 2 for all PCRs. Keratinocytes (KC) in subconfluent cultures that do not differentiate (–) and keratinocytes differentiated in skin equivalent cultures (+) were investigated.

Supplementary Table S1. IL1 family genes of cetaceans.

Species	Gene	Accession nr. of genomic sequence	CDS start	CDS end	Protein encoded	Location of mutation	Information about mutations
Dolphin	IL1A	NW_017842906.1	596186	605799	**yes**	n.a.	
	IL1B	NW_017842906.1	544552	550278	**yes**	n.a.	
	IL1RN	NW_017842906.1	347448	342165	**yes**	n.a.	
	IL18	NW_017843657.1	18145567	18154531	**yes**	n.a.	
	IL33	NW_017843160.1	49586174	49598001	**yes**	n.a.	
	IL36A	n.a.	n.a.	n.a.	no	n.a.	gene absent
	IL36B	NW_017842906.1	430519	426333	no	426815	frameshift leads to a premature stop codon
	IL36G	ABRN02501551.1	5682	885	**yes**	n.a.	
	IL36RN	NW_017842906.1	402114	398959	**yes**	n.a.	
	IL37	n.a.	n.a.	n.a.	no	n.a.	gene absent
	IL37 like	n.a.	n.a.	n.a.	no	n.a.	gene absent
	IL38	NW_017842906.1	388896	385974	no	386084	premature stop codon
Orca	IL1A	NW_004438498.1	11759392	11749208	**yes**	n.a.	
	IL1B	NW_004438674.1	1034136	1040491	**yes**	n.a.	
	IL1RN	NW_004438674.1	788497	783194	**yes**	n.a.	
	IL18	NW_004438469.1	14051933	14060911	**yes**	n.a.	
	IL33	NW_004438521.1	7792463	7811089	**yes**	n.a.	
	IL36A	n.a.	n.a.	n.a.	no	n.a.	gene absent
	IL36B	NW_004438674.1	871114	866641	no	866720	frameshift leads to a premature stop codon
	IL36G	NW_004438674.1	>883294	879013	**yes**	n.a.	
	IL36RN	NW_004438674.1	842029	838845	**yes**	n.a.	
	IL37	n.a.	n.a.	n.a.	no	n.a.	gene absent
	IL37 like	NW_004438674.1	914403	919307	no	914445	premature stop codon
	IL38	NW_004438674.1	835375	831843	no	831951	premature stop codon
Baiji	IL1A	NW_006770093.1	288947	278299	**yes**	n.a.	
	IL1B	NW_006770093.1	345015	337802	**yes**	n.a.	
	IL1RN	NW_006770093.1	587641	592941	**yes**	n.a.	
	IL18	NW_006791778.1	802739	811719	**yes**	n.a.	
	IL33	NW_006775781.1	496315	508936	**yes?**	n.a.	ancestral start codon at 496303 is mutated
	IL36A	n.a.	n.a.	n.a.	no	n.a.	gene absent
	IL36B	NW_006770093.1	497264	501011	no	499836	frameshift
	IL36G	NW_006770093.1	<484119	488409	**yes**	n.a.	
	IL36RN	NW_006770093.1	532259	535433	no	535308	frameshift leads to a premature stop codon
	IL37	NW_006770093.1	426020	431706	no	427561	premature stop codon
	IL37 like	n.a.	n.a.	n.a.	no	n.a.	gene absent
	IL38	NW_006770093.1	541522	543077	no	542967	premature stop codon
Sperm whale	IL1A	NW_006716736.1	84925	77130	**yes**	n.a.	
	IL1B	NW_006716736.1	134664	128920	**yes**	n.a.	
	IL1RN	NW_006714308.1	67246	72392	**yes**	n.a.	
	IL18	NW_006724284.1	>610408	591150	**yes**	n.a.	N-terminus encoded by accession: AWZP01093886.1, nucl. 600 - 520
	IL33	NW_006714296.1	312802	300952	**yes**	n.a.	
	IL36A	NW_006719196.1	33897	36453	no	36265	stop codon mutated; other mutations
	IL36B	NW_006719196.1	39491	45970	no	44839	frameshift
	IL36G	NW_006719196.1	7455	12277	**yes**	n.a.	
	IL36RN	NW_006714308.1	9814	12769	**yes**	n.a.	
	IL37	n.a.	n.a.	n.a.	no	n.a.	gene absent
	IL37 like	NW_006719865.1	33974	22490	no	33917	premature stop codon; start codon mutated; other mutations
	IL38	NW_006714308.1	21027	23562	no	23374	premature stop codon
Minke whale	IL1A	NW_006734456.1	7399880	7389979	**yes**	n.a.	
	IL1B	NW_006734456.1	7456637	7450758	**yes**	n.a.	
	IL1RN	NW_006734456.1	7763274	7768211	**yes**	n.a.	
	IL18	NW_006725887.1	<33541	42270	**yes**	n.a.	gene sequence is incomplete
	IL33	NW_006727131.1	169416	157759	**yes**	n.a.	
	IL36A	NW_006734456.1	7635814	7642785	no	7640630	frameshift leads to a premature stop codon; other muations
	IL36B	NW_006734456.1	7655573	7659992	no?		premature stop leads to loss of 7 aa at C-terminus; uncertain effect on function
	IL36G	NW_006734456.1	<7606282	7618964	no	7618753	ancestral splice site mutated
	IL36RN	NW_006734456.1	7702184	7705162	**yes**	n.a.	
	IL37	NW_006734456.1	7561246	7567471	no	7566975	new splice site - stop codon
	IL37 like	n.a.	n.a.	n.a.	no	n.a.	gene absent
	IL38	NW_006734456.1	7715403	7718660	no	7718550	premature stop codon

Notes: For genes not encoding proteins due to inactivating mutations, "CDS start" and "CDS end" refer to sites homologous to ancestral start and stop codons, respectively.
The location of only one mutation is indicated but additional mutations are present in some of these genes.
aa, amino acid residues; CDS, coding sequence; IL, interleukin, n.a., not applicable.

Supplementary Table S2. Gasdermin genes of cetaceans.

Species	Gene	Accession nr. of genomic sequence	CDS start	CDS end	Protein encoded	Location of mutation	Information about mutations
Dolphin	GSDMA	n.a.	n.a.	n.a.	no	n.a.	gene absent
	GSDMB	NW_017843467.1	151694	141669	no	151520	premature stop codon
	GSDMC	NW_017842982.1	8272952	8268139	no	8268216	premature stop codon and other mutations
	GSDMD	NW_004197581.1	73339	78981	**yes**	n.a.	
	GSDME	NW_017842131.1	47041326	47093619	**yes**	n.a.	
Orca	GSDMA	n.a.	n.a.	n.a.	no	n.a.	gene absent
	GSDMB	NW_004438583.1	4556584	4545444	no	4556410	premature stop codon
	GSDMC	NW_004438426.1	10794280	10789465	no	10789542	premature stop codon and other mutations
	GSDMD	NW_004438426.1	1024476	1021046	**yes**	n.a.	
	GSDME	NW_004438428.1	15535076	15471200	**yes**	n.a.	
Baiji	GSDMA	n.a.	n.a.	n.a.	no	n.a.	gene absent
	GSDMB	NW_006791954.1	4445876	4434668	no	4445702	premature stop codon
	GSDMC	NW_006773072.1	467313	472281	no	472205	premature stop codon and other mutations
	GSDMD	NW_006791731.1	1087131	1090600	**yes**	n.a.	
	GSDME	NW_006792921.1	745976	683477	**yes**	n.a.	
Sperm whale	GSDMA	n.a.	n.a.	n.a.	no	n.a.	gene absent
	GSDMB	NW_006714299.1	612437	623330	no	612611	premature stop codon
	GSDMC	NW_006712916.1	612376	618594	no	617707	mutated splice site
	GSDMD	NW_006714406.1	155309	160174	**yes**	n.a.	
	GSDME	NW_006714383.1	209639	158867	no	209607	frameshift mutation in exon 1
Minke whale	GSDMA	NW_006726421.1	1671121	1696569	no	1691099	premature stop codon and other mutations
	GSDMB	NW_006726421.1	1640314	1628611	no	1639725	premature stop codon
	GSDMC	n.a.	n.a.	n.a.	no	n.a.	gene absent
	GSDMD	NW_006725399.1	2186942	2183262	**yes?**	2183390	gene sequence is incomplete, elongated C-terminus due to mutation of ancestral stop codon
	GSDME	NW_006725732.1	2126251	2054990	no	2072494	stop codons in exon 5

Notes: For genes not encoding proteins due to inactivating mutations, "CDS start" and "CDS end" refer to sites homologous to ancestral start and stop codons, respectively.
The location of only one mutation is indicated but additional mutations are present in some of these genes.
CDS, coding sequence; GSDMA, gasdermin A; GSDMB, gasdermin B; GSDMC, gasdermin C; GSDMD, gasdermin D; GSDME, gasdermin E; n.a., not applicable.

Supplementary Table S3. Sequences of primers used for quantitative real time PCRs.

Gene	mRNA (GenBank accession number)	Sense primer	Antisense primer
B2M	NM_004048.2	5'-gatgagtatgcctgccgtgtg-3'	5'-caatccaaatgcggcatct-3'
CASP1	NM_033292.3	5'-caagaatatgcctgttcctgtga-3'	5'-gcccacagacattcatacagttt-3'
CASP14	NM_012114.2	5'-gaatctcttcgaggccctga-3'	5'-atcatgtcggtaggcgatgt-3'
GSDMA	NM_178171.4	5'-cttctccctccccttcttcg-3'	5'-caggagggaaggtttgcatg-3'
GSDMB	NM_001042471.1	5'-agggcttccaccatcagaaa-3'	5'-gatggccctgagagatctgg-3'
GSDMC	NM_031415.2	5'-tccaaccctcttcaatgcct-3'	5'-atcctttgagagctgagcca-3'
GSDMD	NM_024736.6	5'-gtgtacgtggtgactgaggt-3'	5'-cctctgcttcttatccggga-3'
GSDME	NM_004403.2	5'-ggaggatgggaatgtcacca-3'	5'-tcttgttctcgaagccacct-3'
IL1A	NM_000575.4	5'-catgaaggctgcatggatca-3'	5'-tgctgacctaggcttgatga-3'
IL1B	NM_000576.2	5'-ggagaatgacctgagcacct-3'	5'-ggaggtggagagctttcagt-3'
IL1RN	NM_173842.2	5'-aagatgtgcctgtcctgtgt-3'	5'-gcatattggtgaggctgacg-3'
IL18	NM_001562.3	5'-caattgcatcaactttgtggc-3'	5'-taaatatggtccggggtgca-3'
IL33	NM_033439.3	5'-tgagtctcaacacccctcaa-3'	5'-ctggtctggcagtggttttt-3'
IL36A	NM_014440.2	5'-atgtgtgctaaagtcgggga-3'	5'-agtagtgttggctttcccca-3'
IL36B	NM_014438.4	5'-ggcagcacccaaatcctatg-3'	5'-tgccctgaatttctgcacag-3'
IL36G	NM_019618.3	5'-cagcccacattgcagctaaa-3'	5'-tgatgggctggtctctcttg-3'
IL36RN	NM_012275.2	5'-aaggactcggcattgaaggt-3'	5'-ggcaccaagatagagctcca-3'
IL37	NM_014439.3	5'-atccttgagctcagcctctg-3'	5'-gcccacctgagccctataaa-3'
IL38 (IL1F10)	NM_032556.5	5'-accaaggtccccattttcct-3'	5'-tctggaagaaggtgaagcgt-3'
NLRP1	NM_033004.3	5'-ctgcaaactcatacgcctgg-3'	5'-tgggaagatcttgctcacgt-3'
NLRP10	NM_176821.3	5'-ggaggctgtgaaagttgtcc-3'	5'-atctgccattgactcctgct-3'
PYCARD	NM_013258.4	5'-tggtcagcttctacctggag-3'	5'-cagccactcaacgtttgtga-3'
PYDC1	NM_152901.3	5'-tcgggcagctagatatcgtg-3'	5'-acactgctgttgtcttacgc-3'
